# MR-LDP: a two-sample Mendelian randomization for GWAS summary statistics accounting for linkage disequilibrium and horizontal pleiotropy

**DOI:** 10.1093/nargab/lqaa028

**Published:** 2020-05-04

**Authors:** Qing Cheng, Yi Yang, Xingjie Shi, Kar-Fu Yeung, Can Yang, Heng Peng, Jin Liu

**Affiliations:** 1 Centre for Quantitative Medicine, Health Services & Systems Research, Duke-NUS Medical School, Singapore 169857, Singapore; 2 Department of Statistics, Nanjing University of Finance and Economics, Nanjing, 210023, China; 3 Department of Mathematics, The Hong Kong University of Science and Technology, Kowloon, Hong Kong; 4 Department of Mathematics, Hong Kong Baptist University, Kowloon, Hong Kong

## Abstract

The proliferation of genome-wide association studies (GWAS) has prompted the use of two-sample Mendelian randomization (MR) with genetic variants as instrumental variables (IVs) for drawing reliable causal relationships between health risk factors and disease outcomes. However, the unique features of GWAS demand that MR methods account for both linkage disequilibrium (LD) and ubiquitously existing horizontal pleiotropy among complex traits, which is the phenomenon wherein a variant affects the outcome through mechanisms other than exclusively through the exposure. Therefore, statistical methods that fail to consider LD and horizontal pleiotropy can lead to biased estimates and false-positive causal relationships. To overcome these limitations, we proposed a probabilistic model for MR analysis in identifying the causal effects between risk factors and disease outcomes using GWAS summary statistics in the presence of LD and to properly account for horizontal pleiotropy among genetic variants (MR-LDP) and develop a computationally efficient algorithm to make the causal inference. We then conducted comprehensive simulation studies to demonstrate the advantages of MR-LDP over the existing methods. Moreover, we used two real exposure–outcome pairs to validate the results from MR-LDP compared with alternative methods, showing that our method is more efficient in using all-instrumental variants in LD. By further applying MR-LDP to lipid traits and body mass index (BMI) as risk factors for complex diseases, we identified multiple pairs of significant causal relationships, including a protective effect of high-density lipoprotein cholesterol on peripheral vascular disease and a positive causal effect of BMI on hemorrhoids.

## INTRODUCTION

Epidemiological studies have contributed tremendously to improving our understanding of the primary causes of complex diseases. However, numerous cases of significant associations from observational studies have been subsequently contradicted by large clinical trials (1,2). Drawing causal inferences from observational studies is particularly challenging because of unmeasured confounding, reverse causation and selection bias (3,4). Although the randomized controlled trial (RCT) is considered a gold standard for evaluating causality in an exposure–outcome pair, RCTs have certain limitations, including impracticality (no intervention may exist), high expense and ethical issues (5). Fortunately, as germline genetic variants (single-nucleotide polymorphisms, SNPs) are fixed after random mating and cannot be modified by subsequent factors, e.g. environment factors and living styles, Mendelian randomization (MR) uses genetic variants as instruments to examine the causal effects between health risk factors and disease outcomes, largely excluding the influence from unobserved confounding factors (3). In the past decade, a large number of genome-wide association studies (GWAS) have been successfully used to identify genetic variants associated with complex traits at the genome-wide significance level, including both health factors and diseases, e.g. lipids, body mass index (BMI) and type 2 diabetes (T2D), and most of completed GWAS are only observational studies instead of RCTs. The results from completed GWAS are mostly publicly accessible; e.g. GWAS Catalog outlines a list of sources for summary statistics (https://www.ebi.ac.uk/gwas/downloads/summary-statistics). This large amount of publicly available GWAS summary statistics has prompted the widespread use of two-sample MR as an efficient and cost-effective method to interrogate the causal relationships among health risk factors and disease outcomes.

MR is closely related to the instrumental variable (IV) methods, which have a long history of use in econometrics (6). Classically, an inverse-variance weighted (IVW) and a likelihood-based approach have been used for two-sample MR analysis with summary-level data (7). Despite their successes, they have several limitations. First, they must obey the assumption that IVs affect the outcome exclusively through the risk exposures, which is also referred to as exclusion restriction assumption or no horizontal pleiotropy. The violation of this assumption can distort the statistical inference for MR analysis, leading to biased estimates and false-positive causal relationships. Recent comprehensive surveys reported pervasive pleiotropy among complex traits (8,9), such as autoimmune diseases (10) and psychiatric disorders (11). Consequently, methods that do not account for pleiotropy can substantially reduce the power and inflate the false-positive discoveries. To address this issue, sisVIVE was proposed in the presence of individual-level data (12). To further relax this assumption for two-sample MR analysis using summary-level data, various statistical methods have been proposed, and we divide them into two categories. The first group consists of stepwise methods to correct the impact of horizontal pleiotropy. These methods first detect and remove SNPs with horizontal pleiotropy, and MR analysis is performed in the subsequent step, including *Q* test (13), Cook’s distance (14), studentized residuals (14), GSMR (15) and MR-PRESSO (16). The drawback of these methods is that the number of SNPs after removal is limited, given that abundant pleiotropy exists among complex traits, which can substantially reduce the statistical power to detect causal relationships. In contrast, the second group of methods jointly estimate causal effects by taking into account the horizontal pleiotropy, e.g. MR-Egger (17), MRMix (18) and RAPS (19). RAPS addresses measurement error issues, while MR-Egger and other existing methods applicable to GWAS summary statistics assume that sampling error from SNP exposure is negligible (20).

On the other hand, the classical MR methods (e.g. IVW, MR-Egger) only work for independent IVs and further assume no measurement errors. Among the methods mentioned earlier, only GSMR is capable of accounting weak or moderate linkage disequilibrium (LD) among SNPs, while others demand all-instrumental SNPs to be independent, which is typically achieved by SNP pruning and thus reducing the number of instrumental variants for follow-up MR analysis. As SNPs in close proximity tend to be highly correlated, MR methods that do not account for LD structure may substantially lose statistical power due to the pruning process. Moreover, GSMR is a stepwise method that removes instrumental variants with horizontal pleiotropy, making it less powerful due to the removal of invalid variants.

In this paper, we propose a statistically unified and efficient two-sample MR method to utilize all weak instruments within LD (MR-LD), and further consider an MR-LD accounting for horizontal pleiotropy (MR-LDP). Similar to RAPS, MR-LDP does not require any measurement error assumption. The key idea is to build a joint probabilistic model for GWAS summary statistics from both exposure and outcome using a reference panel to reconstruct LD among instrumental variants and to conduct a formal hypothesis test to make inferences about the causal effect that links the exposure and the outcome through a linear relationship. We also develop an efficient variational Bayesian expectation-maximization (EM) algorithm accelerated by using the parameter expansion (PX-VBEM) to estimate the causal effect for MR-LD and MR-LDP. Moreover, we calibrate the evidence lower bound (ELBO) to construct the likelihood ratio test for the evaluation of the statistical significance of the estimated effect. Simulation studies show that MR-LDP outperforms competing methods in terms of type I error control and point estimates for making the causal inference. Additionally, we used two real exposure–outcome pairs to validate results from MR-LD and MR-LDP compared with alternative methods, particularly showing our methods more efficiently use all SNPs in LD. By further applying MR-LDP to summary statistics from GWAS, we identified multiple pairs of significant causal relationships, including a protective effect of high-density lipoprotein cholesterol (HDL-C) on the peripheral vascular disease (PVD) and a positive causal effect of BMI on hemorrhoids.

## MATERIALS AND METHODS

### Reference panel data

As MR-LD and MR-LDP use the marginal effect sizes and their standard errors from GWAS summary statistics to build a probabilistic model for making a causal inference, information regarding correlations among SNPs is missing; i.e. LD denoted as **R** is missing. Thus, we need to use a reference panel dataset to assist with reconstructing LD. In this study, given that we primarily focus on European populations, we choose to use samples from the following resource as the external reference panel: UK10K Project (Avon Longitudinal Study of Parents and Children, ALSPAC; TwinsUK) merged with 1000 Genome Project Phase 3 (*N* = 4284), which is denoted UK10K thereafter. As SNPs from HapMap Project Phase 3 (HapMap3) are more reliable, we choose to limit our analysis using SNPs from HapMap3 (*P* = 1 189 556).

As samples from ALSPAC and TwinsUK include populations other than Europeans, we conducted strict quality control for UK10K data using PLINK (21). First, SNPs were excluded from the analysis if their calling rates were <95%, minor allele frequencies were <0.01 or *P*-values were <1 × 10^−6^ in the Hardy–Weinberg equilibrium test. We then removed the individuals with genotype missing rates >5%. To further remove individuals with high relatedness in all samples, we used GCTA (22) to first identify those individual pairs with estimated genetic relatedness >0.05 and then randomly remove one from each pair. Additionally, we carried out the principal component analysis on the individuals to identify the population stratification (23). In this way, we extracted the clustering subgroup representing the primary European ancestry using hierarchical clustering on principal component approach (24). After data pre-processing, there were 3764 individuals remaining with 989 932 SNPs.

### Choice of LD matrix

Since the LD between two SNPs decays exponentially concerning their distance, we use LDetect (25) to partition the whole genome into *L* blocks first and then calculate the estimated correlation matrix in each block. For each block, we adopt a shrinkage method to guarantee the sparsity and positive definiteness of the estimated correlation matrix (26). In particular, the correlation matrix estimator $\widehat{{\mathbf {R}}}^{(l)}$ in each block is obtained by optimizing as follows:

(1) $$\begin{eqnarray*} \nonumber \widehat{{\mathbf {R}}}^{(l)} & = &\textrm{arg min}_{{\mathbf {R}}^{(l)} \succ 0} (\Vert {\mathbf {R}}^{(l)} - \widehat{{\mathbf {R}}}^{(l)}_{\mathrm{emp}} \Vert _{\rm F}^2/ 2 - \tau \log |{\mathbf {R}}^{(l)} | \nonumber\\ &&+ \lambda \Vert {\mathbf {R}}^{(l)-}\Vert _1), \ \ \ \end{eqnarray*}$$

where $\widehat{{\mathbf {R}}}^{(l)}_{\mathrm{emp}}$ is the empirical correlation matrix in the *l*th block, *λ* ≥ 0 is the shrinkage tuning parameter and the lasso-type penalty ensures a sparse solution. In addition, *τ* > 0 is fixed at a small value, and the logarithmic barrier term is used to enforce a positive-definite solution. More details can be found in (26). A corresponding R package named *PDSCE* is available to complete the estimation process. In addition, we fixed the shrinkage parameter *λ* to be 0.055 in simulation studies and vary *λ* ∈ {0.1, 0.15} in real data analysis.

### Likelihood for summary statistics

Before elaborating on our method, we first review the following multiple linear regression model that links a trait to genotype data:

$$\begin{eqnarray*} {\mathbf {y}}= {\mathbf {G}}\boldsymbol{\gamma }+ \boldsymbol{\epsilon }, \end{eqnarray*}$$

where **y** is an *n* × 1 vector for trait among *n* individuals, **G** is an *n* × *p* matrix for genotypes, $\boldsymbol{\gamma }$ is a *p* × 1 vector for effect sizes and $\boldsymbol{\epsilon }$ is the vector for random noises. Suppose that the individual-level data {**G**, **y**} are not accessible, but the summary statistics $\lbrace \widehat{\boldsymbol{\gamma }}_k, \widehat{{\mathbf {s}}}_k^2\rbrace _{k = 1, \ldots ,p}$ from univariate linear regression are available:

$$\begin{eqnarray*} \widehat{\boldsymbol{\gamma }}_k = ({\mathbf {g}}_k^{\rm{{T}}}{\mathbf {g}}_k )^{-1}{\mathbf {g}}_k^{\rm{{T}}}{\mathbf {y}}, \ \ \ \widehat{{\mathbf {s}}}_k^2 = (n {\mathbf {g}}_k^{\rm{{T}}}{\mathbf {g}}_k)^{-1}({\mathbf {y}}- {\mathbf {g}}_k \widehat{\boldsymbol{\gamma }}_k)^{\rm{{T}}}({\mathbf {y}}- {\mathbf {g}}_k \widehat{\boldsymbol{\gamma }}_k), \end{eqnarray*}$$

where **g**_*k*_ is the *k*th column of **G**, and $\widehat{\boldsymbol{\gamma }}_k$ and $\widehat{{\mathbf {s}}}_k^2$ are the estimated effect sizes and their variance for SNP *k*, respectively. $\widehat{{\mathbf {R}}}$ denotes the correlation among all genotyped SNPs and $\widehat{{\mathbf {S}}}= \text{diag}([\widehat{{\mathbf {s}}}_{1}, \ldots , \widehat{{\mathbf {s}}}_{p}] )$ is a diagonal matrix for corresponding standard errors. Provided that sample size *n* is large enough and the trait is highly polygenic (i.e. the squared correlation coefficient between the trait and each genetic variant is close to zero), we can use the following formula to approximate the distribution of $\boldsymbol{\gamma }$ based on the summary statistics in a similar fashion as (27–30):

(2) $$\begin{eqnarray*} \widehat{\boldsymbol{\gamma }}| \boldsymbol{\gamma }, \widehat{{\mathbf {R}}}, \widehat{{\mathbf {S}}}\sim \mbox{ $\mathcal {N}$}(\widehat{{\mathbf {S}}}\widehat{{\mathbf {R}}}\widehat{{\mathbf {S}}}^{-1} \boldsymbol{\gamma }, \widehat{{\mathbf {S}}}\widehat{{\mathbf {R}}}\widehat{{\mathbf {S}}}), \end{eqnarray*}$$

where $\widehat{\boldsymbol{\gamma }}= [\widehat{\gamma }_1,\dots ,\widehat{\gamma }_p]^{\rm T}$. Analogously, we apply this distribution to the two-sample MR analysis. The summary statistics for SNP exposure and SNP outcome are denoted by $\lbrace \widehat{\gamma }_k, \widehat{{\mathbf {s}}}_{\gamma _k}^2 \rbrace _{k = 1, \ldots , p}$ and $\lbrace \widehat{\Gamma }_k, \widehat{{\mathbf {s}}}_{\Gamma _k}^2 \rbrace _{k = 1, \ldots , p}$, respectively. Therefore, the likelihood for two-sample summary statistics can be written as

(3) $$\begin{eqnarray*} \widehat{\boldsymbol{\gamma }}| \boldsymbol{\gamma }, \widehat{{\mathbf {R}}}, \widehat{{\mathbf {S}}}_{\gamma } &\sim &\mbox{ $\mathcal {N}$}(\widehat{{\mathbf {S}}}_{\gamma } \widehat{{\mathbf {R}}}\widehat{{\mathbf {S}}}_{\gamma }^{-1} \boldsymbol{\gamma }, \widehat{{\mathbf {S}}}_{\gamma } \widehat{{\mathbf {R}}}\widehat{{\mathbf {S}}}_{\gamma }) ,\nonumber \\ \widehat{\boldsymbol{\Gamma }}| \boldsymbol{\Gamma }, \widehat{{\mathbf {R}}}, \widehat{{\mathbf {S}}}_{\Gamma } &\sim &\mbox{ $\mathcal {N}$}(\widehat{{\mathbf {S}}}_{\Gamma } \widehat{{\mathbf {R}}}\widehat{{\mathbf {S}}}_{\Gamma }^{-1} \boldsymbol{\Gamma },\widehat{{\mathbf {S}}}_{\Gamma } \widehat{{\mathbf {R}}}\widehat{{\mathbf {S}}}_{\Gamma }) , \end{eqnarray*}$$

where $\widehat{{\mathbf {S}}}_{\gamma } = \text{diag}([\widehat{{\mathbf {s}}}_{\gamma _1}, \ldots , \widehat{{\mathbf {s}}}_{\gamma _p}] )$ and $\widehat{{\mathbf {S}}}_{\Gamma }= \text{diag}([\widehat{{\mathbf {s}}}_{\Gamma _1}, \ldots , \widehat{{\mathbf {s}}}_{\Gamma _p}] )$ are both diagonal matrices, and $\widehat{\boldsymbol{\Gamma }}= [\widehat{\Gamma }_1,\dots ,\widehat{\Gamma }_p]^{\rm T}$. In this formulation, the correlations among all *p* SNPs, $\widehat{{\mathbf {R}}}$, are not estimable from summary statistics itself. Zhu and Stephens (29) showed that $\widehat{{\mathbf {R}}}$ could be replaced with $\widehat{{\mathbf {R}}}^{\mathrm{ref}}$ that is estimated from independent samples, where the difference in log-likelihood between individual-level data and summary statistics is a constant that does not depend on the effect size assuming that polygenicity holds and the sample size of individual-level data is large. Thus, the distributions for summary statistics [Equation (3)] will produce approximately the same inferential results as its counterpart for individual-level data. Hereafter, we use $\widehat{{\mathbf {R}}}$ implicitly for $\widehat{{\mathbf {R}}}^{\mathrm{ref}}$ and details on estimating $\widehat{{\mathbf {R}}}$ can be found in the ‘Choice of LD matrix’ section.

### MR-LDP model overview

**Figure 1.**
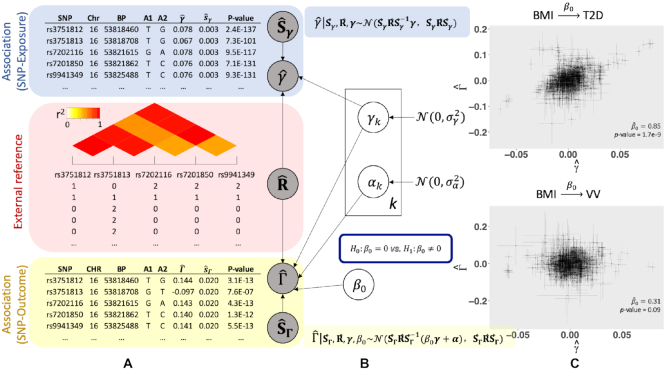
MR-LDP model overview. (**A**) Inputs for MR-LDP include GWAS summary statistics from both the risk factor (blue) and the disease outcome (yellow), and an external reference panel data (red). (**B**) A probabilistic graphical model representation of MR-LDP. The box is the ‘plate’ representing SNPs, *k* = 1, …, *p*. The circles are either variables or parameters. The circle outside the block is the primary parameter of interest. The variables in shaded circles are observed (i.e. GWAS summary statistics $\lbrace \widehat{\gamma }_k, \widehat{{\mathbf {s}}}_{\gamma _k} \rbrace _{k = 1, \ldots , p}$ and $\lbrace \widehat{\Gamma }_k, \widehat{{\mathbf {s}}}_{\Gamma _k} \rbrace _{k = 1, \ldots , p}$, and the estimated $\widehat{{\mathbf {R}}}$ for these *p* SNPs from a reference panel) and variables in unshaded circles are latent variables (i.e. *γ*_*k*_ and *α*_*k*_, *k* = 1, …, *p*). The primary goal is to conduct a formal hypothesis testing for $\mathcal {H}_0: \beta _0=0\; \mathrm{versus}\; \mathcal {H}_1: \beta _0\ne 0$. (**C**) Scatter plots of effect sizes with their standard errors for two exposure–outcome pairs: BMI-T2D and BMI-VV; T2D for type 2 diabetes and VV for varicose veins. Dots represent the effect sizes from SNP exposure against these from SNP outcome, and horizontal and vertical bars represent the standard errors from SNP exposure and SNP outcome, respectively. The estimated *β*_0_ and their *P*-values from MR-LDP are shown in each part.

The fundamental assumptions for two-sample MR analysis include the independence among IVs, and three IV assumptions for a genetic instrument: (i) associated with health risk factors (*γ* ≠ 0); (ii) independent of unobserved confounding factors between the risk factors and the disease outcomes; and (iii) independent of the outcome given risk factors and confounders. Given the strong LD structure among SNPs and abundant horizontal pleiotropy in GWAS, these unique features invalidate the independence assumption for genetic variants and IV assumption (iii). Our proposed MR-LDP aims to make the causal inference of the risk factors on a disease outcome using a probabilistic model by accounting for both the LD structure and the influence of horizontal pleiotropy, as depicted in Figure 1. We first utilize an approximated likelihood to depict the distribution of correlated SNPs from GWAS summary statistics for the risk exposure and the disease outcome, respectively, as shown in Equation (3). Given *p* instrumental variants, the inputs for MR-LDP are GWAS summary statistics for SNP exposure and SNP outcome, respectively, and a genotype reference panel (Figure 1A). By introducing an additional random effect of $\boldsymbol{\alpha }$, we would further eliminate the variance in the disease outcome due to pervasive horizontal pleiotropy. Since MR-LDP uses an approximated likelihood to jointly delineate the distribution for summary statistics (i.e. estimated effect sizes and their standard errors) from GWAS, it is free of the assumption for no measurement errors, requiring that sample sizes used to generate GWAS summary statistics are large (20,31). Figure 1B depicts MR-LDP as a probabilistic graphical model, where the observed variables of our model include GWAS summary statistics from both the SNP exposure and the SNP outcome, and an external reference panel for genotype data. We assume that *α*_*k*_ and *γ*_*k*_ follow two independent Gaussian distributions. The latent variable *γ*_*k*_ and parameter *β*_0_ jointly assist with formulating the distribution for SNP outcome. Then, we can formalize the hypothesis testing for *β*_0_, as shown in Figure 1B. The scatter plots of estimated effect sizes for SNP exposure against SNP outcome, together with the MR-LDP analysis results ($\hat{\beta }_0$ and *P*-value), are shown in Figure 1C. In both BMI-T2D and BMI-VV, there is a dominant proportion of instrumental variants in the center that is mainly due to LD, and methods that do not account for LD tend to inflate findings.

### Details of MR-LDP

#### Parameterization for causal relationship

The relationship between $\boldsymbol{\gamma }$ and $\boldsymbol{\Gamma }$ can be constructed using linear structural models as follows:

(4) $$\begin{eqnarray*} \Gamma _j = \beta _0\gamma _j \quad \mathrm{or} \quad \Gamma _j = \alpha _j +\beta _0\gamma _j , \end{eqnarray*}$$

where *j* = 1, …, *p*, considering without/with horizontal pleiotropy, respectively (12,32). Note that *β*_0_ is the effect size of the exposure on the outcome and $\boldsymbol{\alpha }= [\alpha _1,\dots ,\alpha _p]^{\rm T}$ is the vector of effects of genetic variants on the outcome due to horizontal pleiotropy. Importantly, *β*_0_ can be interpreted as the causal effect between exposure and outcome in the study (32). More details regarding linear structural models incorporating the relationship provided in Equation (4) are available in the Supplementary Data. As MR-LD can be taken as a special case of MR-LDP by taking all $\boldsymbol{\alpha }$ to be zero, we focus on deriving MR-LDP in the main text and provide the Supplementary Data for details on MR-LD.

#### Empirical Bayes model

By assuming that $\boldsymbol{\gamma }$ and $\boldsymbol{\alpha }$ are two latent variables coming from two independent Gaussian distributions, the complete-data likelihood can be written as follows:

(5) $$\begin{eqnarray*} \nonumber &&\Pr (\widehat{\boldsymbol{\Gamma }}, \widehat{\boldsymbol{\gamma }}, \boldsymbol{\gamma }, \boldsymbol{\alpha }| \widehat{{\mathbf {S}}}_{\boldsymbol{\gamma }}, \widehat{{\mathbf {S}}}_{\boldsymbol{\Gamma }}, \widehat{{\mathbf {R}}}; \boldsymbol{\theta }) \\ &&= \mbox{Pr}(\widehat{\boldsymbol{\Gamma }}| \boldsymbol{\gamma }, \boldsymbol{\alpha }, \widehat{{\mathbf {R}}}, \widehat{{\mathbf {S}}}_{\Gamma }; \beta _0)\mbox{Pr}(\widehat{\boldsymbol{\gamma }}| \boldsymbol{\gamma }, \widehat{{\mathbf {R}}}, \widehat{{\mathbf {S}}}_{\gamma })\mbox{Pr}(\boldsymbol{\alpha }|\sigma ^2_{\boldsymbol{\alpha }})\mbox{Pr}(\boldsymbol{\gamma }|\sigma ^2_{\boldsymbol{\gamma }}), \nonumber\\ \end{eqnarray*}$$

where $\boldsymbol{\theta }\stackrel{{\textrm {def}}}{=}\lbrace \beta _0, \sigma _{\boldsymbol{\gamma }}^2, \sigma _{\boldsymbol{\alpha }}^2\rbrace$ denotes the collection of model parameters. Integrating out the latent variables $\boldsymbol{\gamma }$ and $\boldsymbol{\alpha }$, the marginal likelihood can be written as

$$\begin{eqnarray*} \Pr (\widehat{\boldsymbol{\Gamma }}, \widehat{\boldsymbol{\gamma }}|\widehat{{\mathbf {S}}}_{\boldsymbol{\gamma }}, \widehat{{\mathbf {S}}}_{\boldsymbol{\Gamma }}, \widehat{{\mathbf {R}}}; \boldsymbol{\theta }) = \iint \Pr (\widehat{\boldsymbol{\Gamma }}, \widehat{\boldsymbol{\gamma }}, \boldsymbol{\alpha }, \boldsymbol{\gamma }| \widehat{{\mathbf {S}}}_{\boldsymbol{\gamma }}, \widehat{{\mathbf {S}}}_{\boldsymbol{\Gamma }}, \widehat{{\mathbf {R}}}; \boldsymbol{\theta }) {\rm d}\boldsymbol{\gamma }\, {\rm d}\boldsymbol{\alpha }. \ \ \ \ \ \end{eqnarray*}$$

#### Algorithm

The standard EM algorithm is a common choice to find the maximum likelihood for probabilistic models in the presence of latent variables (33). The conventional EM algorithm involves the inverse of large matrix $\widehat{{\mathbf {R}}}$ that is at order *O*(*p*^3^) computationally, making it computational infeasible when a large number of instrumental variants are used. To address this issue, we develop an accelerated variational Bayes (VB) EM algorithm in light of (34), namely, PX-VBEM. Starting with the algorithm, we expand the original MR-LD/MR-LDP model [Equation (5)] as follows:

(6) $$\begin{eqnarray*} \widehat{\boldsymbol{\gamma }}| \boldsymbol{\gamma }, \widehat{{\mathbf {R}}}, \widehat{{\mathbf {S}}}_{\boldsymbol{\gamma }} &\sim &\mbox{ $\mathcal {N}$}(\xi \widehat{{\mathbf {S}}}_{\boldsymbol{\gamma }} \widehat{{\mathbf {R}}}\widehat{{\mathbf {S}}}_{\boldsymbol{\gamma }}^{-1} \boldsymbol{\gamma }, \widehat{{\mathbf {S}}}_{\boldsymbol{\gamma }} \widehat{{\mathbf {R}}}\widehat{{\mathbf {S}}}_{\boldsymbol{\gamma }}). \end{eqnarray*}$$

Next, we sketch the VBEM algorithm using the parameter expanded in Equation (6) for MR-LDP and algorithmic details for MR-LD can be found in the Supplementary Data. The model parameters for MR-LDP after parameter expansion become $\boldsymbol{\theta }= \lbrace \beta _0, \sigma _{\boldsymbol{\gamma }}^2 , \sigma _{\boldsymbol{\alpha }}^2 , \xi \rbrace$. Given variational posterior distribution $q(\boldsymbol{\gamma },\boldsymbol{\alpha })$, it is straightforward to evaluate the marginal log-likelihood by decomposing it into two parts, the ELBO and the Kullback–Leibler (KL) divergence, which is denoted as follows:

(7) $$\begin{eqnarray*} \log \mbox{Pr}(\widehat{\boldsymbol{\gamma }},\widehat{\boldsymbol{\Gamma }}|\widehat{{\mathbf {S}}}_{\boldsymbol{\gamma }},\widehat{{\mathbf {S}}}_{\boldsymbol{\Gamma }}, \widehat{{\mathbf {R}}}; \boldsymbol{\theta }) = \mathcal {L}(q) + \mathbb {KL}(q\Vert p), \end{eqnarray*}$$

where

(8) $$\begin{eqnarray*} \nonumber \mathcal {L}(q) &=& \iint \limits _{\boldsymbol{\gamma },\boldsymbol{\alpha }} q(\boldsymbol{\gamma },\boldsymbol{\alpha }) \log \frac{\mbox{Pr}(\widehat{\boldsymbol{\gamma }},\widehat{\boldsymbol{\Gamma }},\boldsymbol{\gamma },\boldsymbol{\alpha }|\widehat{{\mathbf {S}}}_{\boldsymbol{\gamma }},\widehat{{\mathbf {S}}}_{\boldsymbol{\Gamma }}, \widehat{{\mathbf {R}}}; \boldsymbol{\theta }) }{q(\boldsymbol{\gamma },\boldsymbol{\alpha })} {\rm d}\boldsymbol{\gamma }\, {\rm d}\boldsymbol{\alpha }, \\ \mathbb {KL}(q\Vert p) &=& \iint \limits _{\boldsymbol{\gamma },\boldsymbol{\alpha }} q(\boldsymbol{\gamma },\boldsymbol{\alpha }) \log \frac{q(\boldsymbol{\gamma },\boldsymbol{\alpha }) }{p(\boldsymbol{\gamma },\boldsymbol{\alpha }|\widehat{\boldsymbol{\gamma }},\widehat{\boldsymbol{\Gamma }},\widehat{{\mathbf {S}}}_{\boldsymbol{\gamma }},\widehat{{\mathbf {S}}}_{\boldsymbol{\Gamma }}, \widehat{{\mathbf {R}}}; \boldsymbol{\theta }) }{\rm d}\boldsymbol{\gamma }\, {\rm d}\boldsymbol{\alpha }, \ \ \ \ \ \end{eqnarray*}$$

where $\mathcal {L}(q)$ is the ELBO of the marginal log-likelihood and $\mathbb {KL}(q\Vert p)$ is the KL divergence between two distributions. Moreover, $\mathbb {KL}(q\Vert p) \ge 0$ with equality holding if and only if the variational posterior distribution (*q*) is equal to the true posterior distribution (*p*). As a consequence, minimizing the KL divergence is equivalent to maximizing ELBO. Compared with the standard EM algorithm, the crux of VBEM is to optimize *q* within a factorizable family of distributions by the mean-field assumption (35), which assumes that $q(\boldsymbol{\gamma },\boldsymbol{\alpha })$ can be factorized as

(9) $$\begin{eqnarray*} q(\boldsymbol{\gamma }, \boldsymbol{\alpha }) = \prod _{j=1}^p q_{\gamma _j}(\gamma _j) \prod _{k = 1}^p q_{\alpha _k}(\alpha _k). \end{eqnarray*}$$

This only assumption in variational inference promotes computational efficiency and scalability in large-scale computational problems given that a coordinate descent algorithm is commonly used to identify the optimal distribution q*. To briefly show this, we first note that this factorization [Equation (9)] is used as an approximation for the posterior distribution $p(\boldsymbol{\gamma },\boldsymbol{\alpha }|\widehat{\boldsymbol{\gamma }},\widehat{\boldsymbol{\Gamma }},\widehat{{\mathbf {S}}}_{\boldsymbol{\gamma }},\widehat{{\mathbf {S}}}_{\boldsymbol{\Gamma }}, \widehat{{\mathbf {R}}}; \boldsymbol{\theta })$. In the VB E-step, given the latent variables $\boldsymbol{\gamma }_{-k}$ and $\boldsymbol{\alpha }$, the terms with *γ*_*k*_ have a quadratic form, where $\boldsymbol{\gamma }_{-k}$ is the $\boldsymbol{\gamma }$ vector removing the *k*th element. Similarly, when all other latent variables are fixed, we can show that the terms with *α*_*k*_ also take a quadratic form. Thus, the variational posterior distributions for *γ*_*k*_ and *α*_*k*_ are both from Gaussian distributions, $\mathcal {N}(\mu _{\gamma _k},\sigma ^2_{\gamma _k})$ and $\mathcal {N}(\mu _{\alpha _k},\sigma ^2_{\alpha _k})$, respectively, where we call $\lbrace \mu _{\gamma _k},\sigma ^2_{\gamma _k}, \mu _{\alpha _k},\sigma ^2_{\alpha _k}\rbrace _{k=1,\dots ,p}$ variational parameters. The details of derivations for updating these variational parameters and the ELBO $\mathcal {L}(q)$ in the marginal log-likelihood [Equation (7)] at the old parameter $\boldsymbol{\theta }^{{\rm old}}$ can be found in the Supplementary Data. After updating variational parameters in the VB E-step, model parameters ($\boldsymbol{\theta }$) can be updated by setting the derivative of the ELBO to zero. Derivation details can be found in the Supplementary Data, where we summarize the PX-VBEM algorithms for MR-LD and MR-LDP in Algorithms 1 and 2, respectively.

#### Inference for causality

We can easily formulate the problem provided in Equation (5) as a statistical test for the null hypothesis that the health risk factor is not associated with the disease of interest, or $\mathcal {H}_0: \beta _0 = 0$. Testing this hypothesis requires evaluating the marginal log-likelihood of observed data in MR-LD or MR-LDP, similar to what has been done previously in (36,37); details are given in the Supplementary Data. As VB searches within a factorizable family for the posterior distribution, one can only obtain an approximation for the posterior distribution of latent variables. Earlier works showed that VBEM provides useful and accurate posterior mean estimates (38). Despite its computational efficiency and accuracy for estimating the posterior mean, VB suffers from underestimating the variance of the target distribution (25,39–40). Thus, the ELBO from the VB-type algorithm cannot be directly used to conduct a likelihood-based test. In this paper, we follow Yang *et al.* (37) and adopt the similar strategy to calibrate ELBO as well as mitigate the bias of variance. Details for the PX-VBEM algorithm and the calibration of ELBO can be found in the Supplementary Data.

#### Relationship between MR-LD and TWAS

Using transcriptome data as risk factors, MR-LD can be viewed as a TWAS-type analysis using summary-level data from both expression quantitative trait loci (eQTL) and GWAS, where eQTL and GWAS summary statistics are used as SNP exposure and SNP outcome in the analysis, respectively. Since TWAS-type analysis only seeks genes that are significantly associated with the outcome of interest at the genome-wide level, one cannot infer causality without excluding other potential associations, e.g. horizontal pleiotropy. We note that PMR-Egger (41) was recently proposed to calibrate the type I error control by using a burden test assumption to infer causal relationship. However, this assumption depends heavily on the fact that all effect sizes from horizontal pleiotropy are the same. Therefore, MR-LDP can also be viewed as a relaxation of the burden assumption, which makes it more powerful in accounting for horizontal pleiotropy with more general patterns.

## RESULTS

### Simulations

#### Methods for comparison

We compared the performance of five methods in the main text: (i) our MR-LD and MR-LDP implemented in the R package *MR.LDP*; (ii) GSMR implemented in the R package *gsmr*; (iii) RAPS implemented in the R package *mr.raps*; (iv) IVW implemented in the R package *MendelianRandomization*; and (v) MR-Egger implemented in the R package *MendelianRandomization*. All methods were used with default settings. Note that we performed GSMR analysis with removing outliers first when there exists horizontal pleiotropy ($h_{\boldsymbol{\alpha }} ^2\ne 0$). We conducted comprehensive simulation studies to better gauge the performance of each method in simulation studies in terms of type I error control and point estimates.

In simulation studies, we considered genetic instruments both without and with horizontal pleiotropy. In the scenario that genetic instruments have horizontal pleiotropy, we further considered two cases: the sparse and dense horizontal pleiotropy. The sparse horizontal pleiotropy indicates that only a proportion of genetic instruments have direct effects ($\boldsymbol{\alpha }$ is sparse) on the outcome, while the dense horizontal pleiotropy indicates that all genetic instruments have direct effects ($\boldsymbol{\alpha }$ is dense). As GSMR is a stepwise method that first removes invalid instruments, the dense horizontal pleiotropy theoretically implies that all genetic instruments are invalid. To make fair comparisons, we considered the sparse horizontal pleiotropy with sparsity at 0.2 or 0.4. In addition, as RAPS, IVW and MR-Egger tend to inflate type I error in the presence of LD, we conducted SNP pruning for a fair comparison of point estimates.

#### Simulation settings

To make our simulations as realistic as possible, we started by generating the individual-level two-sample data as follows:

$$\begin{eqnarray*} {\mathbf {x}}= {\mathbf {G}}_1\boldsymbol{\gamma }+{\mathbf {U}}_x \boldsymbol{\eta }_x + {\mathbf {e}}_1,\quad {\mathbf {y}}= \beta _0 {\mathbf {x}}+ {\mathbf {G}}_2 \boldsymbol{\alpha }+ {\mathbf {U}}_y\boldsymbol{\eta }_y+ {\mathbf {e}}_2, \end{eqnarray*}$$

where ${\mathbf {G}}_1 \in \mathbb {R}^{n_1\times p}$ and ${\mathbf {G}}_2 \in \mathbb {R}^{n_2\times p}$ were both genotype matrices, ${\mathbf {U}}_x \in \mathbb {R}^{n_1\times q }$ and ${\mathbf {U}}_y \in \mathbb {R}^{n_2\times q}$ were matrices for confounding variables, *n*_1_ and *n*_2_ were the corresponding sample sizes, *p* was the number of genetic variants, ${\mathbf {x}}\in \mathbb {R}^{n_1\times 1}$ was the exposure vector, ${\mathbf {y}}\in \mathbb {R}^{n_2\times 1}$ was the outcome vector and the error terms **e**_1_ and **e**_2_ were obtained from $\mbox{ $\mathcal {N}$}(\bf 0, \sigma _{{\mathbf {e}}_1}^2{\mathbf {I}}_{n_1})$ and $\mbox{ $\mathcal {N}$}(\bf 0, \sigma _{{\mathbf {e}}_2}^2{\mathbf {I}}_{n_2})$, respectively. In this generative model, *β*_0_ was the true causal effect, while $\boldsymbol{\alpha }$ exhibited the direct effects on the disease. We considered two cases: dense and sparse horizontal pleiotropy. For the dense case, we assumed that *α*_*k*_ was independent and identically distributed as $\mbox{ $\mathcal {N}$}(0, \sigma _{\boldsymbol{\alpha }}^2)$. However, for the sparse case, we assumed that only a fraction of *α*_*k*_ was from a Gaussian distribution and remaining were zero. In simulations, we considered sparsity at both 0.2 and 0.4. Note that $\sigma _{\boldsymbol{\alpha }}^2$ was set by controlling the heritability due to horizontal pleiotropy. Moreover, to mimic the real applications where an external reference panel was applied to estimate the correlation among SNPs, another genotype matrix ${\mathbf {G}}_3 \in \mathbb {R}^{n_3\times p}$ was generated as the reference panel data to estimate the correlation matrix, where *n*_3_ was the sample size in the reference panel. We fixed *n*_1_ = *n*_2_ = 20 000 but varied *n*_3_ ∈ {500, 2500, 4000}. In detail, we first generated a data matrix from multivariate normal distribution $\mbox{ $\mathcal {N}$}(\bf 0, \boldsymbol{\Sigma }(\rho ))$, where $\boldsymbol{\Sigma }(\rho )$ is a block autoregressive with *ρ* = 0, 0.4 or 0.8 representing weak, moderate or strong LD, respectively. We then generated minor allele frequencies from a uniform distribution $\mathbb {U}(0.05,0.5)$ and categorized the data matrix into dosage values {0, 1, 2} according to the Hardy–Weinberg equilibrium under the generated minor allele frequencies. The number of blocks was *M* = 10 or 20 and the number of SNPs within each block was 50. Correspondingly, *P* = 500 or 1000. For confounding variables, we sampled each column of **U**_*x*_ and **U**_*y*_ from a standard normal distribution with fixed *q* = 50, while $\boldsymbol{\eta }_x \in \mathbb {R}^{q\times 1}$ and $\boldsymbol{\eta }_y \in \mathbb {R}^{q \times 1}$ were the corresponding coefficients of confounding factors. Each row of $(\boldsymbol{\eta }_x, \boldsymbol{\eta }_y)$ was generated from a multivariate normal distribution $\mbox{ $\mathcal {N}$}(\bf 0, \boldsymbol{\Sigma }_{\eta })$, where $\boldsymbol{\Sigma }_{\eta }$ is a 2× 2 matrix with diagonal elements set as 1 and off-diagonal elements set as 0.8.

We then conducted single-variant analysis to obtain the summary statistics for SNP exposure and SNP outcome, $\lbrace \widehat{\gamma }_k, \widehat{s}_{\boldsymbol{\gamma }k}^2 \rbrace _{k = 1, \ldots , p}$ and $\lbrace \widehat{\Gamma }_k, \widehat{s}_{\boldsymbol{\Gamma }k}^2\rbrace _{k = 1, \ldots , p}$, respectively. In simulations, we controlled the signal magnitude for both $\boldsymbol{\gamma }$ and $\boldsymbol{\alpha }$ using their corresponding heritability, $h_{\boldsymbol{\gamma }}^2 ={\hbox{var}(\beta _0{\mathbf {G}}_1\boldsymbol{\gamma })}/{\hbox{var}({\mathbf {y}})}$ and $h_{\boldsymbol{\alpha }} ^2= {\hbox{var}({\mathbf {G}}_2\boldsymbol{\alpha })}/{\hbox{var}({\mathbf {y}})}$, respectively. Thus, we could control $h_{\boldsymbol{\alpha }} ^2$ and $h_{\boldsymbol{\gamma }}^2$ at any value by controlling confounding variables and the error terms, $\sigma _{{\mathbf {e}}_1}^2$ and $\sigma _{{\mathbf {e}}_2}^2$. In all settings, we fixed $h_{\boldsymbol{\gamma }}^2 = 0.1$ and varied $h_{\boldsymbol{\alpha }} ^2 \in \lbrace 0, 0.05, 0.1\rbrace$.

#### Simulation results: type I error control and point estimates

**Figure 2.**
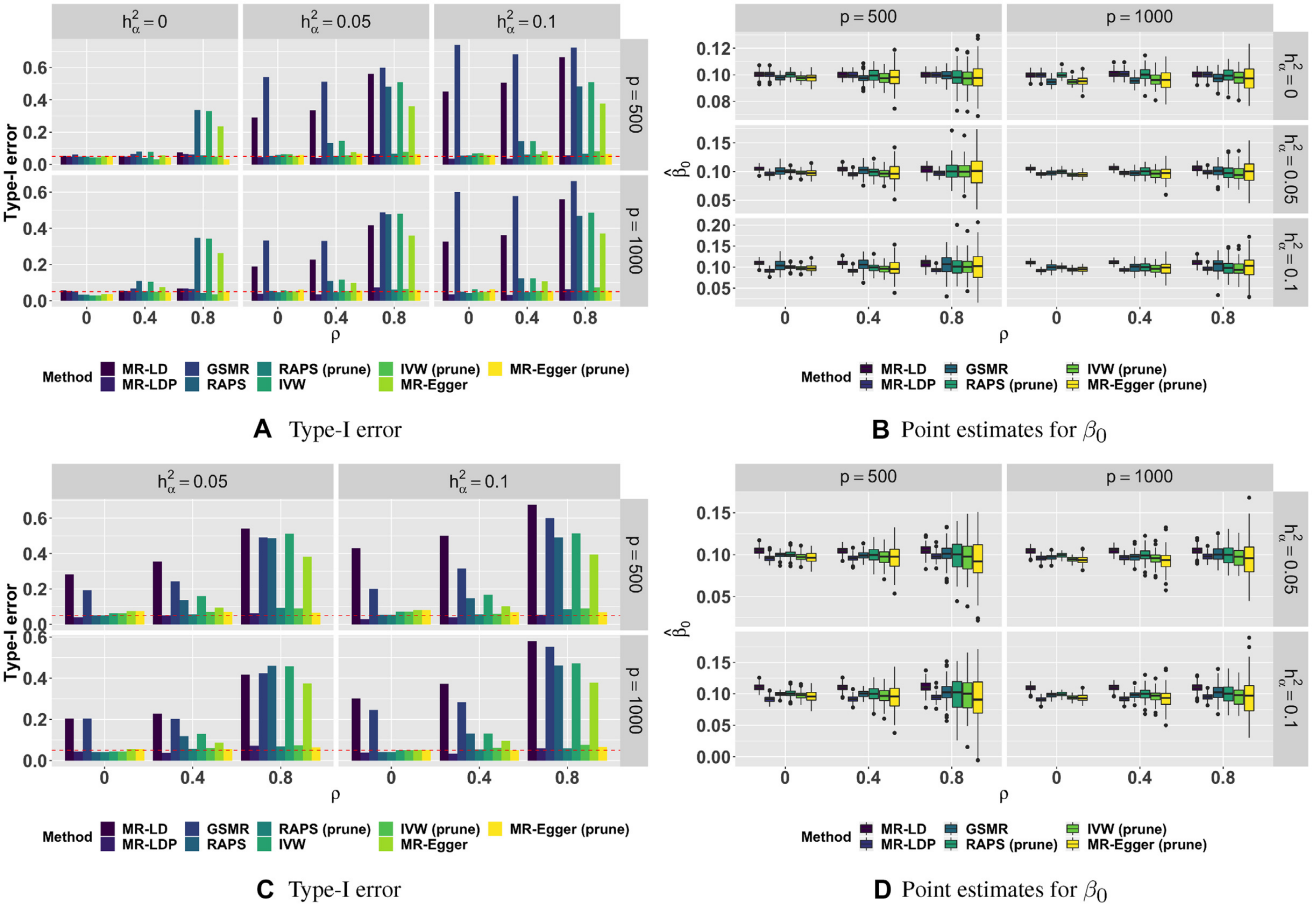
Simulation of type I error control and point estimates under the dense horizontal pleiotropy (**A**, **B**) and the sparse (0.2) horizontal pleiotropy (**C**, **D**). *n*_1_ = *n*_2_ = 20 000; *n*_3_ = 500.

We conducted various simulation studies to make comparisons of MR-LD and MR-LDP with other four commonly used alternative methods: (i) IVW; (ii) MR-Egger; (iii) GSMR; and (iv) RAPS. We first compared the type I error rate for MR-LD and MR-LDP together with other alternative methods based on 1000 replications. The simulation results for dense pleiotropy and sparse pleiotropy with sparsity at 0.2 and 0.4 are shown in Figure 2 and Supplementary Figures S2–S8, respectively, with *n*_3_ = 500, 2500 and 4000, respectively. Note that when $h_{\boldsymbol{\alpha }} ^2=0$, there was no difference between dense and sparse pleiotropy. As shown in the left column of Figure 2A, in the case of no horizontal pleiotropy ($h_{\boldsymbol{\alpha }} ^2=0$), all methods could control type I error at the nominal level of 0.05, generally well when genetic variants were independent (*ρ* = 0). However, as LD becomes stronger (*ρ* = 0.4 or 0.8), the alternative methods failed to control type I error without SNP pruning. In this setting ($h_{\boldsymbol{\alpha }} ^2=0$), MR-LD and MR-LDP performed equally well in type I error control. In the presence of horizontal pleiotropy ($h_{\boldsymbol{\alpha }} ^2=0.05$ or 0.1), as shown in the middle and right columns of Figure 2A, MR-LD failed to control type I error for all *ρ* values, while type I error rates of alternative methods without SNP pruning were not controlled in the case of moderate or strong LD. However, MR-LDP could still control type I error at its nominal level. The similar patterns could be observed for settings under sparse horizontal pleiotropy with sparsity at 0.2 and 0.4 as shown in Figure 2C and Supplementary Figures S4–S8, where the settings was not in favor of MR-LDP. Note that after SNP pruning, genetic variants that remained could be taken as independent. Thus, alternative methods after SNP pruning could control type I error in all settings. However, this is achieved at the expense of losing weak instruments in LD.

Next, we made comparisons of point estimates for MR-LD and MR-LDP together with alternative methods, where SNP pruning was performed for analysis using alternative methods. In this simulation, *β*_0_ = 0.1 and results were based on 100 replications. Clearly, the proposed methods, MR-LD and MR-LDP, had smaller standard errors than alternative methods when LD was moderate or strong (*ρ* = 0.4 or 0.8) (Figure 2B), as SNP pruning causes the alternative methods to use fewer valid instruments. MR-LD and MR-LDP performed equally well in the case of no horizontal pleiotropy, while MR-LD was biased in the presence of horizontal pleiotropy. Similar patterns could be observed for dense and sparse pleiotropy both at sparsity equaling 0.2 and 0.4, as shown in Figure 2D and Supplementary Figures S4–S8.

More simulation settings and their corresponding results can be found in the Supplementary Data, including simulations for robustness and power analysis, simulations using a screening dataset and simulations for binary outcome.

### CAD–CAD and height–height studies

In addition, we used real datasets, i.e. CAD–CAD and height–height pairs, to compare the estimates from MR-LD and MR-LDP with those from the other four alternative methods, where the causal effect *β*_0_ can be taken as known, i.e. *β*_0_ = 1. In these two examples, we used GWAS summary statistics for the same traits (i.e. CAD and BMI, respectively) from three datasets—screening, exposure and outcome (42). The first two datasets are non-overlapping GWAS for the same trait. The exposure dataset and outcome dataset are non-overlapping individuals from European ancestry. Since IVW, MR-Egger and RAPS are designed for independent or weak LD SNPs, and GSMR only works for SNPs with moderate LD, we conducted the LD-based clumping to obtain the near-independent SNPs based on PLINK (43). Individual-level genotype data from UK10K projects served as the reference panel in this study. We used high-performance computing from National SuperComputing Centre, Singapore (https://www.nscc.sg) to accomplish our computational work. For example, if the threshold of 0.1 for *P*-values was applied in the height–height study, there remained 40 521 SNPs and it took ∼2 and ∼8 min, respectively, for MR-LD and MR-LDP to complete the analysis on a Linux platform with a 2.60 GHz Intel Xeon CPU E5-2690 v3 with 30720 KB cache and 96 GB RAM (only 30 GB RAM used). The demo for using *MR.LDP* package can be found both in the Supplementary Data and at GitHub website. In addition, all codes for simulation studies and real data analysis can be found at GitHub website.

**Figure 3.**
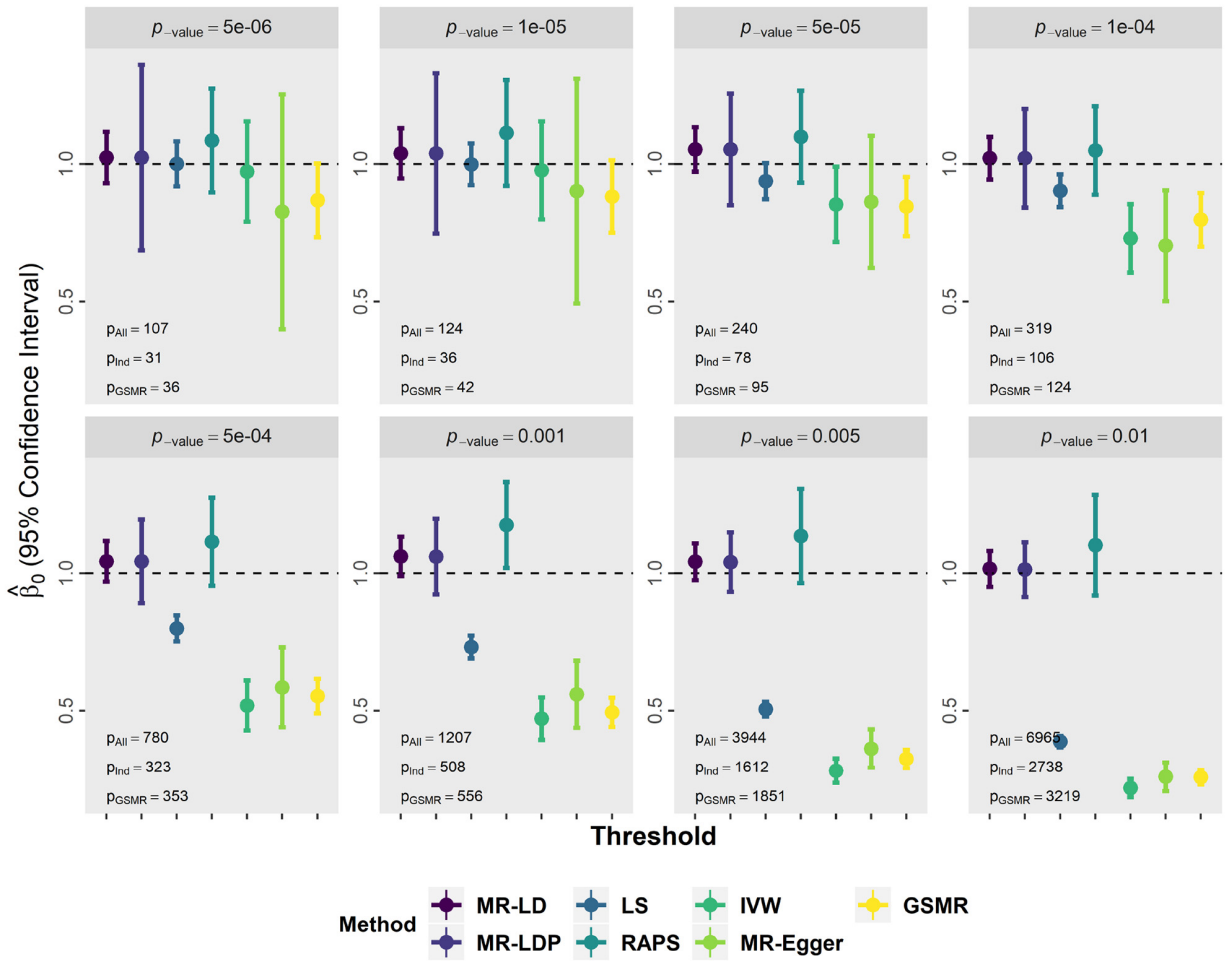
The result of estimates and confidence intervals for CAD–CAD using UK10K as the reference panel with the shrinkage parameter *λ* = 0.1 under different *P*-value thresholds to choose genetic variants in the screening dataset, e.g. *P*-value = 5e−6 and 1e−05. MR-LD, MR-LDP and LS methods use all SNPs selected by the screening dataset (denoted as *P*_All_), but IVW, MR-Egger, RAPS and GSMR use pruned SNPs, where the default value of *r*^2^ is used for GSMR (the number of SNP used: *P*_GSMR_) and *r*^2^ = 0.001 is used for VW, MR-Egger and RAPS (the number of SNP used: *P*_Ind_).

For CAD–CAD analysis, the screening dataset is myocardial infarction (MI) data from UK Biobank (UKB), the exposure data are obtained from the C4D Genetics Consortium (44) and the outcome data are obtained from the transatlantic Coronary ARtery DIsease Genome-wide Replication and Meta-analysis (CARDIoGRAM) (45). We first selected instrumental variants using MI from UKB under different *P*-value thresholds and then conducted MR analysis between the exposure and the outcome using MR-LD, MR-LDP, least squares (LS), IVW, MR-Egger, RAPS and GSMR. First, the scatter plots of $\widehat{\boldsymbol{\gamma }}$ (C4D) against $\widehat{\boldsymbol{\Gamma }}$ (CAD1) are shown in Supplementary Figure S13, where we found that when a larger thresholding value, e.g. *P*-value = 0.001, is applied in the screening dataset (in other words, more genetic variants would be selected for MR analysis), the points crowded in the center make the inference for causality difficult as shown in Supplementary Figure S13. We reported the point estimates with its 95% corresponding confidence intervals for all methods in Figure 3 and Supplementary Figure S14 for *λ* = 0.1 and 0.15, respectively. Clearly, MR-LD and MR-LDP were superior to other methods in terms of smaller bias and shorter confidence intervals when the number of instrumental variants is large. Moreover, the estimates from MR-LD and MR-LDP also exhibited statistical significance consistently, while the coverage of *β*_0_ = 1 from other methods was incorrect under small thresholds except for RAPS with larger standard errors due to the SNP pruning. Additionally, estimates from GSMR, IVW and MR-Egger were always biased when the threshold was small.

Next, we investigated the case that both the exposure and outcome were from human height. In particular, we treated the height in UKB (46) as the screening dataset. The exposure data are from the height for males in a European population-based study, and the outcome data are from the height for females in a European population (47). First, the scatter plot of $\widehat{\boldsymbol{\gamma }}$ (height for males) against $\widehat{\boldsymbol{\Gamma }}$ (height for females) is shown in Supplementary Figure S15. Since height is highly polygenic and the sample size is very large in (47) (around 270 000 individuals), the points are crowded in the middle even with a very small threshold (*P*-value = 5 × 10^−6^). The results of point estimates with their 95% confidence intervals were illustrated in Supplementary Figures S16 and S17 for *λ* = 0.1 and 0.15, respectively. Similar patterns were observed in all cases. In particular, RAPS only offered better performance with larger instrumental variants but did not work for some small thresholds, GSMR failed to estimate the causal effect for this validation study and other methods underestimated the causal effect with relatively large standard errors. MR-LD and MR-LDP used all SNPs passing a certain thresholding value and thus provided more accurate estimates of *β*_0_ = 1.

### The causal effects of lipids and BMI on common human diseases

We further applied our method, MR-LDP, to estimate the causal effects of lipids and BMI on complex diseases, including coronary artery disease (CAD1 and CAD2 from CARDIoGRAM and UKB, respectively), asthma, allergic rhinitis, cancer, major depression disorder, T2D, dyslipidemia (Dyslid), hypertensive disease (Hyper), hemorrhoids, hernia abdominopelvic cavity, insomnia, iron deficiency anemias, irritable bowel syndrome, macular degeneration, osteoarthritis, osteoporosis, PVD, peptic ulcer, psychiatric disorder, acute reaction to stress, VV and disease count (DC). The summary statistics for risk factors include HDL-C, low-density lipoprotein cholesterol (LDL-C), total cholesterol (TC) and BMI. Supplementary Tables S3 and S4 summarize the total number of SNPs and sample sizes for each trait in each health risk factor or disease outcome and the details for the sources of these GWAS summary statistics.

First, we applied MR-LDP together with alternative methods to analyze the exposure–outcome pairs using lipids as the exposure, i.e. HDL-C, LDL-C and TC. Specifically, the screening and exposure datasets were obtained from (48,49), respectively, where the threshold for selecting instrumental variants in the screening dataset is set to 1 × 10^−4^. The association results from the analysis are summarized in Table 1. Note that we did SNP pruning for RAPS, IVW and MR-Egger and used the default settings in all alternative methods. As GSMR removes SNPs by providing an LD threshold, we chose to use *r*^2^ = 0.05, as suggested by its paper (15).

**Table 1.** Causal associations of lipids with common diseases using UK10K as the reference panel with the shrinkage parameter *λ* = 0.1

Lipids	Outcome	*P*_All_	MR-LDP	*P*_GSMR_	GSMR (prune)	*P*_Ind_	RAPS	IVW	MR-Egger
HDL-C	CAD1	2104	**−0.09 (0.027)**	269	**−0.26 (0.038)**	203	**−0.38 (0.07)**	**−0.36 (0.07)**	−0.28 (0.157)
	CAD2	2071	**−0.08 (0.02)**	277	**−0.07 (0.03)**	206	**−0.15 (0.047)**	**−0.15 (0.047)**	−0.08 (0.098)
	T2D	2071	**−0.09 (0.031)**	272	**−0.16 (0.044)**	206	**−0.33 (0.081)**	**−0.35 (0.082)**	0.03 (0.17)
	Dyslid	2071	**−0.14 (0.023)**	255	**−0.1 (0.03)**	206	**−0.23 (0.08)**	**−0.26 (0.076)**	−0.17 (0.158)
	Hyper	2071	**−0.05 (0.017)**	270	**−0.14 (0.022)**	206	**−0.2 (0.037)**	**−0.21 (0.038)**	−0.09 (0.079)
	PVD	2071	**−0.11 (0.048)**	277	−0.12 (0.077)	206	−0.19 (0.109)	−0.19 (0.105)	0.12 (0.222)
	DC	2071	**−0.04 (0.01)**	270	**−0.08 (0.013)**	206	**−0.09 (0.025)**	**−0.1 (0.025)**	−0.03 (0.052)
LDL-C	CAD1	1867	**0.27 (0.029)**	257	**0.42 (0.037)**	193	**0.34 (0.065)**	**0.32 (0.062)**	**0.33 (0.133)**
	CAD2	1820	**0.11 (0.021)**	266	**0.16 (0.027)**	199	**0.15 (0.043)**	**0.14 (0.043)**	**0.26 (0.085)**
	Dyslid	1820	**0.56 (0.03)**	258	**0.94 (0.027)**	199	**0.9 (0.053)**	**0.86 (0.051)**	**0.93 (0.1)**
	DC	1820	**0.08 (0.01)**	267	**0.13 (0.012)**	199	**0.13 (0.019)**	**0.13 (0.019)**	**0.17 (0.037)**
TC	CAD1	2546	**0.24 (0.028)**	309	**0.46 (0.036)**	215	**0.41 (0.061)**	**0.39 (0.062)**	**0.35 (0.146)**
	CAD2	2484	**0.08 (0.02)**	314	**0.16 (0.029)**	218	**0.15 (0.043)**	**0.14 (0.043)**	**0.22 (0.094)**
	Dyslid	2484	**0.54 (0.03)**	303	**1.08 (0.029)**	218	**0.93 (0.055)**	**0.9 (0.051)**	**0.97 (0.111)**
	DC	2484	**0.06 (0.01)**	314	**0.13 (0.012)**	218	**0.12 (0.019)**	**0.12 (0.019)**	**0.14 (0.041)**

MR-LDP uses all SNPs selected by the screening dataset (denoted as *P*_All_), but IVW, MR-Egger, RAPS and GSMR use pruned SNPs, where the default value of *r*^2^ is used for GSMR (the number of SNP used: *P*_GSMR_) and *r*^2^ = 0.001 is used for VW, MR-Egger and RAPS (the number of SNP used: *P*_Ind_). Statistically significant results are indicated in bold.

In practice, HDL-C and LDL-C are often referred to as ‘good’ and ‘bad’ cholesterol, respectively. HDL-C is known to be inversely correlated with heart and vascular diseases. We found several significant protective effects of HDL-C against CAD1 ($\widehat{\beta }= -0.09$), CAD2 ($\widehat{\beta }= -0.08$), T2D ($\widehat{\beta }= -0.09$), Dyslid ($\widehat{\beta }= -0.14$), Hyper ($\widehat{\beta }= -0.05$), PVD ($\widehat{\beta }= -0.11$) and DC ($\widehat{\beta }= -0.04$), which is consistent with known epidemiological associations in the same direction (50–52). Moreover, MR-LDP identified the significant negative causality between HDL-C and PVD, which is consistent with previous studies (53,54). On the other hand, MR-LDP identified the significant positive causality between LDL-C and CAD, which is consistent with the fact that LDL-C narrows the arteries and increases the chance of developing heart diseases. Regarding TC, MR-LDP identified the significant risk effects for cardiovascular disease, as confirmed by RCTs.

**Figure 4.**
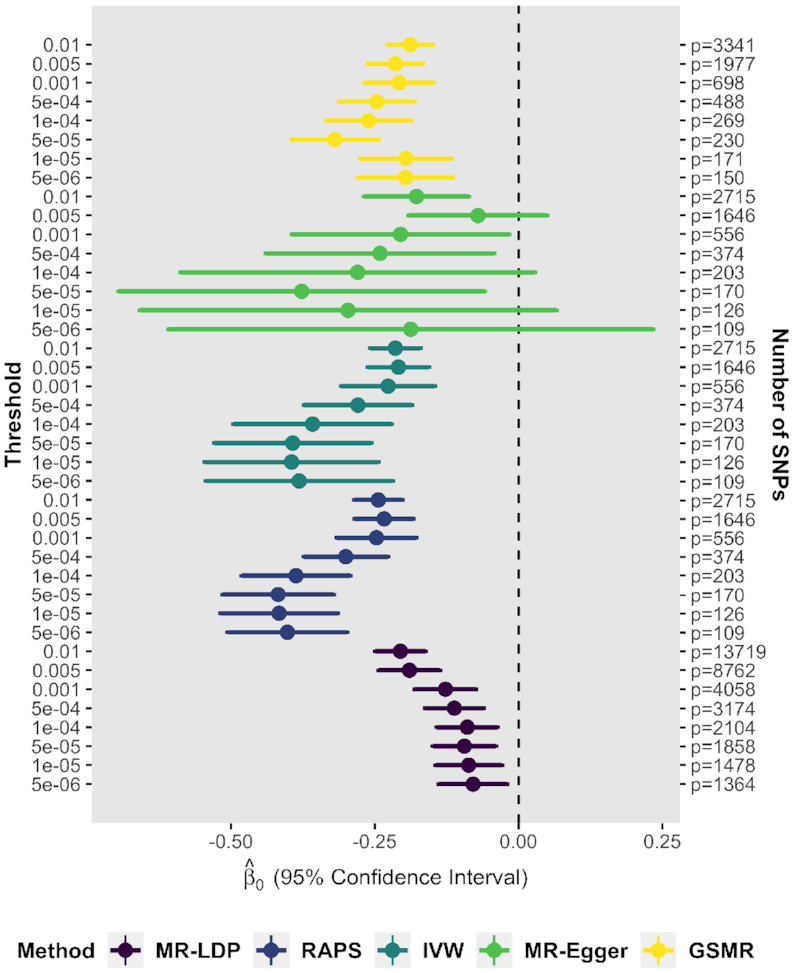
Causal associations of HDL-C on CAD1 under different *P*-value thresholds in the screening dataset, where UK10K was used as the reference panel and the shrinkage parameter *λ* = 0.1.

To better understand the impact of different thresholds, we repeated the analysis for HDL-C on CAD1, CAD2 and PVD, separately, using a sequence of thresholds as shown in Figure 4 and Supplementary Figures S18–S22. Several patterns can be observed: (i) methods taking into account LD have small standard errors; (ii) by using more SNPs under larger thresholds, the standard errors become smaller; and (iii) as thresholds become relatively large, e.g. 0.005, the point estimates tend to be biased. The first two patterns are expected. Generally, MR-LDP is robust under different thresholds but shows biasedness when the threshold is too liberal, which is primarily due to the inclusion of invalid variants. As the threshold is relatively large, more genetic variants with no associations to the exposure are included in the analysis, which induces biasedness either upward or downward depending on the directions of effects for invalid instrumental variants.

Second, we examine the associations between BMI and common diseases, where the exposure and the screening datasets were obtained from GIANT (55,56), respectively. We chose the threshold to be 1 × 10^−4^ for selecting the instrumental variants from the screening dataset. The association results from the analysis are summarized in Table 2. Overall, our MR-LDP detected a relatively more significant causality between BMI and complex diseases in this study. Our findings are consistent with RCTs, which indicate that obesity increases the risk of diseases such as heart disease, T2D and hypertensive disease (57).

**Table 2.** Causal associations of BMI with common diseases using UK10K as the reference panel with the shrinkage parameter *λ* = 0.1

Outcome	*P*_All_	MR-LDP	*P*_GSMR_	GSMR (prune)	*P*_Ind_	RAPS	IVW	MR-Egger
CAD1	4405	**0.2 (0.084)**	701	**0.33 (0.07)**	563	0.2 (0.121)	0.17 (0.091)	0.2 (0.129)
Asthma	4428	**0.28 (0.073)**	707	**0.23 (0.061)**	563	**0.24 (0.107)**	**0.19 (0.08)**	0.18 (0.115)
CAD2	4428	**0.23 (0.066)**	708	**0.21 (0.062)**	563	**0.26 (0.105)**	**0.2 (0.079)**	0.22 (0.113)
T2D	4428	**0.85 (0.141)**	708	**0.84 (0.091)**	563	**1.22 (0.16)**	**0.93 (0.124)**	**1.46 (0.175)**
Dyslid	4428	**0.22 (0.076)**	704	**0.29 (0.059)**	563	0.18 (0.133)	0.16 (0.086)	**0.29 (0.124)**
Hemorrhoids	4428	**0.3 (0.135)**	709	0.2 (0.111)	563	0.15 (0.17)	0.11 (0.129)	−0.1 (0.184)
Hyper	4428	**0.47 (0.066)**	703	**0.5 (0.047)**	563	**0.58 (0.095)**	**0.46 (0.067)**	**0.54 (0.097)**
Insomnia	4428	**0.77 (0.235)**	708	**0.85 (0.215)**	563	**1.24 (0.325)**	**0.96 (0.246)**	0.6 (0.353)
Osteoarthritis	4428	**0.27 (0.078)**	709	**0.27 (0.068)**	563	**0.26 (0.114)**	**0.2 (0.084)**	**0.39 (0.119)**
Osteoporosis	4428	**−0.44 (0.178)**	709	**−0.36 (0.15)**	563	**−0.62 (0.238)**	**−0.48 (0.178)**	**−0.73 (0.254)**
PVD	4428	**0.35 (0.167)**	709	**0.41 (0.159)**	563	0.32 (0.242)	0.24 (0.183)	0.41 (0.263)
DC	4428	**0.27 (0.035)**	700	**0.3 (0.027)**	563	**0.3 (0.051)**	**0.23 (0.037)**	**0.26 (0.053)**

MR-LDP uses all SNPs selected by the screening dataset (denoted as *P*_All_), but IVW, MR-Egger, RAPS and GSMR use pruned SNPs, where the default value of *r*^2^ is used for GSMR (the number of SNP used: *P*_GSMR_) and *r*^2^ = 0.001 is used for VW, MR-Egger and RAPS (the number of SNP used: *P*_Ind_). Statistically significant results are indicated in bold.

We also estimated some causal effects that are rarely involved in the previous MR analysis but reported in the epidemiological studies. For instance, BMI is a significant risk factor for hemorrhoids (58).

In addition, MR-Egger is too conservative in identifying the causal relationship between BMI and common diseases, and the same conclusion can be found in (18). Similar to lipid studies, we repeated the analysis for BMI on hemorrhoids and PVD, respectively, using a sequence of thresholds, as shown in Supplementary Figures S23–S26. The patterns are similar to those in Figure 4 and Supplementary Figures S18–S22.

## DISCUSSION

Here, we proposed a statistically rigorous and efficient approach to perform a two-sample MR analysis that accounts for both LD structure and horizontal pleiotropy using GWAS summary statistics and a genotype reference panel. We implemented our method in the R package *MR.LDP*, which is available for download at GitHub. MR-LDP jointly estimates the causal effect through an approximated likelihood of two sets of GWAS summary statistics for both the risk factor and disease outcome using an additional variance component to eliminate the impact of horizontal pleiotropy. Thus, the type I error can be well controlled even if horizontal pleiotropy and LD structure exist among instrumental variants. MR-LDP is indeed statistically more powerful than other methods in identifying causal effects as illustrated in our simulations (see the ‘Simulations’ section).

Unlike other MR methods, MR-LDP is particularly suitable to analyze complex traits that have multiple instrumental variants within LD. This is primarily accomplished by jointly modeling the distributions for summary statistics and the causal relationship between the risk factor and disease outcome. These summary statistics distributions rely on the polygenicity in complex traits. Moreover, we model the causality by Equation (4) as the average of ‘local’ causal effect, which can be treated similarly as linear structural model in the Supplementary Data. Similar to RAPS, MR-LDP further eliminates the impact of horizontal pleiotropy using a random component. Consequently, MR-LDP is invariant to the orientation of genetic variants, while the results from MR-Egger depend on this orientation as MR-Egger uses a fixed intercept. We notice that a Gaussian distribution with a mean of zero is generally robust even in the case that the underlying horizontal pleiotropy is sparse. In the framework of EM algorithm, the complete-data likelihood for MR-LDP can be written as Equation (5). To further speed up the computation, we developed a PX-VBEM algorithm by expanding parameters and using VB. We further accelerated MR-LDP by parallel computing implemented in the package *MR.LDP*. To further conduct hypothesis testing for causal effects, we calibrated the EBLO from the PX-VBEM algorithm. In our numerical studies, we observe that only GSMR can handle genetic variants with weak LD but is not applicable to analyze genetic variants with high LD. We further demonstrate that unlike other methods, MR-LDP is more effective in controlling type I error in the presence of LD and either sparse or dense horizontal pleiotropy. These merits enable us to apply MR-LDP on GWAS summary statistics, likely discovering more fruitful and meaningful causal relationships in the future.

We used two pairs (CAD–CAD and height–height) of real data to validate the proposed method partially. As the risk factor and the outcome are the same, we can take the true causal effect as known (*β*_0_ = 1). By applying MR-LD and MR-LDP with alternative methods, we found that estimates from the proposed methods can effectively cover the true *β*_0_ at the 95% confidence level, for instrument variants chosen under a wide range of thresholds. When more instrumental variants come into the model under a less stringent threshold, the estimates for the causality have narrower confidence intervals or smaller standard errors. We also note that MR-LDP has a wider confidence interval than MR-LD. This wider confidence interval is because MR-LDP makes additional efforts to model the horizontal pleiotropy.

In this article, we primarily focus on modeling the lipids and BMI as the exposures and complex diseases as the outcomes. Using a threshold of 1 × 10^−4^ in the screening dataset, we identified multiple pairs of significant causal relationships. For example, the well-known protective effect of HDL-C on the PVD (59) was identified by our model. We also identified significant causal relationships between HDL-C and CAD in two parallel experiments. In particular, although HDL-C was found to be associated with CAD in multiple observational studies (60–62), the role of HDL-C in CAD was overturned by later studies (63,64). Recently, Zhao *et al.* (42) showed that the effect of HDL-C in CAD is heterogeneous using different instruments. For BMI, we identified its positive association with knee osteoarthritis and sleep duration, which is consistent with what have been reported in (65,66), respectively. We also confirmed a protective effect of BMI on osteoporosis as suggested previously by (67,68). Moreover, increased BMI is also considered to be one of the contributing factors for PVD in both our study and other related work (69). We further demonstrated the robustness of MR-LDP using a sequence of threshold values to select instrumental variants. As illustrated in Figure 4, the point estimates for *P*-value threshold ≤10^−4^ are almost stable; hence, we want to suggest 10^−4^ as the starting *P*-value cutoff for users to work with.

Even though MR-LDP accounts two important issues, it has several limitations. First, MR-LDP cannot be utilized if there are overlapping samples between SNP exposure and SNP outcome. Nowadays, GWAS consortia usually generate summary statistics using the meta-analysis from many completed studies, which makes samples inevitably overlapped. Moreover, different analyses involving UKB (70) samples are largely overlapped with each other. Second, similar to RAPS and other methods, MR-LDP requires an additional independent SNP exposure dataset to select instrumental variants, otherwise existing selection bias would invalidate the inference. Third, similar to many existing methods including RAPS, MR-LDP relies on the ‘Instrument Strength Independent of Direct Effect’ assumption to account for horizontal pleiotropy. Therefore, methods capable of accounting for correlated pleiotropy are highly anticipated.

## WEB RESOURCES

*MR.LDP* is available at GitHub (https://github.com/QingCheng0218/MR.LDP).

BMI-JAP (screen) dataset: ftp://ftp.ebi.ac.uk/pub/databases/gwas/summary_statistics/AkiyamaM_28892062_GCST004904.

Height and BMI (exposure) datasets: https://portals.broadinstitute.org/collaboration/giant/index.php/GIANT_consortium_data_files#2018_GIANT_and_UK_ BioBank_Meta_Analysis_for_Public_Release

Lipid (screen) datasets: http://csg.sph.umich.edu/willer/public/lipids2010/.

Lipid (exposure) datasets: http://csg.sph.umich.edu/willer/public/lipids2013/.

CAD1 dataset: http://www.cardiogramplusc4d.org/data-downloads/

Common human disease datasets: http://cnsgenomics.com/data.html.

UK10K datasets: https://www.uk10k.org/data_access.html.

## Supplementary Material

lqaa028_Supplemental_File
