# Supplementary material for: MR-LDP: a two-sample Mendelian randomization for GWAS summary statistics accounting for linkage disequilibrium and horizontal pleiotropy
Source: NAR Genom Bioinform. 2020 May 4;2(2):lqaa028. doi: 10.1093/nargab/lqaa028 (PMC7671398; doi:10.1093/nargab/lqaa028)
Supplement: lqaa028_Supplemental_File [file lqaa028_supplemental_file.pdf]

The Supplementary of "MR-LDP: a two-sample Mendelian randomization for GWAS summary statistics accounting for linkage disequilibrium and horizontal pleiotropy"

Qing Cheng[1], Yi Yang[1], Xingjie Shi[1,2], Kar-Fu Yeung[1], Can Yang[3], Heng Peng[4], Jin Liu[1*]

[1]Centre for Quantitative Medicine, Health Services & Systems Research, Duke-NUS Medical School

[2]Department of Statistics, Nanjing University of Finance and Economics, Nanjing, China

[3]Department of Mathematics, The Hong Kong University of Science and Technology

[4]Department of Mathematics, Hong Kong Baptist University

*To whom the correspondence should be addressed

*Correspondence should be addressed to Jin Liu (jin.liu@duke-nus.edu.sg)

Contents

1 Statistical Model for MR-LD and MR-LDP **4**

1.1 Validity of instrumental variables 4

1.2 Model for MR-LD 5

2 The derivation of PX-VBEM algorithm **7**

2.1 PX-VBEM for MR-LD 7

2.1.1 Variational E-step: 7

2.1.2 Variational M-step: 8

2.1.3 Statistical Inference for MR-LD 10

2.2 PX-VBEM for MR-LDP 11

2.2.1 Variational E-step: 11

2.2.2 Variational M-step: 12

2.2.3 Statistical Inference for MR-LDP 14

3 More simulation results for different settings **16**

4 Real Data Analysis **30**

4.1 Two validation studies 30

4.1.1 CAD-CAD study 30

4.1.2 Height-Height study 32

4.2 Applications to the effect of lipids and BMI on common diseases 35

4.2.1 Lipids-disease outcome 35

4.2.2 BMI-disease outcome 41

5 The detail information of GWAS Datasets **46**

6 The vignette of R package: *MR.LDP* **48**

6.1 Introduction 48

6.2 Fit MR-LDP using simulated data 48

6.3 Fit MR-LDP using CAD-CAD study. 51

1 Statistical Model for MR-LD and MR-LDP

1.1 Validity of instrumental variables

Denote $\mathbf{G} \in \mathbb{R}^{n\times p}$ the matrix for p SNPs among n independent individuals, $\mathbf{x} \in \mathbb{R}^{n\times 1}$ and $\mathbf{y} \in \mathbb{R}^{n\times 1}$ the corresponding exposure and outcome variable, respectively, and $\mathbf{U} \in \mathbb{R}^{n\times q}$ the matrix for q confounding factors among n samples. The SNP-exposure and SNP-outcome true effects are denoted as $\boldsymbol{\gamma} \in \mathbb{R}^{p\times 1}$ and $\boldsymbol{\Gamma} \in \mathbb{R}^{p\times 1}$, respectively, for all p SNPs. Then, we can depict the causal model through a directed acyclic graph in Figure S1, where the dashed line represents the horizontal pleiotropy of genetic variants on outcome, denoted as $\boldsymbol{\alpha} \in \mathbb{R}^{p\times 1}$. We start from the classical assumption that all IVs are valid (namely, core assumptions include: all $\boldsymbol{\gamma}$s are non-zero, SNPs are independent of the confounding factors, and no horizontal pleiotropy holds), where the associations of the exposure and the outcome can be framed as the following linear structural model [5]:

$$\mathbf{x} = \sum_{j=1}^{p} \mathbf{g}_j \gamma_j + \mathbf{U}\boldsymbol{\eta}_x + \boldsymbol{\epsilon}_x, \quad \mathbf{y} = \beta_0 \mathbf{x} + \mathbf{U}\boldsymbol{\eta}_y + \boldsymbol{\epsilon}_y, \tag{S1}$$

where β_0 is the effect size of the exposure on the outcome, $\mathbf{g}_j$ is the j-th column of $\mathbf{G}$, $\boldsymbol{\eta}_x$ and $\boldsymbol{\eta}_y$ are effects on confounding factors for exposure and outcome, respectively, and $\boldsymbol{\epsilon}_x$ and $\boldsymbol{\epsilon}_y$ are independent random noises. Importantly, in this model (S1, β_0 can be interpreted as the causal effect of the exposure on the outcome as long as the core assumptions for IV are satisfied [9, 4, 5]. Clearly, the effect size of genetic variant $\mathbf{g}_j$ on the outcome $\mathbf{y}$ can be explicitly expressed as $\Gamma_j = \beta_0 \gamma_j$. Thus Γ_j is linear to γ_j, and β_0 can be interpreted as the causal effect between exposure and outcome in the study [19]. In practice, horizontal pleiotropy is abundant in complex traits and violation of such an assumption can induce severe bias in MR analysis. To investigate the impact of horizontal pleiotropy, one may

consider a modified linear structural model [19]:

$$\mathbf{x} = \sum_{j=1}^{p} \mathbf{g}_j \gamma_j + \mathbf{U}\boldsymbol{\eta}_x + \epsilon_x, \quad \mathbf{y} = \sum_{j=1}^{p} \mathbf{g}_j \alpha_j + \beta_0 \mathbf{x} + \mathbf{U}\boldsymbol{\eta}_y + \epsilon_y, \tag{S2}$$

where genetic variants have direct effects on the outcome, denoted as $\boldsymbol{\alpha} = [\alpha_1, \ldots, \alpha_p]^{\mathrm{T}}$. Implicitly, model (S2) also assumes a linear relationship between γ_j and Γ_j but with a intercept deviating from origin: $\Gamma_j = \alpha_j + \beta_0 \gamma_j$, which is essential to model causality in the presence of horizontal pleiotropy. The linear structural models (S1) and (S2) are not useful in practice as confounding factors are usually not observed in observational studies. However, these two models shed a fundamental insight into modeling causality using summary statistics, namely two-sample MR analysis.

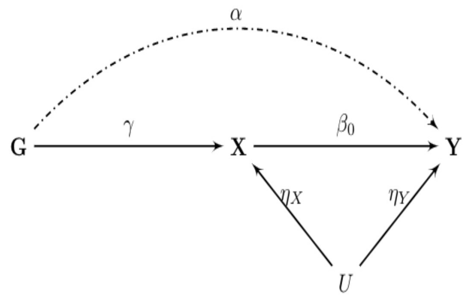

Figure S1: Causal diagram representing three IV assumptions.

1.2 Model for MR-LD

Suppose that all genetic variants satisfy with the core assumptions for an IV model. Invoking by [9, 19], the linear structural model (S1) suggests that we can model the causal effect in

the absence of pleiotropy as

$$\Gamma_k = \beta_0 \gamma_k, \quad \text{for } k = 1, \cdots, p, \tag{S3}$$

where β_0 is the causal effect of interest. We assign a Gaussian prior on each γ_k, that is $\boldsymbol{\gamma} \sim \mathcal{N}(\mathbf{0}, \sigma_{\boldsymbol{\gamma}}^{\mathbf{2}} \mathbf{I_p})$. The Gaussian prior is widely used in genetics studies due to polygenicity [16, 20, 21], which offers a great computational advantage over the complicated ones. Combining Equations (3) and (S3), the likelihood for summary statistics from SNP-outcome can be writen as

$$\widehat{\boldsymbol{\Gamma}} | \boldsymbol{\Gamma}, \widehat{\mathbf{R}}, \widehat{\mathbf{S}}_\Gamma \quad \sim \quad \mathcal{N}(\widehat{\mathbf{S}}_\Gamma \widehat{\mathbf{R}} \widehat{\mathbf{S}}_\Gamma^{-1} \beta_0 \boldsymbol{\gamma}, \widehat{\mathbf{S}}_\Gamma \widehat{\mathbf{R}} \widehat{\mathbf{S}}_\Gamma). \tag{S4}$$

Taking $\boldsymbol{\gamma}$ as the latent variables, the completely-data likelihood can be written as follows

$$\Pr(\widehat{\boldsymbol{\Gamma}}, \widehat{\boldsymbol{\gamma}}, \boldsymbol{\gamma} | \widehat{\mathbf{S}}_{\boldsymbol{\gamma}}, \widehat{\mathbf{S}}_{\boldsymbol{\Gamma}}, \widehat{\mathbf{R}}; \boldsymbol{\theta}) = \mathcal{N}(\widehat{\mathbf{S}}_{\boldsymbol{\Gamma}} \widehat{\mathbf{R}} \widehat{\mathbf{S}}_{\boldsymbol{\Gamma}}^{-1} \beta_0 \boldsymbol{\gamma}, \widehat{\mathbf{S}}_{\boldsymbol{\Gamma}} \widehat{\mathbf{R}} \widehat{\mathbf{S}}_{\boldsymbol{\Gamma}}) \, \mathcal{N}(\widehat{\mathbf{S}}_{\boldsymbol{\gamma}} \widehat{\mathbf{R}} \widehat{\mathbf{S}}_{\boldsymbol{\gamma}}^{-1} \boldsymbol{\gamma}, \widehat{\mathbf{S}}_{\boldsymbol{\gamma}} \widehat{\mathbf{R}} \widehat{\mathbf{S}}_{\boldsymbol{\gamma}}) \, \mathcal{N}(\mathbf{0}, \sigma_{\boldsymbol{\gamma}}^{\mathbf{2}} \mathbf{I_p}), \tag{S5}$$

where $\boldsymbol{\theta} = \{\beta_0, \sigma_{\boldsymbol{\gamma}}^2\}$ is the collection of model parameters. The marginal likelihood can be obtained through integrating out the latent variable $\boldsymbol{\gamma}$, that is

$$\Pr(\widehat{\boldsymbol{\Gamma}}, \widehat{\boldsymbol{\gamma}} | \widehat{\mathbf{S}}_{\boldsymbol{\gamma}}, \widehat{\mathbf{S}}_{\boldsymbol{\Gamma}}, \widehat{\mathbf{R}}; \boldsymbol{\theta}) = \int_{\boldsymbol{\gamma}} \Pr(\widehat{\boldsymbol{\Gamma}}, \widehat{\boldsymbol{\gamma}}, \boldsymbol{\gamma} | \widehat{\mathbf{S}}_{\boldsymbol{\gamma}}, \widehat{\mathbf{S}}_{\boldsymbol{\Gamma}}, \widehat{\mathbf{R}}; \boldsymbol{\theta}) d\boldsymbol{\gamma}.$$

Note that we further expand MR-LD (S5) as the following equation.

$$\Pr(\widehat{\boldsymbol{\Gamma}}, \widehat{\boldsymbol{\gamma}}, \boldsymbol{\gamma} | \widehat{\mathbf{S}}_{\boldsymbol{\gamma}}, \widehat{\mathbf{S}}_{\boldsymbol{\Gamma}}, \widehat{\mathbf{R}}; \boldsymbol{\theta}) = \mathcal{N}(\widehat{\mathbf{S}}_{\boldsymbol{\Gamma}} \widehat{\mathbf{R}} \widehat{\mathbf{S}}_{\boldsymbol{\Gamma}}^{-1} \beta_0 \boldsymbol{\gamma}, \widehat{\mathbf{S}}_{\boldsymbol{\Gamma}} \widehat{\mathbf{R}} \widehat{\mathbf{S}}_{\boldsymbol{\Gamma}}) \, \mathcal{N}(\xi \widehat{\mathbf{S}}_{\boldsymbol{\gamma}} \widehat{\mathbf{R}} \widehat{\mathbf{S}}_{\boldsymbol{\gamma}}^{-1} \boldsymbol{\gamma}, \widehat{\mathbf{S}}_{\boldsymbol{\gamma}} \widehat{\mathbf{R}} \widehat{\mathbf{S}}_{\boldsymbol{\gamma}}) \, \mathcal{N}(\mathbf{0}, \sigma_{\boldsymbol{\gamma}}^{\mathbf{2}} \mathbf{I_p}),$$

where $\boldsymbol{\theta} \stackrel{\text{def}}{=} \{\beta_0, \sigma_{\boldsymbol{\gamma}}, \xi\}$ is the collection of model parameters.

2 The derivation of PX-VBEM algorithm

In this section, we present the details on deriving PX-VBEM algorithm for both MR-LD and MR-LDP.

2.1 PX-VBEM for MR-LD

2.1.1 Variational E-step:

Let $\boldsymbol{\theta} \stackrel{\text{def}}{=} \{\beta_0, \sigma_\gamma, \xi\}$ be the collection of model parameters, the log of the complete-data likelihood can be written as

$$
\begin{aligned}
& \log \Pr(\widehat{\boldsymbol{\Gamma}}, \widehat{\boldsymbol{\gamma}}, \boldsymbol{\gamma} | \widehat{\mathbf{S}}_\gamma, \widehat{\mathbf{S}}_{\boldsymbol{\Gamma}}, \widehat{\mathbf{R}}; \boldsymbol{\theta}) \\
= & \log\ \mathcal{N}(\widehat{\mathbf{S}}_{\boldsymbol{\Gamma}} \widehat{\mathbf{R}} \widehat{\mathbf{S}}_{\boldsymbol{\Gamma}}^{-1} \beta_0 \boldsymbol{\gamma}, \widehat{\mathbf{S}}_{\boldsymbol{\Gamma}} \widehat{\mathbf{R}} \widehat{\mathbf{S}}_{\boldsymbol{\Gamma}}) N(\xi \widehat{\mathbf{S}}_\gamma \widehat{\mathbf{R}} \widehat{\mathbf{S}}_\gamma^{-1} \boldsymbol{\gamma}, \widehat{\mathbf{S}}_\gamma \widehat{\mathbf{R}} \widehat{\mathbf{S}}_\gamma)\ \mathcal{N}(0, \sigma_\gamma^2 \mathbf{I}_p) \\
= & \frac{p}{2}\log(2\pi) - \frac{1}{2}\log|\widehat{\mathbf{S}}_{\boldsymbol{\Gamma}} \widehat{\mathbf{R}} \widehat{\mathbf{S}}_{\boldsymbol{\Gamma}}| - \frac{1}{2}(\widehat{\boldsymbol{\Gamma}} - \widehat{\mathbf{S}}_{\boldsymbol{\Gamma}} \widehat{\mathbf{R}} \widehat{\mathbf{S}}_{\boldsymbol{\Gamma}}^{-1} \beta_0 \boldsymbol{\gamma})^{\mathrm{T}} (\widehat{\mathbf{S}}_{\boldsymbol{\Gamma}} \widehat{\mathbf{R}} \widehat{\mathbf{S}}_{\boldsymbol{\Gamma}})^{-1} (\widehat{\boldsymbol{\Gamma}} - \widehat{\mathbf{S}}_{\boldsymbol{\Gamma}} \widehat{\mathbf{R}} \widehat{\mathbf{S}}_{\boldsymbol{\Gamma}}^{-1} \beta_0 \boldsymbol{\gamma}) \\
- & \frac{p}{2}\log(2\pi) - \frac{1}{2}\log|\widehat{\mathbf{S}}_\gamma \widehat{\mathbf{R}} \widehat{\mathbf{S}}_\gamma| - \frac{1}{2}(\widehat{\boldsymbol{\gamma}} - \xi \widehat{\mathbf{S}}_\gamma \widehat{\mathbf{R}} \widehat{\mathbf{S}}_\gamma^{-1} \boldsymbol{\gamma})^{\mathrm{T}} (\widehat{\mathbf{S}}_\gamma \widehat{\mathbf{R}} \widehat{\mathbf{S}}_\gamma)^{-1} (\widehat{\boldsymbol{\gamma}} - \xi \widehat{\mathbf{S}}_\gamma \widehat{\mathbf{R}} \widehat{\mathbf{S}}_\gamma^{-1} \boldsymbol{\gamma}) \\
- & \frac{p}{2}\log(2\pi) - \frac{p}{2}\log\sigma_\gamma^2 - \frac{1}{2\sigma_\gamma^2}\sum_{k=1}^{p}\gamma_k^2 \qquad \text{(S1)}
\end{aligned}
$$

We could obtain the expression of $\log q(\gamma_j)$ as follows with the help of equation (10.9) in [2]

$$
\begin{aligned}
\log q(\gamma_j) = \quad & E_{q_{-j}}[\log \Pr(\widehat{\boldsymbol{\Gamma}}, \widehat{\boldsymbol{\gamma}}, \boldsymbol{\gamma} | \widehat{\mathbf{S}}_\gamma, \widehat{\mathbf{S}}_{\boldsymbol{\Gamma}}, \widehat{\mathbf{R}}; \boldsymbol{\theta})] + const(\gamma_k) \\
= \quad & E_{q_{-j}}\left[\left(\frac{\xi}{\widehat{\gamma}_j}\widehat{\mathbf{s}}_{\gamma j}^2 - \frac{\xi^2}{\widehat{\mathbf{s}}_{\gamma j}}\sum_{k\neq j}\frac{\gamma_k \widehat{\mathbf{R}}_{jk}}{\widehat{\mathbf{s}}_{\gamma k}} + \frac{\beta_0 \widehat{\Gamma}_j}{\widehat{\mathbf{s}}_{\boldsymbol{\Gamma} j}^2} - \frac{\beta_0^2}{\widehat{\mathbf{s}}_{\boldsymbol{\Gamma} j}}\sum_{k\neq j}\frac{\widehat{\mathbf{R}}_{jk}\gamma_k}{\widehat{\mathbf{s}}_{\boldsymbol{\Gamma} k}}\right)\gamma_j\right. \\
- \quad & \left.\frac{1}{2}\left(\frac{\xi^2 \widehat{\mathbf{R}}_{jj}}{\widehat{\mathbf{s}}_{\gamma j}^2} + \frac{\beta_0^2 \widehat{\mathbf{R}}_{jj}}{\widehat{\mathbf{s}}_{\boldsymbol{\Gamma} j}^2} + \frac{1}{\sigma_\gamma^2}\right)\gamma_j^2\right] + const(\gamma_k),
\end{aligned}
$$

where the notation $E_{q_{-j}}[\cdot]$ denotes an expectation with respect to the q distributions over all variables γ_i , for $i \neq j$. Hence the posterior distribution $\log(\gamma_j)$ should be Gaussian $\mathcal{N}(\mu_j, \sigma_j^2)$

with parameters

$$\begin{aligned}
\frac{1}{\sigma_j^2} &= \frac{\xi^2 \widehat{\mathbf{R}}_{jj}}{\widehat{\mathbf{s}}_{\gamma j}^2} + \frac{\beta_0^2 \widehat{\mathbf{R}}_{jj}}{\widehat{\mathbf{s}}_{\Gamma j}^2} + \frac{1}{\sigma_\gamma^2}, \\
\mu_j &= \sigma_j^2 \left(\frac{\xi \widehat{\gamma}_j}{\widehat{\mathbf{s}}_{\gamma j}^2} - \frac{\xi^2}{\widehat{\mathbf{s}}_{\gamma j}} \sum_{k \neq j} \frac{\langle \gamma_k \rangle \widehat{\mathbf{R}}_{jk}}{\widehat{\mathbf{s}}_{\gamma k}} + \frac{\beta_0 \widehat{\Gamma}_j}{\widehat{\mathbf{s}}_{\Gamma j}^2} - \frac{\beta_0^2}{\widehat{\mathbf{s}}_{\Gamma j}} \sum_{k \neq j} \frac{\widehat{\mathbf{R}}_{jk} \langle \gamma_k \rangle}{\widehat{\mathbf{s}}_{\Gamma k}} \right),
\end{aligned} \tag{S2}$$

where $\langle \gamma_k \rangle \overset{\text{def}}{=} E_q(\gamma_k)$.

2.1.2 Variational M-step:

As illustrated before, the ELBO of the marginal log-likelihood can be written as follows given the current estimates of all parameters $\boldsymbol{\theta}$.

$$\mathcal{L}(\boldsymbol{\theta}) = E_{q(\boldsymbol{\gamma})} \left\{ \log \Pr(\widehat{\boldsymbol{\Gamma}}, \widehat{\boldsymbol{\gamma}}, \boldsymbol{\gamma} | \widehat{\mathbf{S}}_\gamma, \widehat{\mathbf{S}}_\Gamma, \widehat{\mathbf{R}}; \boldsymbol{\theta}) \right\} - E_{q(\boldsymbol{\gamma})} \left\{ \log q(\boldsymbol{\gamma}) \right\}.$$

The second term is independent with the parameters $\boldsymbol{\theta}$, the first term is the expectation of log-complete-data likelihood evaluated under the variational distribution, which can be written as

$$\begin{aligned}
& E_{q(\boldsymbol{\gamma})} \left\{ \log \Pr(\widehat{\boldsymbol{\Gamma}}, \widehat{\boldsymbol{\gamma}}, \boldsymbol{\gamma} | \widehat{\mathbf{S}}_\gamma, \widehat{\mathbf{S}}_\Gamma, \widehat{\mathbf{R}}; \boldsymbol{\theta}) \right\} \\
= \; & E_{q(\boldsymbol{\gamma})} \left\{ \left(\xi \widehat{\boldsymbol{\gamma}}^{\mathrm{T}} \widehat{\mathbf{S}}_\gamma^{-2} + \beta_0 \widehat{\Gamma}^{\mathrm{T}} \widehat{\mathbf{S}}_\Gamma^{-2} \right) \boldsymbol{\gamma} - \frac{1}{2} \boldsymbol{\gamma}^{\mathrm{T}} \left(\xi^2 \widehat{\mathbf{S}}_\gamma^{-1} \widehat{\mathbf{R}} \widehat{\mathbf{S}}_\gamma^{-1} + \beta_0^2 \widehat{\mathbf{S}}_\Gamma^{-1} \widehat{\mathbf{R}} \widehat{\mathbf{S}}_\Gamma^{-1} + \sigma_\gamma^{-2} \mathbf{I}_p \right) \boldsymbol{\gamma}. \right\} \\
- \; & \frac{p}{2} \log \sigma_\gamma^2 \Big\} + const(\boldsymbol{\theta}) \\
= \; & \left(\xi \widehat{\boldsymbol{\gamma}} \widehat{\mathbf{S}}_\gamma^{-2} + \beta_0 \widehat{\boldsymbol{\Gamma}}^{\mathrm{T}} \widehat{\mathbf{S}}_\Gamma^{-2} \right) \boldsymbol{\mu}_\gamma - \frac{1}{2} \boldsymbol{\mu}_\gamma^{\mathrm{T}} \left(\xi^2 \widehat{\mathbf{S}}_\gamma^{-1} \widehat{\mathbf{R}} \widehat{\mathbf{S}}_\gamma^{-1} + \beta_0^2 \widehat{\mathbf{S}}_\Gamma^{-1} \widehat{\mathbf{R}} \widehat{\mathbf{S}}_\Gamma^{-1} + \sigma_\gamma^{-2} \mathbf{I}_p \right) \boldsymbol{\mu}_\gamma \\
- \; & \frac{1}{2} \xi^2 Tr(\widehat{\mathbf{S}}_\gamma^{-1} \widehat{\mathbf{R}} \widehat{\mathbf{S}}_\gamma^{-1} \boldsymbol{\Sigma}_\gamma) - \frac{1}{2} \beta_0^2 Tr(\widehat{\mathbf{S}}_\Gamma^{-1} \widehat{\mathbf{R}} \widehat{\mathbf{S}}_\Gamma^{-1} \boldsymbol{\Sigma}_\gamma) - \frac{p}{2} \log \sigma_\gamma^2 - \frac{1}{2\sigma_\gamma^2} Tr(\boldsymbol{\Sigma}_\gamma) + const(\boldsymbol{\theta}).
\end{aligned}$$

The second equation is due to the fact: if $\mathbf{x} \sim \mathcal{N}(\mathbf{x} | \boldsymbol{\mu}, \boldsymbol{\Sigma})$, then $E(\mathbf{x}^{\mathrm{T}} \mathbf{A} \mathbf{x}) = \boldsymbol{\mu}^{\mathrm{T}} \mathbf{A} \boldsymbol{\mu} + Tr(\mathbf{A} \boldsymbol{\Sigma})$ for any symmetric matrix $\mathbf{A}$.

By setting the derivative of $E_{q(\boldsymbol{\gamma})} \left\{ \log \Pr(\widehat{\boldsymbol{\Gamma}}, \widehat{\boldsymbol{\gamma}}, \boldsymbol{\gamma} | \widehat{\mathbf{S}}_\gamma, \widehat{\mathbf{S}}_\Gamma, \widehat{\mathbf{R}}; \boldsymbol{\theta}) \right\}$ to zero, we obtain the

new updates for all parameters

$$
\begin{aligned}
\beta_0 &= \left\{\boldsymbol{\mu}_{\boldsymbol{\gamma}}^{\mathrm{T}}\widehat{\mathbf{S}}_{\boldsymbol{\Gamma}}^{-1}\widehat{\mathbf{R}}\widehat{\mathbf{S}}_{\boldsymbol{\Gamma}}^{-1}\boldsymbol{\mu}_{\boldsymbol{\gamma}} + Tr(\widehat{\mathbf{S}}_{\boldsymbol{\Gamma}}^{-1}\widehat{\mathbf{R}}\widehat{\mathbf{S}}_{\boldsymbol{\Gamma}}^{-1}\boldsymbol{\Sigma}_{\boldsymbol{\gamma}})\right\}^{-1}\left(\widehat{\boldsymbol{\Gamma}}^{\mathrm{T}}\widehat{\mathbf{S}}_{\boldsymbol{\Gamma}}^{-2}\boldsymbol{\mu}_{\boldsymbol{\gamma}}\right)\\
\sigma_{\boldsymbol{\gamma}}^2 &= \left\{\boldsymbol{\mu}_{\boldsymbol{\gamma}}^{\mathrm{T}}\boldsymbol{\mu}_{\boldsymbol{\gamma}} + Tr(\boldsymbol{\Sigma}_{\boldsymbol{\gamma}})\right\}/p\\
\xi &= \left\{\boldsymbol{\mu}_{\boldsymbol{\gamma}}^{\mathrm{T}}\widehat{\mathbf{S}}_{\boldsymbol{\gamma}}^{-1}\widehat{\mathbf{R}}\widehat{\mathbf{S}}_{\boldsymbol{\gamma}}^{-1}\boldsymbol{\mu}_{\boldsymbol{\gamma}} + Tr(\widehat{\mathbf{S}}_{\boldsymbol{\gamma}}^{-1}\widehat{\mathbf{R}}\widehat{\mathbf{S}}_{\boldsymbol{\gamma}}^{-1}\boldsymbol{\Sigma}_{\boldsymbol{\gamma}})\right\}^{-1}\left(\widehat{\boldsymbol{\gamma}}^{\mathrm{T}}\widehat{\mathbf{S}}_{\boldsymbol{\gamma}}^{-2}\boldsymbol{\mu}_{\boldsymbol{\gamma}}\right) \qquad \text{(S3)}
\end{aligned}
$$

where $\boldsymbol{\mu}_{\boldsymbol{\gamma}} = (\mu_1, \cdots, \mu_p)^{\mathrm{T}}, \boldsymbol{\Sigma}_{\boldsymbol{\gamma}} = \mathrm{diag}((\sigma_1^2, \cdots, \sigma_p^2)^{\mathrm{T}})$.

Since the entropy of $q(\boldsymbol{\gamma})$ equals $\frac{p}{2}(\log(2\pi)+1) + \frac{1}{2}\sum_{j=1}^{p}\log\sigma_j^2$, the analytic form of ELBO can be obtained as follows

$$
\begin{aligned}
&\mathcal{L}(q)\\
=\;& \xi\widehat{\boldsymbol{\gamma}}\widehat{\mathbf{S}}_{\boldsymbol{\gamma}}^{-2}\boldsymbol{\mu}_{\boldsymbol{\gamma}} - \frac{\xi^2}{2}\boldsymbol{\mu}_{\boldsymbol{\gamma}}^{\mathrm{T}}\widehat{\mathbf{S}}_{\boldsymbol{\gamma}}^{-1}\widehat{\mathbf{R}}\widehat{\mathbf{S}}_{\boldsymbol{\gamma}}^{-1}\boldsymbol{\mu}_{\boldsymbol{\gamma}} - \frac{\xi^2}{2}Tr(\widehat{\mathbf{S}}_{\boldsymbol{\gamma}}^{-1}\widehat{\mathbf{R}}\widehat{\mathbf{S}}_{\boldsymbol{\gamma}}^{-1}\boldsymbol{\Sigma}_{\boldsymbol{\gamma}}) + \beta_0\widehat{\boldsymbol{\Gamma}}^{\mathrm{T}}\widehat{\mathbf{S}}_{\boldsymbol{\Gamma}}^{-2}\boldsymbol{\mu}_{\boldsymbol{\gamma}} - \frac{1}{2}\beta_0^2\boldsymbol{\mu}_{\boldsymbol{\gamma}}^{\mathrm{T}}\widehat{\mathbf{S}}_{\boldsymbol{\Gamma}}^{-1}\widehat{\mathbf{R}}\widehat{\mathbf{S}}_{\boldsymbol{\Gamma}}^{-1}\boldsymbol{\mu}_{\boldsymbol{\gamma}}\\
-\;& \frac{1}{2}\beta_0^2 Tr(\widehat{\mathbf{S}}_{\boldsymbol{\Gamma}}^{-1}\widehat{\mathbf{R}}\widehat{\mathbf{S}}_{\boldsymbol{\Gamma}}^{-1}\boldsymbol{\Sigma}_{\boldsymbol{\gamma}}) - \frac{p}{2}\log\sigma_{\boldsymbol{\gamma}}^2 - \frac{1}{2\sigma_{\boldsymbol{\gamma}}^2}\boldsymbol{\mu}_{\boldsymbol{\gamma}}^{\mathrm{T}}\boldsymbol{\mu}_{\boldsymbol{\gamma}} - \frac{1}{2\sigma_{\boldsymbol{\gamma}}^2}Tr(\boldsymbol{\Sigma}_{\boldsymbol{\gamma}}) + \frac{1}{2}\sum_{j=1}^{p}\log\sigma_j^2 - p\log(2\pi)\\
+\;& \frac{p}{2} - \frac{1}{2}\left\{\log|\widehat{\mathbf{S}}_{\boldsymbol{\gamma}}\widehat{\mathbf{R}}\widehat{\mathbf{S}}_{\boldsymbol{\gamma}}| + \log|\widehat{\mathbf{S}}_{\boldsymbol{\Gamma}}\widehat{\mathbf{R}}\widehat{\mathbf{S}}_{\boldsymbol{\Gamma}}| + \widehat{\boldsymbol{\gamma}}^{\mathrm{T}}(\widehat{\mathbf{S}}_{\boldsymbol{\gamma}}\widehat{\mathbf{R}}\widehat{\mathbf{S}}_{\boldsymbol{\gamma}})^{-1}\widehat{\boldsymbol{\gamma}} + \widehat{\boldsymbol{\Gamma}}^{\mathrm{T}}(\widehat{\mathbf{S}}_{\boldsymbol{\Gamma}}\widehat{\mathbf{R}}\widehat{\mathbf{S}}_{\boldsymbol{\Gamma}})^{-1}\boldsymbol{\Gamma}\right\}.
\end{aligned}
$$

The reduction steps: $\boldsymbol{\mu}_{\boldsymbol{\gamma}} = \xi\boldsymbol{\mu}_{\boldsymbol{\gamma}}$, $\beta_0 = \beta_0/\xi$, $\sigma_{\boldsymbol{\gamma}}^2 = \xi^2\sigma_{\boldsymbol{\gamma}}^2$.

The corresponding PX-VBEM can be summarized as Algorithm 1.

Algorithm 1: PX-VBEM for MR-LD

Initialization: The parameters $(\sigma_{\gamma_j}^2, \sigma_e^2)$ are initialized using linear mixed model. Meanwhile, we set $\beta_0 = 0$, $\mu_{\boldsymbol{\gamma}} = \mathbf{0}$, $\sigma_{\boldsymbol{\gamma}} = 0.01$, $\xi = 1$.

repeat

  E-step: At the t-th iteration, for each $j = 1, \cdots, p$, the posterior distribution $q(\gamma_j|\boldsymbol{\theta}^{(t)})$ follows Gaussian with parameters obtained using formula (S2) given $\xi = \xi^{(t)} = 1$, $\beta_0 = \beta_0^{(t)}$, $\sigma_{\boldsymbol{\gamma}}^2 = (\sigma_{\boldsymbol{\gamma}}^{(t)})^2$.

  M-tesp: Update β_0, $\sigma_{\boldsymbol{\gamma}}^2$ and ξ as equation(S3).

  Reduction-step: Rescale the parameters $\boldsymbol{\mu}_{\boldsymbol{\gamma}}^{(t+1)} = \xi^{(t+1)}\boldsymbol{\mu}_{\boldsymbol{\gamma}}^{(t+1)}$, $\beta_0^{(t+1)} = \beta_0^{(t+1)}/\xi^{(t+1)}$, $(\sigma_j^{(t+1)})^2 = (\xi^{(t+1)})^2(\sigma_j^{(t+1)})^2$ and reset $\xi^{(t+1)} = 1$.

until *convergence or maximum iteration reached*;

2.1.3 Statistical Inference for MR-LD

As VB searches within a factorizable family for posterior distribution, one can only obtain an approximation for the posterior distribution of latent variables. Earlier works showed that VBEM provides useful and accurate posterior mean estimates [3]. Despite its computational efficiency and accuracy for estimating posterior mean, VB suffers from under-estimating the variance of target distribution Thus, the ELBO from VB-type algorithm cannot be used directly as a proxy to log-likelihood. In this paper, we follow Yang et al. [17] and adopt the similar strategy to calibrate ELBO as well as mitigate the bias of variance. We first set up the following hypothesis test to formally examine the significance between the exposure and the outcome:

$$\mathcal{H}_0 : \beta_0 = 0 \qquad \text{v.s.} \qquad \mathcal{H}_1 : \beta_0 \neq 0.$$

A likelihood ratio test (LRT) statistic is given by

$$\Lambda \;=\; 2(\log \Pr(\widehat{\gamma}, \widehat{\mathbf{\Gamma}}|\widehat{\mathbf{S}}_{\gamma}, \widehat{\mathbf{S}}_{\mathbf{\Gamma}}, \widehat{\mathbf{R}}; \widehat{\boldsymbol{\theta}}) - \log \Pr(\widehat{\gamma}, \widehat{\mathbf{\Gamma}}|\widehat{\mathbf{S}}_{\gamma}, \widehat{\mathbf{S}}_{\mathbf{\Gamma}}, \widehat{\mathbf{R}}; \widehat{\boldsymbol{\theta}}_0)), \tag{S4}$$

where $\widehat{\boldsymbol{\theta}}$ and $\widehat{\boldsymbol{\theta}}_0$ are parameters obtained by maximizing the marginal likelihood, under both the null hypothesis and alternative hypothesis, respectively. As the marginal likelihood cannot be obtained but the ELBO with accurate posterior means, we calibrate the ELBO(denoted by $\widetilde{\mathcal{L}}(\boldsymbol{\theta}, \boldsymbol{\mu}_{\boldsymbol{\gamma}})$) as follows

$$\begin{aligned}
\widetilde{\mathcal{L}}(\boldsymbol{\theta}, \boldsymbol{\mu}_{\boldsymbol{\gamma}}) &= E_{q(\boldsymbol{\gamma})}\left\{\log \Pr(\widehat{\mathbf{\Gamma}}, \widehat{\gamma}, \boldsymbol{\gamma}|\widehat{\mathbf{S}}_{\gamma}, \widehat{\mathbf{S}}_{\mathbf{\Gamma}}, \widehat{\mathbf{R}}; \boldsymbol{\theta}) - \log q(\boldsymbol{\gamma})\right\} \\
&= -\frac{1}{2}\boldsymbol{\mu}_{\boldsymbol{\gamma}}^{\mathrm{T}}\left(\xi^2 \widehat{\mathbf{S}}_{\gamma}^{-1}\widehat{\mathbf{R}}\widehat{\mathbf{S}}_{\gamma}^{-1} + \beta_0^2 \widehat{\mathbf{S}}_{\mathbf{\Gamma}}^{-1}\widehat{\mathbf{R}}\widehat{\mathbf{S}}_{\mathbf{\Gamma}}^{-2} + \sigma_{\gamma}^{-2}\mathbf{I}_p\right)\boldsymbol{\mu}_{\boldsymbol{\gamma}} \\
&+ \left(\xi \widehat{\gamma}^{\mathrm{T}}\widehat{\mathbf{S}}_{\gamma}^{-2} + \beta_0 \widehat{\mathbf{\Gamma}}^{\mathrm{T}}\widehat{\mathbf{S}}_{\mathbf{\Gamma}}^{-2}\right)\boldsymbol{\mu}_{\boldsymbol{\gamma}} - \frac{p}{2}\log \sigma_{\gamma}^2 + \frac{1}{2}\log|\widetilde{\mathbf{\Sigma}}_{\boldsymbol{\gamma}}| - p\log(2\pi) \\
&- \frac{1}{2}\left\{\log|\widehat{\mathbf{S}}_{\gamma}\widehat{\mathbf{R}}\widehat{\mathbf{S}}_{\gamma}| + \log|\widehat{\mathbf{S}}_{\mathbf{\Gamma}}\widehat{\mathbf{R}}\widehat{\mathbf{S}}_{\mathbf{\Gamma}}| + \widehat{\gamma}^{\mathrm{T}}(\widehat{\mathbf{S}}_{\gamma}\widehat{\mathbf{R}}\widehat{\mathbf{S}}_{\gamma})^{-1}\widehat{\gamma} + \widehat{\mathbf{\Gamma}}^{\mathrm{T}}(\widehat{\mathbf{S}}_{\mathbf{\Gamma}}\widehat{\mathbf{R}}\widehat{\mathbf{S}}_{\mathbf{\Gamma}})^{-1}\mathbf{\Gamma}\right\},
\end{aligned} \tag{S5}$$

where $\boldsymbol{\mu}_{\boldsymbol{\gamma}}$ and $\widetilde{\mathbf{\Sigma}}_{\boldsymbol{\gamma}}$ are the posterior mean and posterior variance for latent variable $\boldsymbol{\gamma}$ and the form of $\widetilde{\mathbf{\Sigma}}_{\boldsymbol{\gamma}}$ is from EM/PX-EM by plugging the posterior mean estimates and parameter

estimates from VBEM/PX-VBEM which can be expressed as

$$\widetilde{\boldsymbol{\Sigma}}_{\gamma} \;=\; \left(\xi^2\widehat{\mathbf{S}}_{\gamma}^{-1}\widehat{\mathbf{R}}\widehat{\mathbf{S}}_{\gamma}^{-1} + \beta_0^2\widehat{\mathbf{S}}_{\boldsymbol{\Gamma}}^{-1}\widehat{\mathbf{R}}\widehat{\mathbf{S}}_{\boldsymbol{\Gamma}}^{-2} + \sigma_{\gamma}^{-2}\mathbf{I}_p\right)^{-1}.$$

In summary, we recalibrate the ELBO for MR-LD as follows:

1. Obtain parameters together with variational means under both $\mathcal{H}_0$ and $\mathcal{H}_1$.
2. Recalibrate ELBO using formulae (S5).
3. Calculate the test statistic (S4) using the recalibrated ELBO as a proxy to the marginal log-likelihood.

As demonstrated in Yang et al. [17], this procedure can produce the calibrated ELBO that is highly correlated with the marginal log-likelihood obtained from EM algorithm. Moreover, our empirical results in validation studies show that this procedure works well.

2.2 PX-VBEM for MR-LDP

2.2.1 Variational E-step:

The latent variable: $\boldsymbol{\gamma}, \boldsymbol{\alpha}$ and the parameter set $\boldsymbol{\theta} \overset{\text{def}}{=} \{\sigma_\alpha^2, \sigma_\gamma^2, \beta_0, \xi\}$. Therefor, the log-likelihood of complete-data can be written as follows,

$$\begin{aligned}
& \log\Pr(\widehat{\boldsymbol{\Gamma}}, \widehat{\boldsymbol{\gamma}}, \boldsymbol{\alpha}, \boldsymbol{\gamma} | \widehat{\mathbf{S}}_{\gamma}, \widehat{\mathbf{S}}_{\boldsymbol{\Gamma}}, \widehat{\mathbf{R}}; \boldsymbol{\theta}) \\
= \;& \log\, \mathcal{N}(\widehat{\boldsymbol{\Gamma}} | \widehat{\mathbf{S}}_{\boldsymbol{\Gamma}}\widehat{\mathbf{R}}\widehat{\mathbf{S}}_{\boldsymbol{\Gamma}}^{-1}(\beta_0\boldsymbol{\gamma} + \boldsymbol{\alpha}), \widehat{\mathbf{S}}_{\boldsymbol{\Gamma}}\widehat{\mathbf{R}}\widehat{\mathbf{S}}_{\boldsymbol{\Gamma}}) N(\widehat{\boldsymbol{\gamma}} | \xi\widehat{\mathbf{S}}_{\gamma}\widehat{\mathbf{R}}\widehat{\mathbf{S}}_{\gamma}^{-1}\boldsymbol{\gamma}, \widehat{\mathbf{S}}_{\gamma}\widehat{\mathbf{R}}\widehat{\mathbf{S}}_{\gamma})\; \mathcal{N}(\mathbf{0}, \sigma_\alpha^{\mathbf{2}}\mathbf{I_p})\; \mathcal{N}(\mathbf{0}, \sigma_\gamma^{\mathbf{2}}\mathbf{I_p}) \\
= \;& \left(\beta_0\widehat{\boldsymbol{\Gamma}}^{\mathrm{T}}\widehat{\mathbf{S}}_{\boldsymbol{\Gamma}}^{-2} + \xi\widehat{\boldsymbol{\gamma}}\widehat{\mathbf{S}}_{\gamma}^{-2}\right)\boldsymbol{\gamma} - \frac{1}{2}\boldsymbol{\gamma}^{\mathrm{T}}\left(\beta_0^2\widehat{\mathbf{S}}_{\boldsymbol{\Gamma}}^{-1}\widehat{\mathbf{R}}\widehat{\mathbf{S}}_{\boldsymbol{\Gamma}}^{-1} + \xi^2\widehat{\mathbf{S}}_{\gamma}^{-1}\widehat{\mathbf{R}}\widehat{\mathbf{S}}_{\gamma}^{-1} + \sigma_{\gamma}^{-2}\mathbf{I}\right)\boldsymbol{\gamma} + \widehat{\boldsymbol{\Gamma}}\widehat{\mathbf{S}}_{\boldsymbol{\Gamma}}^{-2}\boldsymbol{\alpha} \\
- \;& \beta_0\boldsymbol{\alpha}^{\mathrm{T}}\widehat{\mathbf{S}}_{\boldsymbol{\Gamma}}^{-1}\widehat{\mathbf{R}}\widehat{\mathbf{S}}_{\boldsymbol{\Gamma}}^{-1}\boldsymbol{\gamma} - \frac{p}{2}\log\sigma_{\gamma}^2 - \frac{p}{2}\log\sigma_{\boldsymbol{\alpha}}^2 - \frac{1}{2}\boldsymbol{\alpha}^{\mathrm{T}}\left(\widehat{\mathbf{S}}_{\boldsymbol{\Gamma}}^{-1}\widehat{\mathbf{R}}\widehat{\mathbf{S}}_{\boldsymbol{\Gamma}}^{-1} + \sigma_{\boldsymbol{\alpha}}^{-2}\mathbf{I}\right)\boldsymbol{\alpha} - 2p\log(2\pi) \\
- \;& \frac{1}{2}\left\{\log|\widehat{\mathbf{S}}_{\gamma}\widehat{\mathbf{R}}\widehat{\mathbf{S}}_{\gamma}| + \log|\widehat{\mathbf{S}}_{\boldsymbol{\Gamma}}\widehat{\mathbf{R}}\widehat{\mathbf{S}}_{\boldsymbol{\Gamma}}| + \widehat{\boldsymbol{\gamma}}^{\mathrm{T}}(\widehat{\mathbf{S}}_{\gamma}\widehat{\mathbf{R}}\widehat{\mathbf{S}}_{\gamma})^{-1}\widehat{\boldsymbol{\gamma}} + \widehat{\boldsymbol{\Gamma}}^{\mathrm{T}}(\widehat{\mathbf{S}}_{\boldsymbol{\Gamma}}\widehat{\mathbf{R}}\widehat{\mathbf{S}}_{\boldsymbol{\Gamma}})^{-1}\boldsymbol{\Gamma}\right\}. \qquad \text{(S6)}
\end{aligned}$$

We use mean field theory to approximate the posterior $q(\boldsymbol{\gamma}, \boldsymbol{\alpha}) = q(\gamma_1)\cdots q(\gamma_p)\cdots q(\alpha_1)\cdots q(\alpha_p)$.

The posterior distribution of $\gamma_j \sim \mathcal{N}(\mu_j, \sigma_j^2)$, where

$$\begin{aligned} -\frac{1}{2\sigma_j^2} &= -\frac{\beta_0^2}{2}\frac{\widehat{\mathbf{R}}_{jj}}{\widehat{\mathbf{s}}_{\mathbf{\Gamma}j}^2} - \frac{\xi^2\widehat{\mathbf{R}}_{jj}}{2\widehat{\mathbf{s}}_{\gamma j}^2} - \frac{1}{2\sigma_\gamma^2}, \\ \frac{\mu_j}{\sigma_j^2} &= \beta_0\frac{\widehat{\Gamma}_j}{\widehat{\mathbf{s}}_{\mathbf{\Gamma}j}^2} - \frac{\beta_0^2}{\widehat{\mathbf{s}}_{\mathbf{\Gamma}j}}\left(\sum_{k\neq j}\frac{\langle\gamma_k\rangle\widehat{\mathbf{R}}_{jk}}{\widehat{\mathbf{s}}_{\mathbf{\Gamma}k}}\right) - \frac{\beta_0}{\widehat{\mathbf{s}}_{\mathbf{\Gamma}j}}\left(\sum_{i=1}^{p}\frac{\alpha_i\widehat{\mathbf{R}}_{ij}}{\widehat{\mathbf{s}}_{\mathbf{\Gamma}i}}\right) + \frac{\xi\widehat{\gamma}_j}{\widehat{\mathbf{s}}_{\gamma j}^2} - \frac{\xi^2}{\widehat{\mathbf{s}}_{\gamma j}}\left(\sum_{k\neq j}\frac{\langle\gamma_k\rangle\widehat{\mathbf{R}}_{jk}}{\widehat{\mathbf{s}}_{\gamma k}}\right). \end{aligned} \tag{S7}$$

where $\langle\gamma_k\rangle \overset{\text{def}}{=} E_q(\gamma_k)$.

Therefore, we can denote the posterior of $\boldsymbol{\gamma}$ by $\mathcal{N}(\boldsymbol{\gamma}|\mu_{\boldsymbol{\gamma}}, \mathbf{\Sigma}_{\boldsymbol{\gamma}})$, where $\mu_{\boldsymbol{\gamma}} = (\mu_1, \cdots, \mu_p)^{\mathrm{T}}$ and $\mathbf{\Sigma}_{\boldsymbol{\gamma}} = \mathrm{diag}((\sigma_1, \cdots, \sigma_p))$

The posterior distribution of $\alpha_k \sim \mathcal{N}(\widetilde{\mu}_k, \widetilde{\sigma}_k^2)$, where

$$\begin{aligned} -\frac{1}{2\widetilde{\sigma}_k^2} &= -\frac{1}{2}\frac{\widehat{\mathbf{R}}_{kk}}{\widehat{\mathbf{s}}_{\mathbf{\Gamma}k}^2} - \frac{1}{2\sigma_\alpha^2}, \\ \frac{\widetilde{\mu}_k}{\widetilde{\sigma}_k^2} &= \frac{\widehat{\Gamma}_k}{\widehat{\mathbf{s}}_{\mathbf{\Gamma}k}^2} - \frac{\beta_0}{\widehat{\mathbf{s}}_{\mathbf{\Gamma}k}}\sum_{i=1}^{p}\frac{\widehat{\mathbf{R}}_{ik}\langle\gamma_i\rangle}{\widehat{\mathbf{s}}_{\mathbf{\Gamma}`i}} - \frac{1}{\widehat{\mathbf{s}}_{\mathbf{\Gamma}k}}\sum_{j\neq k}\frac{\langle\alpha_j\rangle\widehat{\mathbf{R}}_{jk}}{\widehat{\mathbf{s}}_{\mathbf{\Gamma}j}}, \end{aligned} \tag{S8}$$

where, $\langle\alpha_j\rangle \overset{\text{def}}{=} E_q(\alpha_j)$.

Similarly, $\boldsymbol{\alpha} \sim \mathcal{N}(\boldsymbol{\alpha}|\mu_{\boldsymbol{\alpha}}, \mathbf{\Sigma}_{\boldsymbol{\alpha}})$, where $\mu_{\boldsymbol{\alpha}} = (\widetilde{\mu}_1, \cdots, \widetilde{\mu}_p)^{\mathrm{T}}$ and $\mathbf{\Sigma}_{\boldsymbol{\alpha}} = \mathrm{diag}((\widetilde{\sigma}_1, \cdots, \widetilde{\sigma}_p))$

2.2.2 Variational M-step:

Given the current estimates of all parameters $\boldsymbol{\theta}$, the ELBO of the marginal log-likelihood can be written as follows

$$\mathcal{L}(\boldsymbol{\theta}) = E_{q(\boldsymbol{\gamma},\boldsymbol{\alpha})}\left\{\log\Pr(\widehat{\mathbf{\Gamma}}, \widehat{\boldsymbol{\gamma}}, \boldsymbol{\alpha}, \boldsymbol{\gamma}|\widehat{\mathbf{S}}_{\boldsymbol{\gamma}}, \widehat{\mathbf{S}}_{\mathbf{\Gamma}}, \widehat{\mathbf{R}}; \boldsymbol{\theta})\right\} - E_{q(\boldsymbol{\gamma})}\left\{\log q(\boldsymbol{\gamma})\right\} - E_{q(\boldsymbol{\alpha})}\left\{\log q(\boldsymbol{\alpha})\right\}.$$

Only the first term contains the parameters $\boldsymbol{\theta}$, which is the expectation of log-complete-data likelihood, in details

$$\begin{aligned} &E_{q(\boldsymbol{\gamma},\boldsymbol{\alpha})}\left\{\log\Pr(\widehat{\mathbf{\Gamma}}, \widehat{\boldsymbol{\gamma}}, \boldsymbol{\alpha}, \boldsymbol{\gamma}|\widehat{\mathbf{S}}_{\boldsymbol{\gamma}}, \widehat{\mathbf{S}}_{\mathbf{\Gamma}}, \widehat{\mathbf{R}}; \boldsymbol{\theta})\right\} \\ = &-\frac{1}{2}\beta_0^2\left\{\boldsymbol{\mu}_{\boldsymbol{\gamma}}^{\mathrm{T}}\widehat{\mathbf{S}}_{\mathbf{\Gamma}}^{-1}\widehat{\mathbf{R}}\widehat{\mathbf{S}}_{\mathbf{\Gamma}}^{-1}\boldsymbol{\mu}_{\boldsymbol{\gamma}} + Tr(\widehat{\mathbf{S}}_{\mathbf{\Gamma}}^{-1}\widehat{\mathbf{R}}\widehat{\mathbf{S}}_{\mathbf{\Gamma}}^{-1}\mathbf{\Sigma}_{\boldsymbol{\gamma}})\right\} - \frac{1}{2}\left\{\boldsymbol{\mu}_{\boldsymbol{\alpha}}^{\mathrm{T}}\widehat{\mathbf{S}}_{\mathbf{\Gamma}}^{-1}\widehat{\mathbf{R}}\widehat{\mathbf{S}}_{\mathbf{\Gamma}}^{-1}\boldsymbol{\mu}_{\boldsymbol{\alpha}} + Tr(\widehat{\mathbf{S}}_{\mathbf{\Gamma}}^{-1}\widehat{\mathbf{R}}\widehat{\mathbf{S}}_{\mathbf{\Gamma}}^{-1}\mathbf{\Sigma}_{\boldsymbol{\alpha}})\right\} \end{aligned}$$

$$
\begin{aligned}
&- \frac{1}{2}\left\{\xi^2\boldsymbol{\mu}_{\boldsymbol{\gamma}}^{\mathrm{T}}\widehat{\mathbf{S}}_{\boldsymbol{\gamma}}^{-1}\widehat{\mathbf{R}}\widehat{\mathbf{S}}_{\boldsymbol{\gamma}}^{-1}\boldsymbol{\mu}_{\boldsymbol{\gamma}} + Tr(\xi^2\widehat{\mathbf{S}}_{\boldsymbol{\gamma}}^{-1}\widehat{\mathbf{R}}\widehat{\mathbf{S}}_{\boldsymbol{\gamma}}^{-1}\boldsymbol{\Sigma}_{\boldsymbol{\gamma}})\right\} + \beta_0\widehat{\boldsymbol{\Gamma}}^{\mathrm{T}}\widehat{\mathbf{S}}_{\boldsymbol{\Gamma}}^{-2}\boldsymbol{\mu}_{\boldsymbol{\gamma}} - \beta_0\boldsymbol{\mu}_{\boldsymbol{\alpha}}^{\mathrm{T}}\widehat{\mathbf{S}}_{\boldsymbol{\Gamma}}^{-1}\widehat{\mathbf{R}}\widehat{\mathbf{S}}_{\boldsymbol{\Gamma}}^{-1}\boldsymbol{\mu}_{\boldsymbol{\gamma}} \\
&+ \widehat{\boldsymbol{\Gamma}}^{\mathrm{T}}\widehat{\mathbf{S}}_{\boldsymbol{\Gamma}}^{-2}\boldsymbol{\mu}_{\boldsymbol{\alpha}} + \xi\widehat{\boldsymbol{\gamma}}^{\mathrm{T}}\widehat{\mathbf{S}}_{\boldsymbol{\gamma}}^{-2}\boldsymbol{\mu}_{\boldsymbol{\gamma}} - \frac{1}{2\sigma_{\boldsymbol{\gamma}}^2}\left\{\boldsymbol{\mu}_{\boldsymbol{\gamma}}^{\mathrm{T}}\boldsymbol{\mu}_{\boldsymbol{\gamma}} + Tr(\boldsymbol{\Sigma}_{\boldsymbol{\gamma}})\right\} - \frac{1}{2\sigma_{\boldsymbol{\alpha}}^2}\left\{\boldsymbol{\mu}_{\boldsymbol{\alpha}}^{\mathrm{T}}\boldsymbol{\mu}_{\boldsymbol{\alpha}} + Tr(\boldsymbol{\Sigma}_{\boldsymbol{\alpha}})\right\} \\
&- \frac{p}{2}\ln\sigma_{\boldsymbol{\gamma}}^2 - \frac{p}{2}\ln\sigma_{\boldsymbol{\alpha}}^2 + const(\boldsymbol{\theta}). \qquad \text{(S9)}
\end{aligned}
$$

In the M-step, we obtain the new updates for all the parameters $\boldsymbol{\theta}$ by setting the derivative of $E_{q(\boldsymbol{\gamma},\boldsymbol{\alpha})}\left\{\log\Pr(\widehat{\boldsymbol{\Gamma}},\widehat{\boldsymbol{\gamma}},\boldsymbol{\alpha},\boldsymbol{\gamma}|\widehat{\mathbf{S}}_{\boldsymbol{\gamma}},\widehat{\mathbf{S}}_{\boldsymbol{\Gamma}},\widehat{\mathbf{R}};\boldsymbol{\theta})\right\}$ to zeros. Thus, we have the following update equation

$$
\begin{aligned}
\beta_0 &= \left\{\boldsymbol{\mu}_{\boldsymbol{\gamma}}^{\mathrm{T}}\widehat{\mathbf{S}}_{\boldsymbol{\Gamma}}^{-1}\widehat{\mathbf{R}}\widehat{\mathbf{S}}_{\boldsymbol{\Gamma}}^{-1}\boldsymbol{\mu}_{\boldsymbol{\gamma}} + Tr(\widehat{\mathbf{S}}_{\boldsymbol{\Gamma}}^{-1}\widehat{\mathbf{R}}\widehat{\mathbf{S}}_{\boldsymbol{\Gamma}}^{-1}\boldsymbol{\Sigma}_{\boldsymbol{\gamma}})\right\}^{-1}\left(\widehat{\boldsymbol{\Gamma}}^{\mathrm{T}}\widehat{\mathbf{S}}_{\boldsymbol{\Gamma}}^{-2}\boldsymbol{\mu}_{\boldsymbol{\gamma}} - \boldsymbol{\mu}_{\boldsymbol{\alpha}}^{\mathrm{T}}\widehat{\mathbf{S}}_{\boldsymbol{\Gamma}}^{-1}\widehat{\mathbf{R}}\widehat{\mathbf{S}}_{\boldsymbol{\Gamma}}^{-1}\boldsymbol{\mu}_{\boldsymbol{\gamma}}\right), \\
\sigma_{\boldsymbol{\gamma}}^2 &= \left\{\boldsymbol{\mu}_{\boldsymbol{\gamma}}^{\mathrm{T}}\boldsymbol{\mu}_{\boldsymbol{\gamma}} + Tr(\boldsymbol{\Sigma}_{\boldsymbol{\gamma}})\right\}/p, \\
\sigma_{\boldsymbol{\alpha}}^2 &= \left\{\boldsymbol{\mu}_{\boldsymbol{\alpha}}^{\mathrm{T}}\boldsymbol{\mu}_{\boldsymbol{\alpha}} + Tr(\boldsymbol{\Sigma}_{\boldsymbol{\alpha}})\right\}/p, \\
\xi &= \left\{\boldsymbol{\mu}_{\boldsymbol{\gamma}}^{\mathrm{T}}\widehat{\mathbf{S}}_{\boldsymbol{\gamma}}^{-1}\widehat{\mathbf{R}}\widehat{\mathbf{S}}_{\boldsymbol{\gamma}}^{-1}\boldsymbol{\mu}_{\boldsymbol{\gamma}} + Tr(\widehat{\mathbf{S}}_{\boldsymbol{\gamma}}^{-1}\widehat{\mathbf{R}}\widehat{\mathbf{S}}_{\boldsymbol{\gamma}}^{-1}\boldsymbol{\Sigma}_{\boldsymbol{\gamma}})\right\}^{-1}\left(\widehat{\boldsymbol{\gamma}}^{\mathrm{T}}\widehat{\mathbf{S}}_{\boldsymbol{\gamma}}^{-2}\boldsymbol{\mu}_{\boldsymbol{\gamma}}\right), \qquad \text{(S10)}
\end{aligned}
$$

where $\boldsymbol{\mu}_{\boldsymbol{\gamma}} = (\mu_1,\cdots,\mu_p)^{\mathrm{T}}, \boldsymbol{\Sigma}_{\boldsymbol{\gamma}} = \mathrm{diag}((\sigma_1^2,\cdots,\sigma_p^2)^{\mathrm{T}}), \boldsymbol{\mu}_{\boldsymbol{\alpha}} = (\widetilde{\mu}_1,\cdots,\widetilde{\mu}_p)^{\mathrm{T}}, \boldsymbol{\Sigma}_{\boldsymbol{\alpha}} = \mathrm{diag}((\widetilde{\sigma}_1^2,\cdots,\widetilde{\sigma}_p^2)^{\mathrm{T}})$. Since the entropy of $q(\boldsymbol{\gamma})$ equals $-\frac{p}{2}\ln(2\pi) - \frac{1}{2}\sum_{j=1}^p \ln\sigma_j^2 - \frac{p}{2}$ and the entropy of $q(\boldsymbol{\alpha})$ equals $-\frac{p}{2}\ln(2\pi) - \frac{1}{2}\sum_{k=1}^p \ln\widetilde{\sigma}_k^2 - \frac{p}{2}$, the ELBO can be written by

$$
\begin{aligned}
&\mathcal{L}(\boldsymbol{\theta}) \\
&= E_{q(\boldsymbol{\gamma},\boldsymbol{\alpha})}\left\{\log\Pr(\widehat{\boldsymbol{\Gamma}},\widehat{\boldsymbol{\gamma}},\boldsymbol{\alpha},\boldsymbol{\gamma}|\widehat{\mathbf{S}}_{\boldsymbol{\gamma}},\widehat{\mathbf{S}}_{\boldsymbol{\Gamma}},\widehat{\mathbf{R}};\boldsymbol{\theta})\right\} - E_{q(\boldsymbol{\gamma})}\left\{\log q(\boldsymbol{\gamma})\right\} - E_{q(\boldsymbol{\alpha})}\left\{\log q(\boldsymbol{\alpha})\right\} \\
&= -\frac{1}{2}\beta_0^2\left\{\boldsymbol{\mu}_{\boldsymbol{\gamma}}^{\mathrm{T}}\widehat{\mathbf{S}}_{\boldsymbol{\Gamma}}^{-1}\widehat{\mathbf{R}}\widehat{\mathbf{S}}_{\boldsymbol{\Gamma}}^{-1}\boldsymbol{\mu}_{\boldsymbol{\gamma}} + Tr(\widehat{\mathbf{S}}_{\boldsymbol{\Gamma}}^{-1}\widehat{\mathbf{R}}\widehat{\mathbf{S}}_{\boldsymbol{\Gamma}}^{-1}\boldsymbol{\Sigma}_{\boldsymbol{\gamma}})\right\} - \frac{1}{2}\left\{\boldsymbol{\mu}_{\boldsymbol{\alpha}}^{\mathrm{T}}\widehat{\mathbf{S}}_{\boldsymbol{\Gamma}}^{-1}\widehat{\mathbf{R}}\widehat{\mathbf{S}}_{\boldsymbol{\Gamma}}^{-1}\boldsymbol{\mu}_{\boldsymbol{\alpha}} + Tr(\widehat{\mathbf{S}}_{\boldsymbol{\Gamma}}^{-1}\widehat{\mathbf{R}}\widehat{\mathbf{S}}_{\boldsymbol{\Gamma}}^{-1}\boldsymbol{\Sigma}_{\boldsymbol{\alpha}})\right\} \\
&- \frac{1}{2}\left\{\xi^2\boldsymbol{\mu}_{\boldsymbol{\gamma}}^{\mathrm{T}}\widehat{\mathbf{S}}_{\boldsymbol{\gamma}}^{-1}\widehat{\mathbf{R}}\widehat{\mathbf{S}}_{\boldsymbol{\gamma}}^{-1}\boldsymbol{\mu}_{\boldsymbol{\gamma}} + Tr(\xi^2\widehat{\mathbf{S}}_{\boldsymbol{\gamma}}^{-1}\widehat{\mathbf{R}}\widehat{\mathbf{S}}_{\boldsymbol{\gamma}}^{-1}\boldsymbol{\Sigma}_{\boldsymbol{\gamma}})\right\} + \beta_0\widehat{\boldsymbol{\Gamma}}^{\mathrm{T}}\widehat{\mathbf{S}}_{\boldsymbol{\Gamma}}^{-2}\boldsymbol{\mu}_{\boldsymbol{\gamma}} - \beta_0\boldsymbol{\mu}_{\boldsymbol{\alpha}}^{\mathrm{T}}\widehat{\mathbf{S}}_{\boldsymbol{\Gamma}}^{-1}\widehat{\mathbf{R}}\widehat{\mathbf{S}}_{\boldsymbol{\Gamma}}^{-1}\boldsymbol{\mu}_{\boldsymbol{\gamma}} \\
&+ \widehat{\boldsymbol{\Gamma}}^{\mathrm{T}}\widehat{\mathbf{S}}_{\boldsymbol{\Gamma}}^{-2}\boldsymbol{\mu}_{\boldsymbol{\alpha}} + \xi\widehat{\boldsymbol{\gamma}}^{\mathrm{T}}\widehat{\mathbf{S}}_{\boldsymbol{\gamma}}^{-2}\boldsymbol{\mu}_{\boldsymbol{\gamma}} - \frac{1}{2\sigma_{\boldsymbol{\gamma}}^2}\left\{\boldsymbol{\mu}_{\boldsymbol{\gamma}}^{\mathrm{T}}\boldsymbol{\mu}_{\boldsymbol{\gamma}} + Tr(\boldsymbol{\Sigma}_{\boldsymbol{\gamma}})\right\} - \frac{1}{2\sigma_{\boldsymbol{\alpha}}^2}\left\{\boldsymbol{\mu}_{\boldsymbol{\alpha}}^{\mathrm{T}}\boldsymbol{\mu}_{\boldsymbol{\alpha}} + Tr(\boldsymbol{\Sigma}_{\boldsymbol{\alpha}})\right\} \\
&- \frac{p}{2}\ln\sigma_{\boldsymbol{\gamma}}^2 - \frac{p}{2}\ln\sigma_{\boldsymbol{\alpha}}^2 + \frac{1}{2}\sum_{j=1}^p \ln\sigma_j^2 + \frac{1}{2}\sum_{k=1}^p \ln\widetilde{\sigma}_k^2 + const(\boldsymbol{\theta}).
\end{aligned}
$$

The reduction steps: $\boldsymbol{\mu}_{\boldsymbol{\gamma}} = \xi\boldsymbol{\mu}_{\boldsymbol{\gamma}},\ \beta_0 = \beta_0/\xi,\ \sigma_{\boldsymbol{\gamma}}^2 = \xi^2\sigma_{\boldsymbol{\gamma}}^2.$

The corresponding PX-VBEM for MR-LDP can be summarized as Algorithm 2.

Algorithm 2: PX-VBEM for MR-LDP

Initialization: $\beta_0 = 0, \boldsymbol{\mu}_{\boldsymbol{\gamma}} = \boldsymbol{\mu}_{\boldsymbol{\alpha}} = \mathbf{0}$, $\sigma^2_{\boldsymbol{\alpha}} = \sigma^2_{\boldsymbol{\gamma}} = 0.01$ and $\xi = 1$.

repeat

  E-step: At the t-th iteration, for $j = 1, \cdots, p$ and $k = 1, \cdots, p$, both the posterior distribution of $q(\gamma_j|\boldsymbol{\theta}^{(t)})$ and $q(\alpha_k|\boldsymbol{\theta}^{(t)})$ are Gaussian with expressions (S7) and (S8). The parameters are given as $\xi = \xi^{(t)} = 1$, $\beta_0 = \beta_0^{(t)}$, $\sigma^2_{\boldsymbol{\gamma}} = (\sigma^{(t)}_{\boldsymbol{\gamma}})^2$ and $\sigma^2_{\boldsymbol{\alpha}} = (\sigma^{(t)}_{\boldsymbol{\alpha}})^2$.

  M-tesp: Update β_0, $\sigma^2_{\boldsymbol{\gamma}}$, $\sigma^2_{\boldsymbol{\alpha}}$ and ξ as equation(S10).

  Reduction-step: Rescale the parameters $\boldsymbol{\mu}^{(t+1)}_{\boldsymbol{\gamma}} = \xi^{(t+1)}\boldsymbol{\mu}^{(t+1)}_{\boldsymbol{\gamma}}$, $\beta_0^{(t+1)} = \beta_0^{(t+1)}/\xi^{(t+1)}$, $(\sigma_j^{(t+1)})^2 = (\xi^{(t+1)})^2(\sigma_j^{(t+1)})^2$ and reset $\xi^{(t+1)} = 1$.

until *convergence or maximum iteration reached*;

2.2.3 Statistical Inference for MR-LDP

The procedure of statistical inference for MR-LDP is similar with MR-LD, we first calibrate the ELBO(denoted by $\widetilde{\mathcal{L}}(\boldsymbol{\theta}, \boldsymbol{\mu}_{\gamma}, \boldsymbol{\mu}_{\boldsymbol{\alpha}})$) as follows

$$
\begin{aligned}
& \widetilde{\mathcal{L}}(\boldsymbol{\theta}, \boldsymbol{\mu}_{\gamma}, \boldsymbol{\mu}_{\boldsymbol{\alpha}}) \\
= \; & E_{q(\boldsymbol{\gamma},\boldsymbol{\alpha})}\left\{\log \Pr(\widehat{\boldsymbol{\Gamma}}, \widehat{\boldsymbol{\gamma}}, \boldsymbol{\alpha}, \boldsymbol{\gamma}|\widehat{\mathbf{S}}_{\gamma}, \widehat{\mathbf{S}}_{\boldsymbol{\Gamma}}, \widehat{\mathbf{R}}; \boldsymbol{\theta})\right\} - E_{q(\boldsymbol{\gamma})}\left\{\log q(\boldsymbol{\gamma})\right\} - E_{q(\boldsymbol{\alpha})}\left\{\log q(\boldsymbol{\alpha})\right\} \\
= \; & \left(\beta_0 \widehat{\boldsymbol{\Gamma}}^{\mathrm{T}} \widehat{\mathbf{S}}_{\boldsymbol{\Gamma}}^{-2} + \xi \widehat{\boldsymbol{\gamma}} \widehat{\mathbf{S}}_{\gamma}^{-2}\right) \boldsymbol{\mu}_{\gamma} - \frac{1}{2}\boldsymbol{\mu}_{\gamma}^{\mathrm{T}} \widetilde{\boldsymbol{\Sigma}}_{\gamma}^{-1} \boldsymbol{\mu}_{\gamma} + \widehat{\boldsymbol{\Gamma}} \widehat{\mathbf{S}}_{\boldsymbol{\Gamma}}^{-2} \boldsymbol{\mu}_{\boldsymbol{\alpha}} - \frac{1}{2}\boldsymbol{\mu}_{\boldsymbol{\alpha}}^{\mathrm{T}} \widetilde{\boldsymbol{\Sigma}}_{\boldsymbol{\alpha}}^{-1} \boldsymbol{\mu}_{\boldsymbol{\alpha}} - \beta_0 \boldsymbol{\mu}_{\boldsymbol{\alpha}}^{\mathrm{T}} \widehat{\mathbf{S}}_{\boldsymbol{\Gamma}}^{-1} \widehat{\mathbf{R}} \widehat{\mathbf{S}}_{\boldsymbol{\Gamma}}^{-1} \boldsymbol{\mu}_{\gamma} \\
- \; & \frac{p}{2}(\log \sigma^2_{\boldsymbol{\gamma}} + 1) - \frac{p}{2}(\log \sigma^2_{\boldsymbol{\alpha}} + 1) + \frac{1}{2}\log|\widetilde{\boldsymbol{\Sigma}}_{\gamma}| + \frac{1}{2}\log|\widetilde{\boldsymbol{\Sigma}}_{\boldsymbol{\alpha}}| + p - p\log(2\pi) \\
- \; & \frac{1}{2}\left\{\log|\widehat{\mathbf{S}}_{\gamma}\widehat{\mathbf{R}}\widehat{\mathbf{S}}_{\gamma}| + \log|\widehat{\mathbf{S}}_{\boldsymbol{\Gamma}}\widehat{\mathbf{R}}\widehat{\mathbf{S}}_{\boldsymbol{\Gamma}}| + \widehat{\boldsymbol{\gamma}}^{\mathrm{T}}(\widehat{\mathbf{S}}_{\gamma}\widehat{\mathbf{R}}\widehat{\mathbf{S}}_{\gamma})^{-1}\widehat{\boldsymbol{\gamma}} + \widehat{\boldsymbol{\Gamma}}^{\mathrm{T}}(\widehat{\mathbf{S}}_{\boldsymbol{\Gamma}}\widehat{\mathbf{R}}\widehat{\mathbf{S}}_{\boldsymbol{\Gamma}})^{-1}\boldsymbol{\Gamma}\right\}, \qquad \text{(S11)}
\end{aligned}
$$

where $\boldsymbol{\mu}_{\gamma}$ and $\widetilde{\boldsymbol{\Sigma}}_{\gamma}$ are the posterior mean and posterior variance for latent variable $\boldsymbol{\gamma}$, $\boldsymbol{\mu}_{\boldsymbol{\alpha}}$ and $\widetilde{\boldsymbol{\Sigma}}_{\boldsymbol{\alpha}}$ are the posterior mean and posterior variance for latent variable $\boldsymbol{\alpha}$, the forms of $\widetilde{\boldsymbol{\Sigma}}_{\gamma}$) and $\widetilde{\boldsymbol{\Sigma}}_{\boldsymbol{\alpha}}$) are from EM/PX-EM by plugging the posterior mean estimates and parameter estimates from VBEM/PX-VBEM which can be expressed as

$$
\widetilde{\boldsymbol{\Sigma}}_{\gamma} \;=\; \left(\beta_0^2 \widehat{\mathbf{S}}_{\boldsymbol{\Gamma}}^{-1} \widehat{\mathbf{R}} \widehat{\mathbf{S}}_{\boldsymbol{\Gamma}}^{-1} + \xi^2 \widehat{\mathbf{S}}_{\gamma}^{-1} \widehat{\mathbf{R}} \widehat{\mathbf{S}}_{\gamma}^{-1} + \sigma_{\boldsymbol{\gamma}}^{-2}\mathbf{I}\right)^{-1},
$$

and

$$\widetilde{\boldsymbol{\Sigma}}_{\boldsymbol{\alpha}} = \left(\widehat{\mathbf{S}}_{\boldsymbol{\Gamma}}^{-1}\widehat{\mathbf{R}}\widehat{\mathbf{S}}_{\boldsymbol{\Gamma}}^{-1} + \sigma_{\boldsymbol{\alpha}}^{-2}\mathbf{I}\right)^{-1}.$$

Similarly, we recalibrate the ELBO for MR-LDP as follows:

1. Obtain parameters together with variational means under both $\mathcal{H}_0$ and $\mathcal{H}_1$.
2. Calibrate ELBO using formulae (S11).
3. Calculate the test statistic (S4) using the calibrated ELBO as a proxy to the marginal log-likelihood.

3 More simulation results for different settings

The results of type-I error and point estimates for the dense pleiotropy with $n_3 = 2,500; 4,000$ are shown in Figure S2 - S3, respectively. For sparse horizontal pleiotropy, we consider the sparsity level at 0.2 and 0.4. Figure S4 and S5 show the results of type-I error and point estimates for the sparse pleiotropy at 0.2 with $n_3 = 2,500; 4,000$, respectively, and Figures-S6 - S8 show the results of type-I error and point estimates with $n_3 = 500; 2,500; 4,000$, respectively, for the sparse pleiotropy at 0.4.

To show robustness of proposed methods, we conducted additional simulations using non-generative distributions. Specifically, for each $j = 1, \cdots, p$, both γ_j and α_j are from t-distributions with degrees of freedom either at 5 or 10. The result can be found in Figure S9. As one can see, our method still controls type-I error at nominal level 0.05 while point estimates are roughly unbiased.

Moreover, we conducted simulations to make comparisons of statistical power for all methods in Figure S10, where we varied heritability of indirect effect $\boldsymbol{\gamma}$ with $h^2_{\boldsymbol{\gamma}} \in \{0, 0.01, 0.02, 0.03, 0.04, 0.05\}$. As statistical power is only meaningful when type-I error is under control, we conducted SNPs pruning for alternative methods to ensure that their type-I error is controlled at 0.05 level for null. As shown in Figure S10, we controlled type-I error at its nominal level 0.05 under the null when $h^2_{\boldsymbol{\gamma}} = 0$. Moreover, one can observe that when there is no horizontal pleiotropy, power for MR-LD and MR-LDP are similar. When their exists horizontal pleiotropy, MR-LD cannot control type-I error and thus the power is inflated but MR-LDP still controls type-I error at nominal level with improved power. Finally, statistical power from MR-LDP outperforms those from alternative methods especially when LD is high.

To mimic the real world, we adopted a screening dataset to select the significant SNPs in the following simulation study. In details, the screening dataset are generated in the way as

the exposure dataset,

$$\mathbf{x}_s = \mathbf{G}_s\boldsymbol{\gamma}_s + \mathbf{U}_s\eta_s + \mathbf{e}_s$$

where $\mathbf{G}_s, \boldsymbol{\gamma}_s, \mathbf{U}_s, \eta_s$ and $\mathbf{e}_s$ are independent and identically distributed with their corresponding counterparts, $\mathbf{G}_1, \boldsymbol{\gamma}, \mathbf{U}_x, \eta_1$ and $\mathbf{e}_1$. The genotype matrix is $\mathbf{G}_s \in \mathbb{R}^{n_0 \times p}$ with $n_0 = 20,000, p = 20,000$. The effect size $\boldsymbol{\gamma}_s$ is a p-dimensional vector, where 90% of $\boldsymbol{\gamma}$ are zero and the rest nonzero entries of $\boldsymbol{\gamma}$ were simulated from $\mathcal{N}(0, \sigma^2_{\boldsymbol{\gamma}})$. This setting is realistic as [6] argued that complex traits are highly polygenic, e.g., for human height, there are around 10% of all SNPs associated with it. After conducting the single-variant analysis between $\mathbf{G}_s$ and $\mathbf{x}_s$, we selected the genetic variants with p-value less than 0.01. The corresponding summary statistics for the selected genetic instruments in both SNP-exposure and SNP-outcome were chosen to complete the subsequent MR analysis. As shown in Figure S11, MR-LD and MR-LDP perform equally well in type-I error control in the case of no horizontal pleiotropy. When horizontal pleiotropy is getting larger ($h^2_{\boldsymbol{\alpha}} = 0.05$ or 0.1), MR-LDP is still capable of controlling type-I error at its nominal level. In addition, both our methods and GSMR are unbiased in point estimates while our methods have smaller standard errors.

In addition, we consider the binary outcome in the following simulations. In details, we generated data for binary outcome using the following logistic model:

$$\mathbf{x} = \mathbf{G}_1\boldsymbol{\gamma} + \mathbf{U}_x\boldsymbol{\eta}_x + \mathbf{e}_1, \quad \mathbf{y} = Bernoulli(H(\beta\mathbf{x} + \mathbf{G}_2\boldsymbol{\alpha} + \mathbf{U}_y\boldsymbol{\eta}_y)),$$

where $H(t) = 1/(1 + \exp(-t))$.

The population prevalence was set to be 0.1. We first generated a large population pool of outcomes and sampled 10,000 cases and 10,000 controls for the following analysis. Since the odds ratio is not collapsible, the original estimation of casual effect is biased [10], in light of [7] and [18], we should correct the casual effect using the formula $\beta_0 = \beta/\sqrt{1 + (\sigma/1.7)^2}$, where σ^2 can be estimated using $var((\beta\mathbf{x} + \mathbf{G}_2\boldsymbol{\alpha} + \mathbf{U}_y\boldsymbol{\eta}_y)$. We display the result of simulation study

in Figure S12. Clearly, the results are generally similar to those in quantitative traits. Only MR-LDP can control type-I error when there are both strong LD and horizontal pleiotropy. The standard errors of MR-LDP is smaller due to the inclusion of all SNPs within LD.

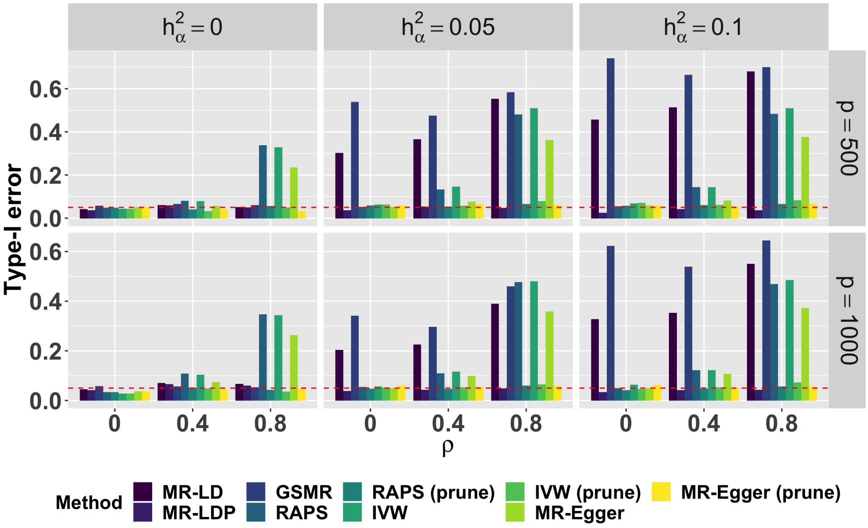

(A) Type-I error

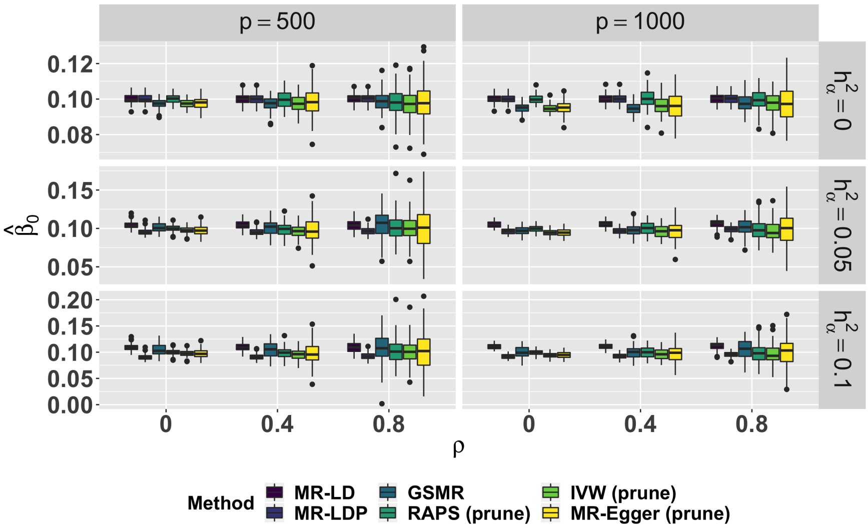

(B) Point estimates for β_0

Figure S2: Simulation of type-I error control and point estimates under the dense horizontal pleiotropy. $n_1 = n_2 = 20,000, n_3 = 2,500$.

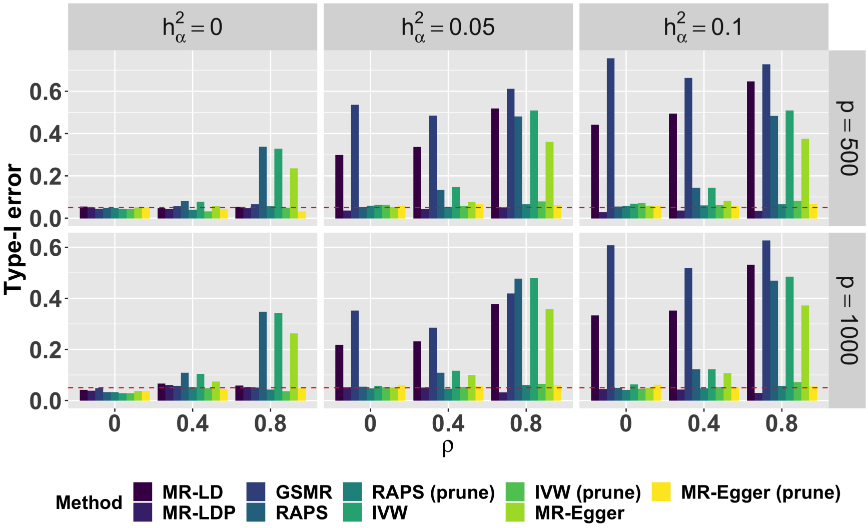

(A) Type-I error

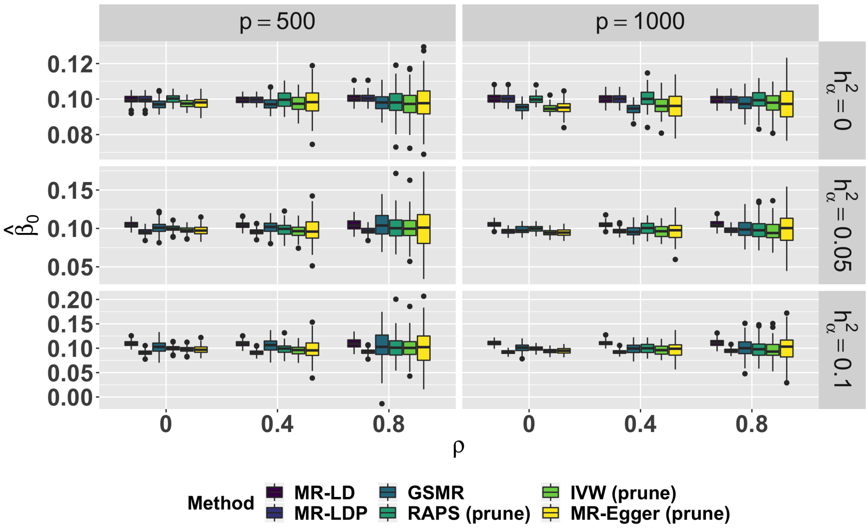

(B) Point estimates for β_0

Figure S3: Simulation of type-I error control and point estimates under the dense horizontal pleiotropy. $n_1 = n_2 = 20,000, n_3 = 4,000$.

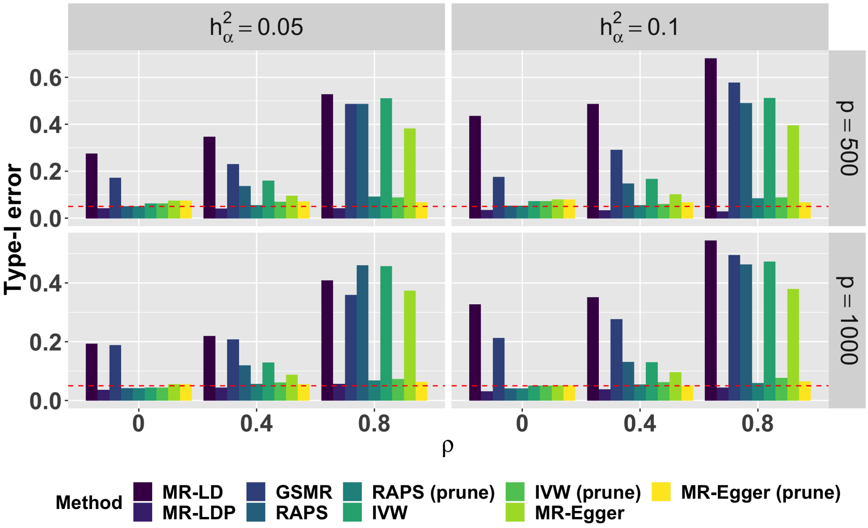

(A) Type-I error

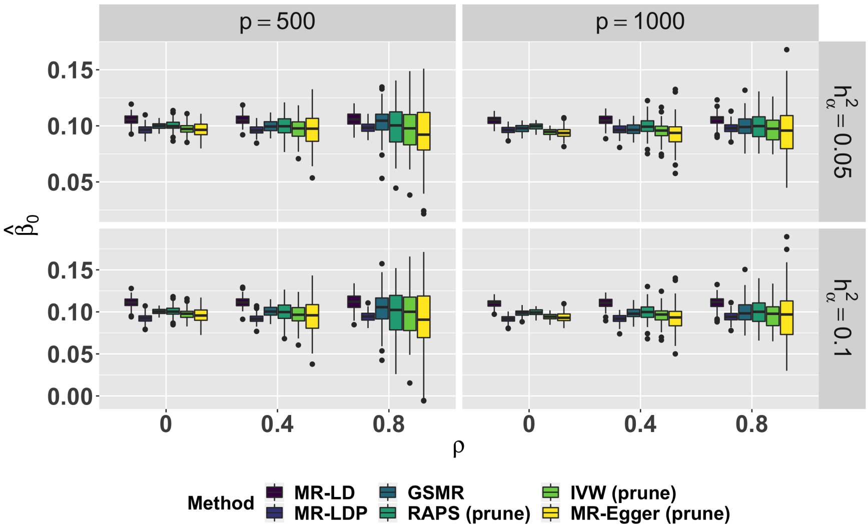

(B) Point estimates for β_0

Figure S4: Simulation of type-I error control and point estimates under the sparse horizontal pleiotropy, sparsity = 0.2. $n_1 = n_2 = 20,000, n_3 = 2,500$.

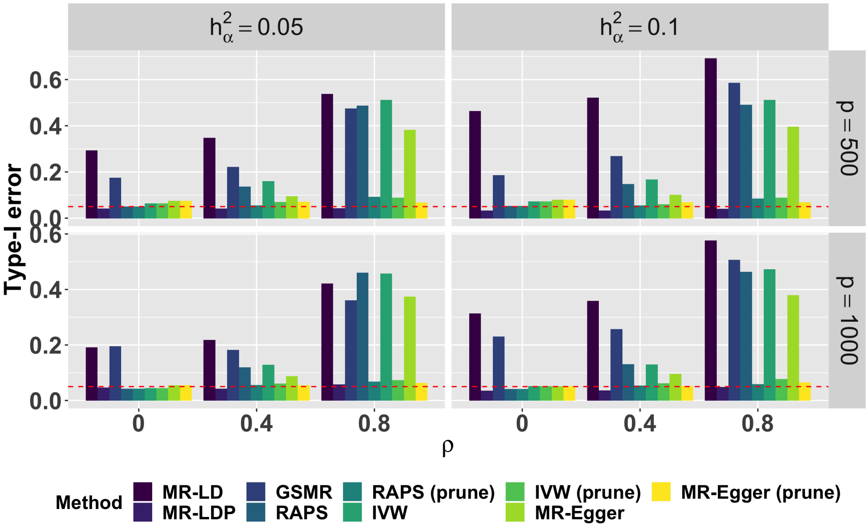

(A) Type-I error

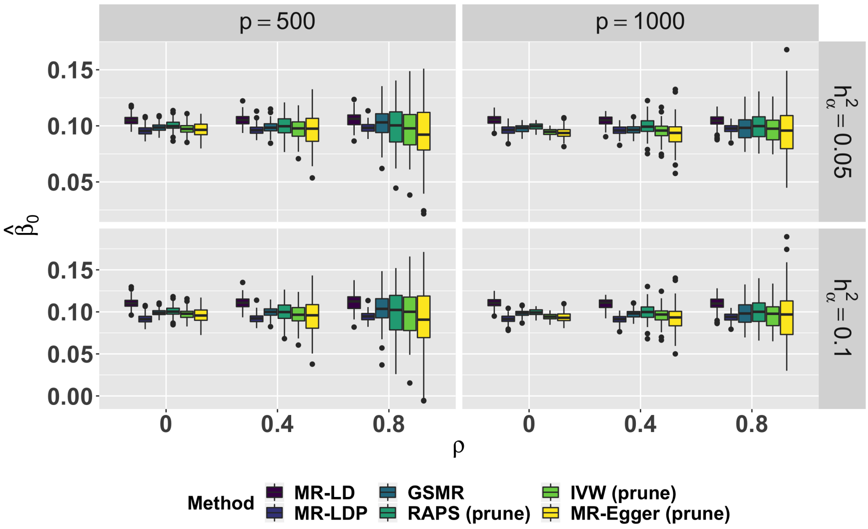

(B) Point estimates for β_0

Figure S5: Simulation of type-I error control and point estimates under the sparse horizontal pleiotropy, sparsity = 0.2. $n_1 = n_2 = 20,000, n_3 = 4,000$.

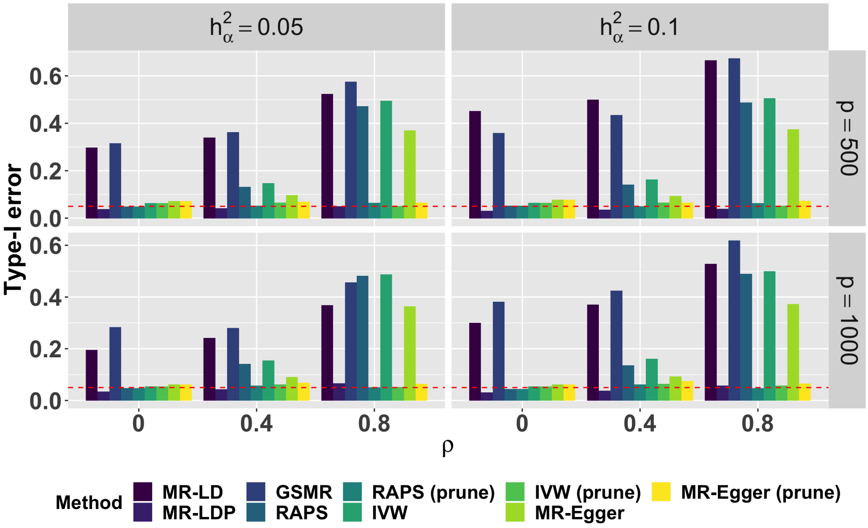

(A) Type-I error

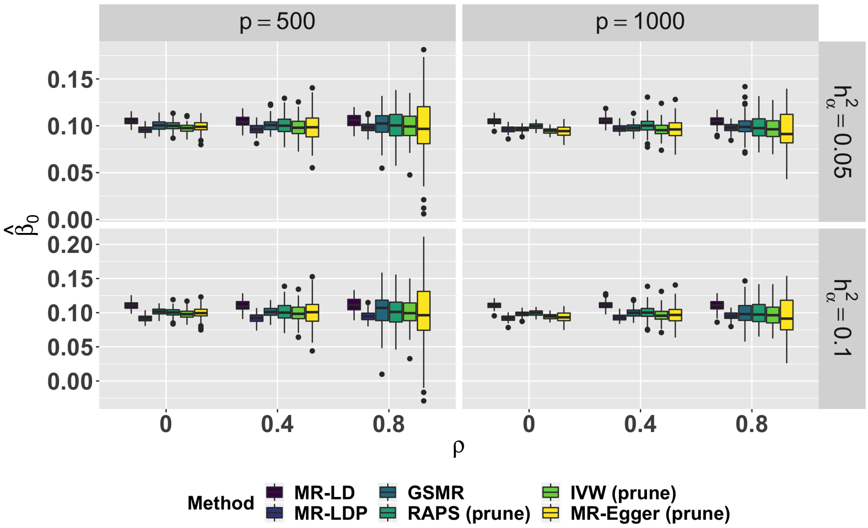

(B) Point estimates for β_0

Figure S6: Simulation of type-I error control and point estimates under the sparse horizontal pleiotropy, sparsity $= 0.4$. $n_1 = n_2 = 20,000, n_3 = 500$.

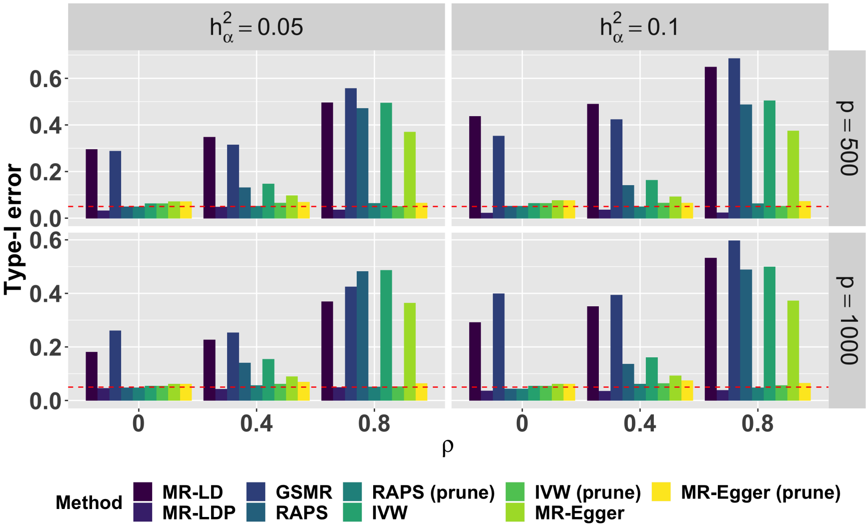

(A) Type-I error

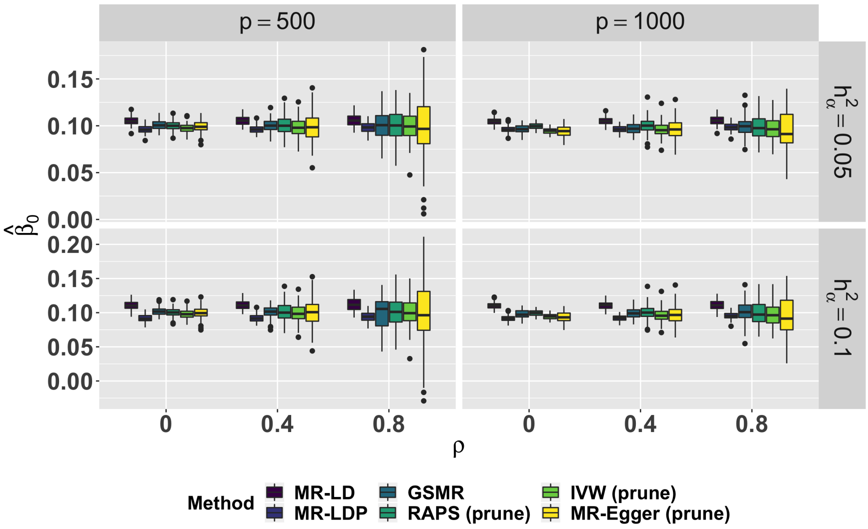

(B) Point estimates for β_0

Figure S7: Simulation of type-I error control and point estimates under the sparse horizontal pleiotropy, sparsity $= 0.4$. $n_1 = n_2 = 20,000, n_3 = 2,500$.

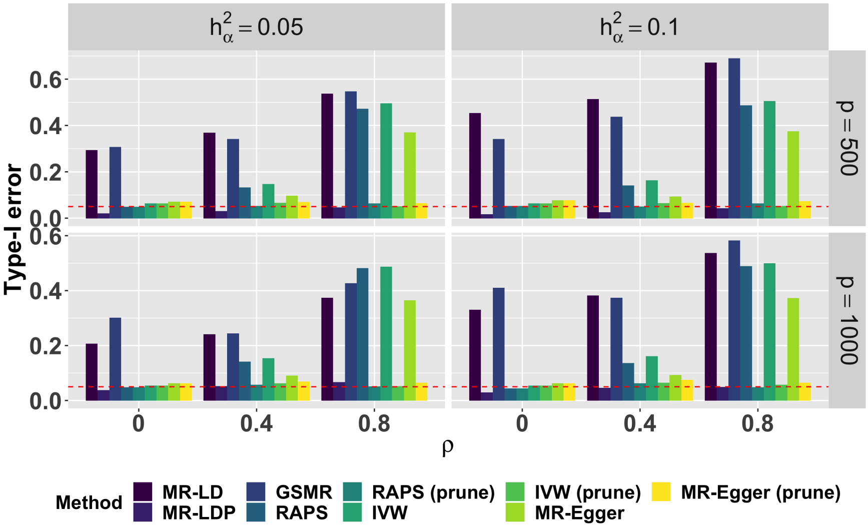

(A) Type-I error

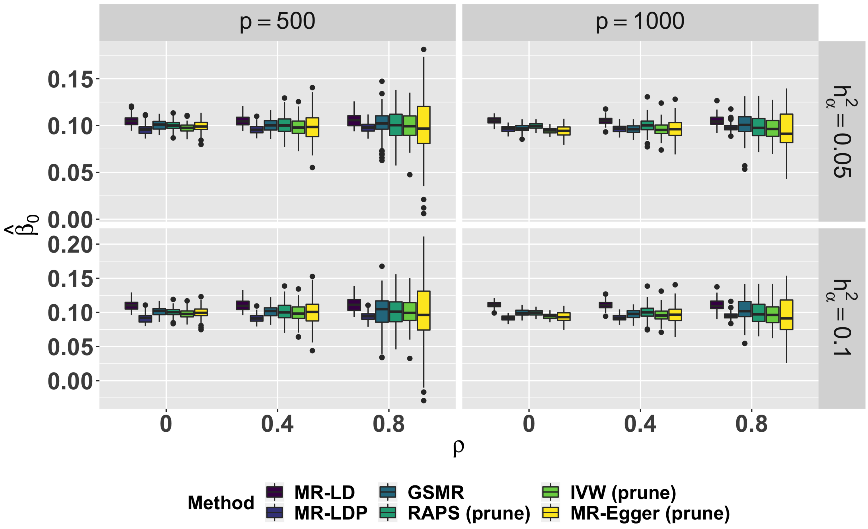

(B) Point estimates for β_0

Figure S8: Simulation of type-I error control and point estimates under the sparse horizontal pleiotropy, sparsity = 0.4. $n_1 = n_2 = 20,000, n_3 = 4,000$.

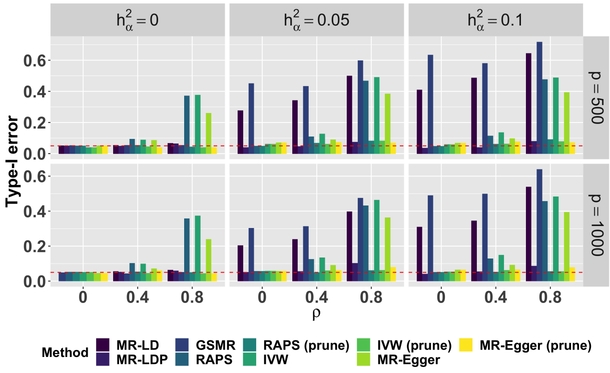

(A) Type-I error ($\nu = 5$)

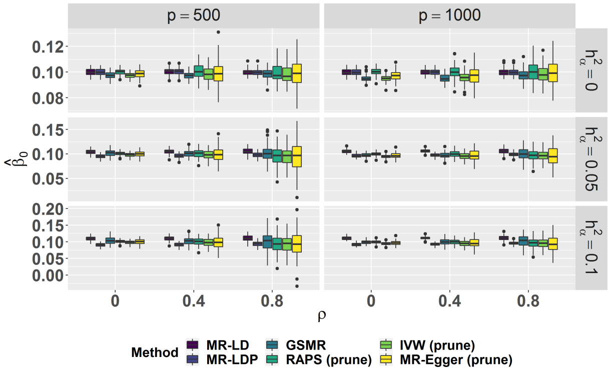

(B) Point estimates for β_0 ($\nu = 5$)

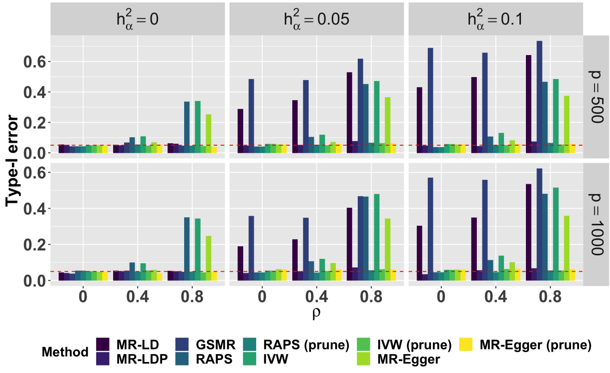

(C) Type-I error ($\nu = 10$)

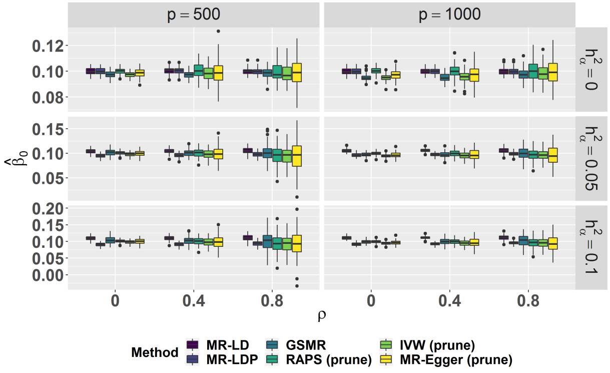

(D) Point estimates for β_0 ($\nu = 10$)

Figure S9: Simulation of type-I error control and point estimates under the dense horizontal pleiotropy. $n_1 = n_2 = 20,000, n_3 = 500$, and degrees of freedom $\nu = 5$ or 10.

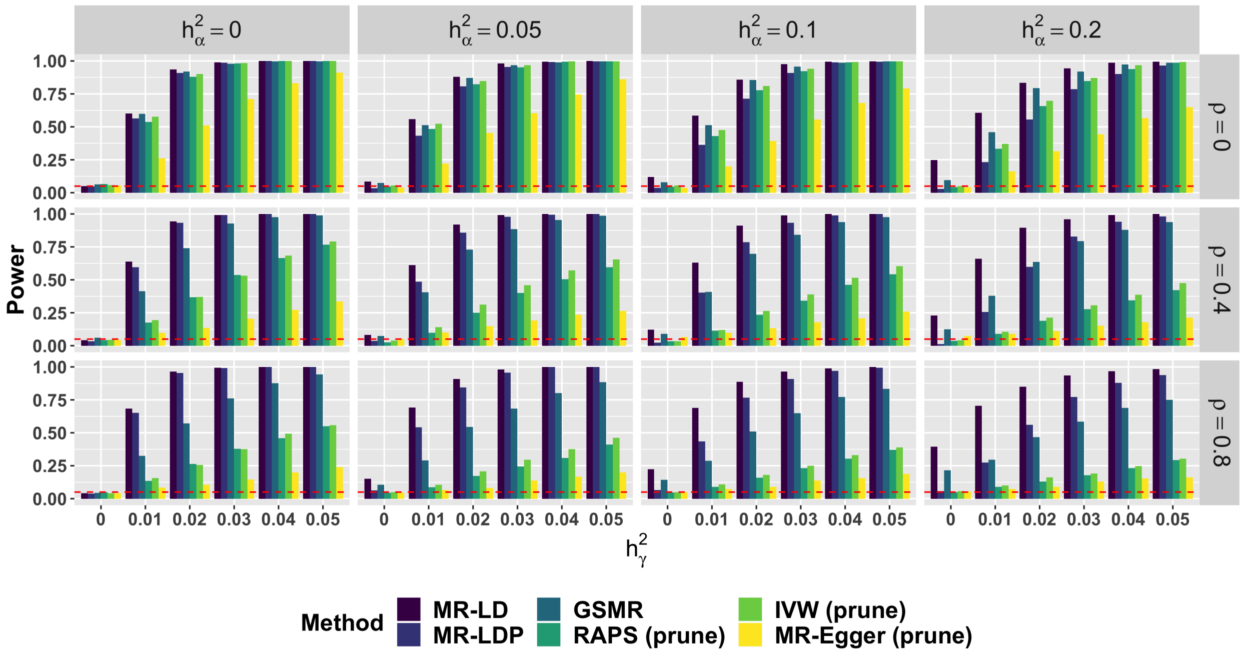

Figure S10: Simulation of statistical power under dense horizontal pleiotropy. $n_1 = n_2 = 1,000, n_3 = 500, p = 500$.

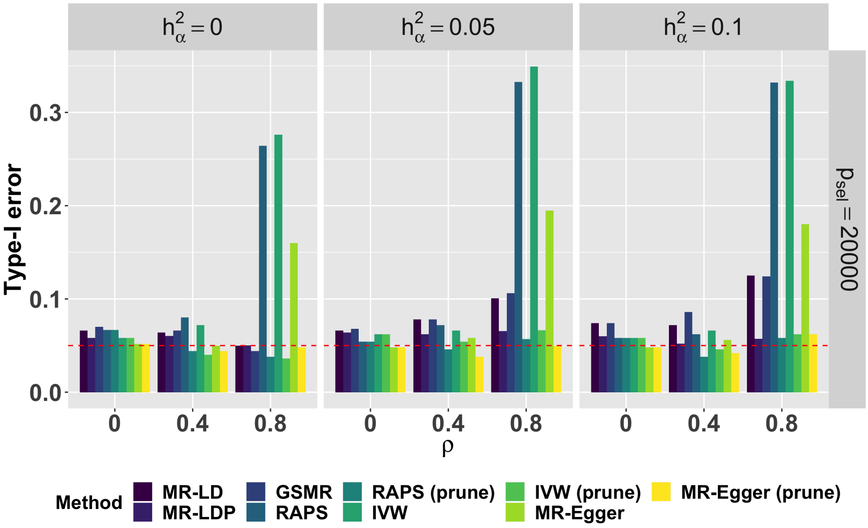

(A) Type-I error

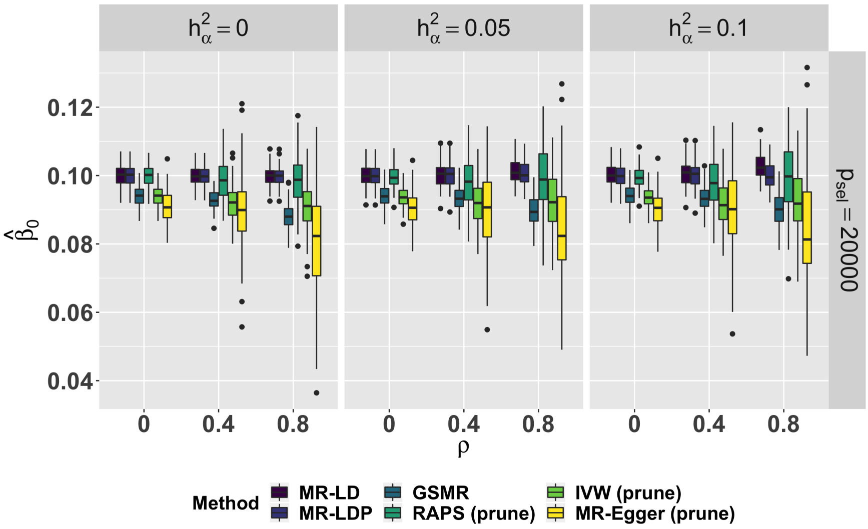

(B) Point estimates for β_0

Figure S11: Simulation of type-I error control and point estimates under the screening process. $n_0 = n_1 = n_2 = 20,000, n_3 = 500$.

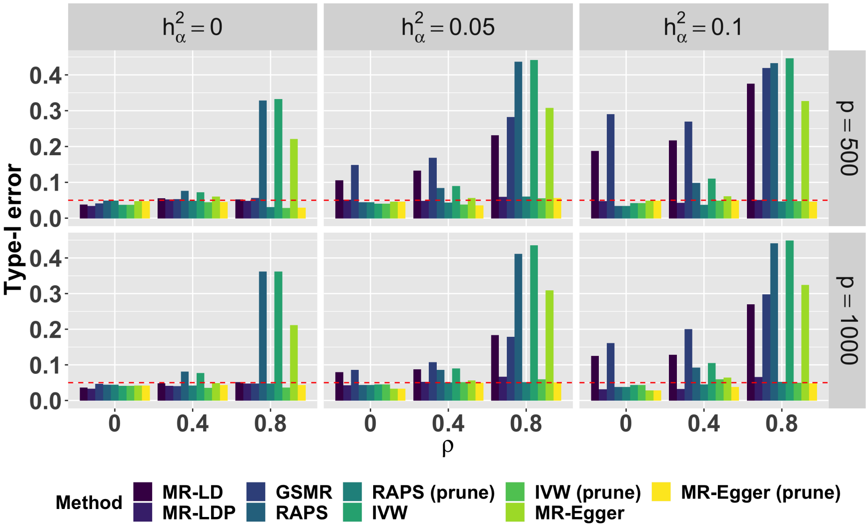

(A) Type-I error

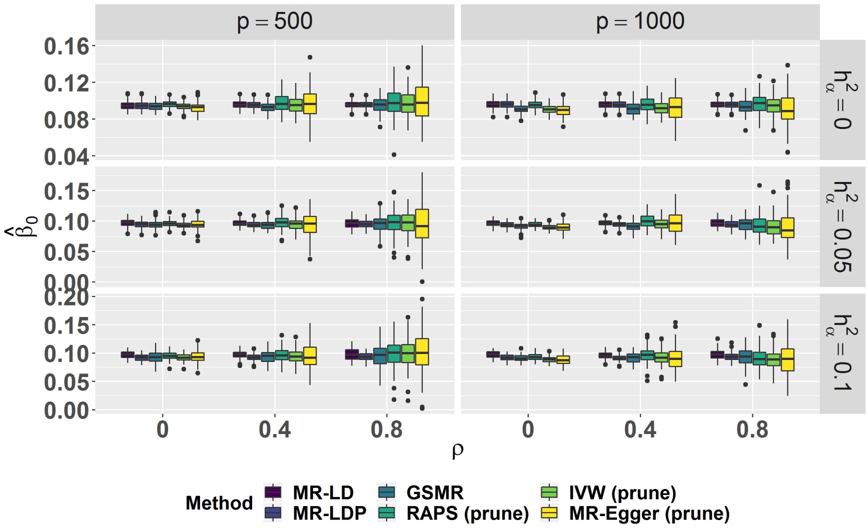

(B) Point estimates for β_0

Figure S12: Simulation of type-I error control and point estimates under the binary outcome. $n_1 = n_2 = 20,000, n_3 = 500$.

4 Real Data Analysis

4.1 Two validation studies

4.1.1 CAD-CAD study

We display the scatter plot of $\widehat{\boldsymbol{\gamma}}$ (C4D) against $\widehat{\boldsymbol{\Gamma}}$ (CAD1) in Figure S13, each point is augmented by the standard error of $\widehat{\gamma}_i$ and $\widehat{\Gamma}_i$ on the vertical and horizontal sides. In Figure S14, we report detailed results of CAD-CAD study with the shrinkage parameter $\lambda = 0.15$.

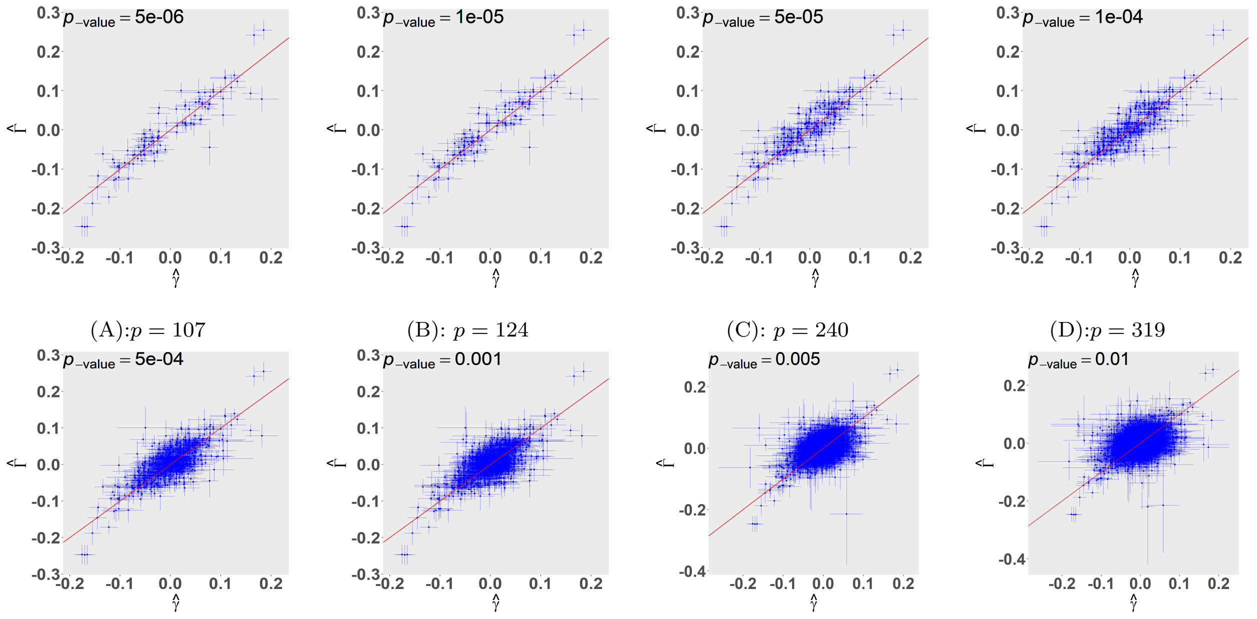

(A):$p = 107$ (B): $p = 124$ (C): $p = 240$ (D):$p = 319$

(E): $p = 780$ (F): $p = 1207$ (G): $p = 3944$ (H):$p = 6965$

Figure S13: Scatter plots of $\widehat{\boldsymbol{\gamma}}$ (C4D) against $\widehat{\boldsymbol{\Gamma}}$ (CAD1) with a sequence of p-value thresholds in the screening dataset.

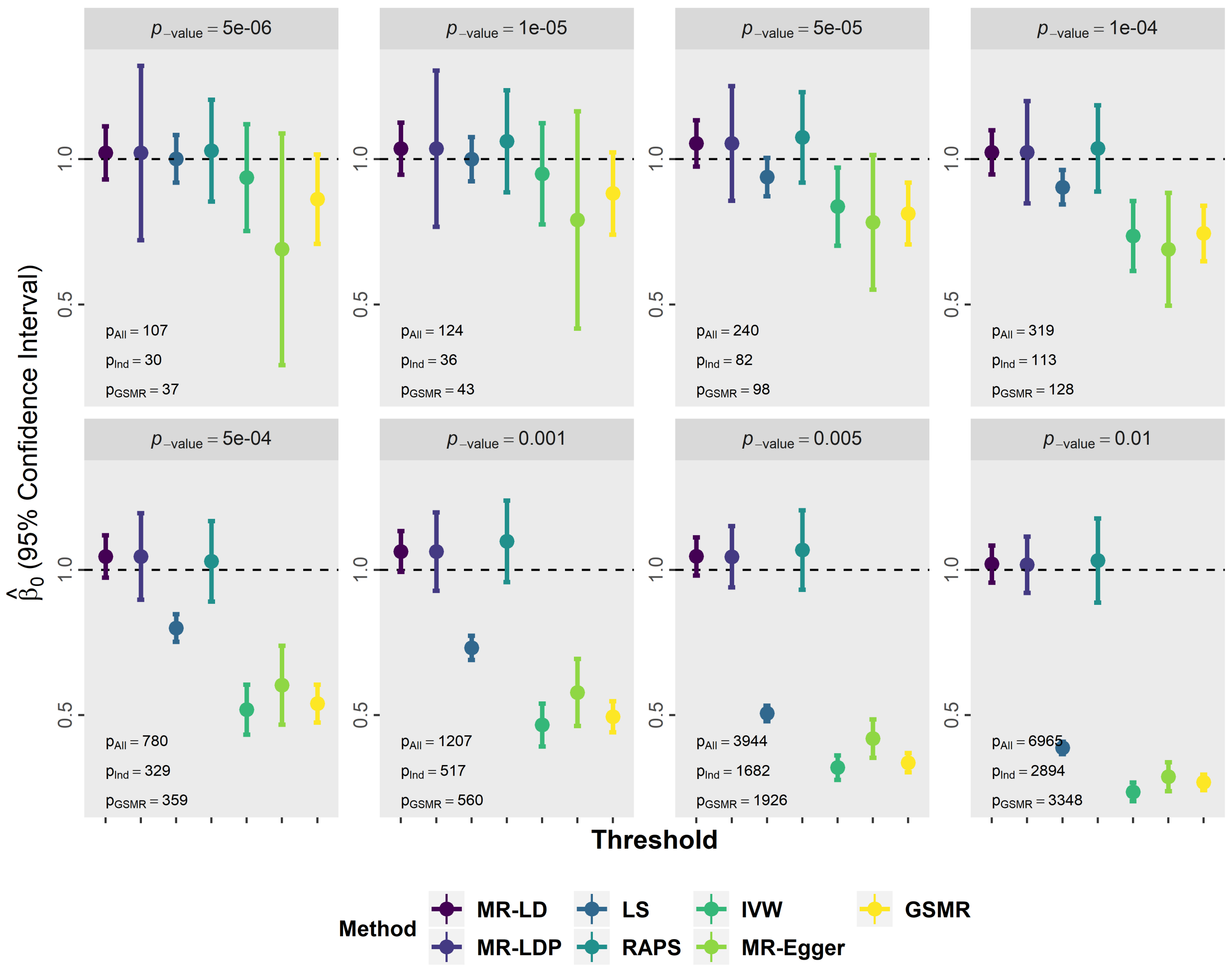

Figure S14: The result of CAD-CAD using UK10K as the reference penal with shrinkage parameter $\lambda = 0.15$ under different p-value thresholds to choose genetic variants in the screening dataset, e.g., p-value = 5e-6, 1e-05. MR-LD, MR-LDP and LS methods use all SNPs selected by the screening dataset (denoted as P_{All}), but IVW, MR-Egger, RAPS and GSMR use pruned SNPs, where the default value of r^2 is used for GSMR (the number of SNP used: P_{GSMR}) and $r^2 = 0.001$ is used for IVW, MR-Egger and RAPS (the number of SNP used: P_{Ind}).

4.1.2 Height-Height study

We display the scatter plot of $\widehat{\boldsymbol{\gamma}}$ (height for males) against $\widehat{\boldsymbol{\Gamma}}$ (height for females) in Figure S15, each point is augmented by the standard error of $\widehat{\gamma}_i$ and $\widehat{\Gamma}_i$ on the vertical and horizontal sides. In Figures S16 and S17, we report detailed results of Height-Height study with the shrinkage parameters $\lambda = 0.1$ and $\lambda = 0.15$, respectively.

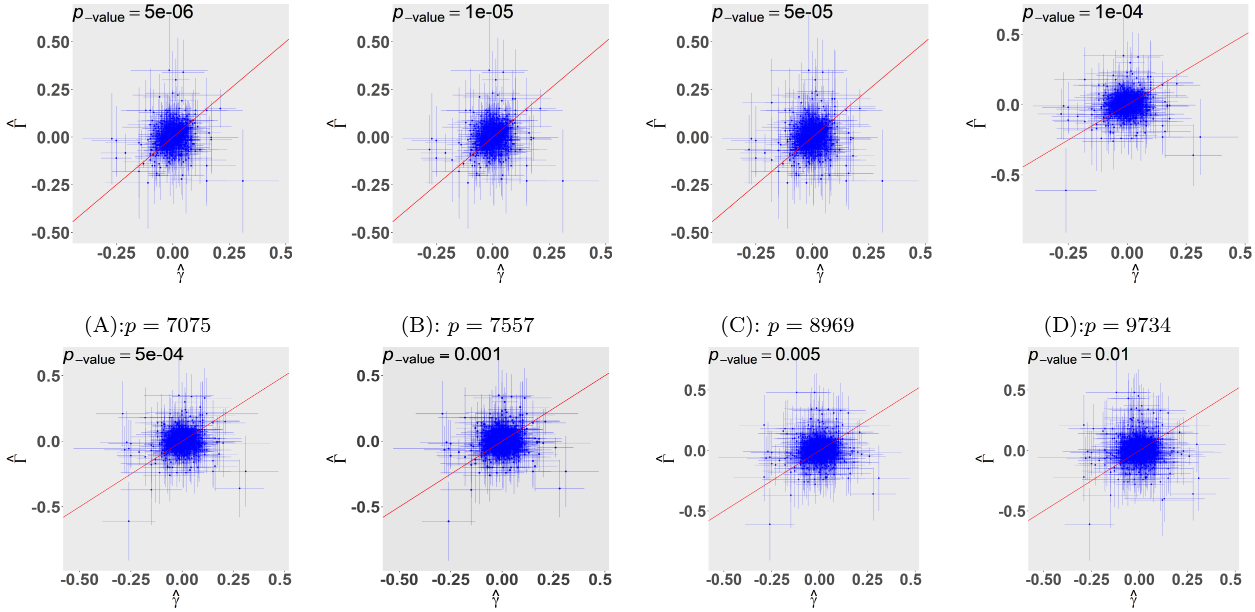

(A): $p = 7075$ (B): $p = 7557$ (C): $p = 8969$ (D): $p = 9734$

(E): $p = 12165$ (F): $p = 13539$ (G): $p = 18059$ (H): $p = 20947$

Figure S15: Scatter plot of $\widehat{\boldsymbol{\gamma}}$ (height for males) against $\widehat{\boldsymbol{\Gamma}}$ (height for females) with a sequence of p-value thresholds in the screening dataset.

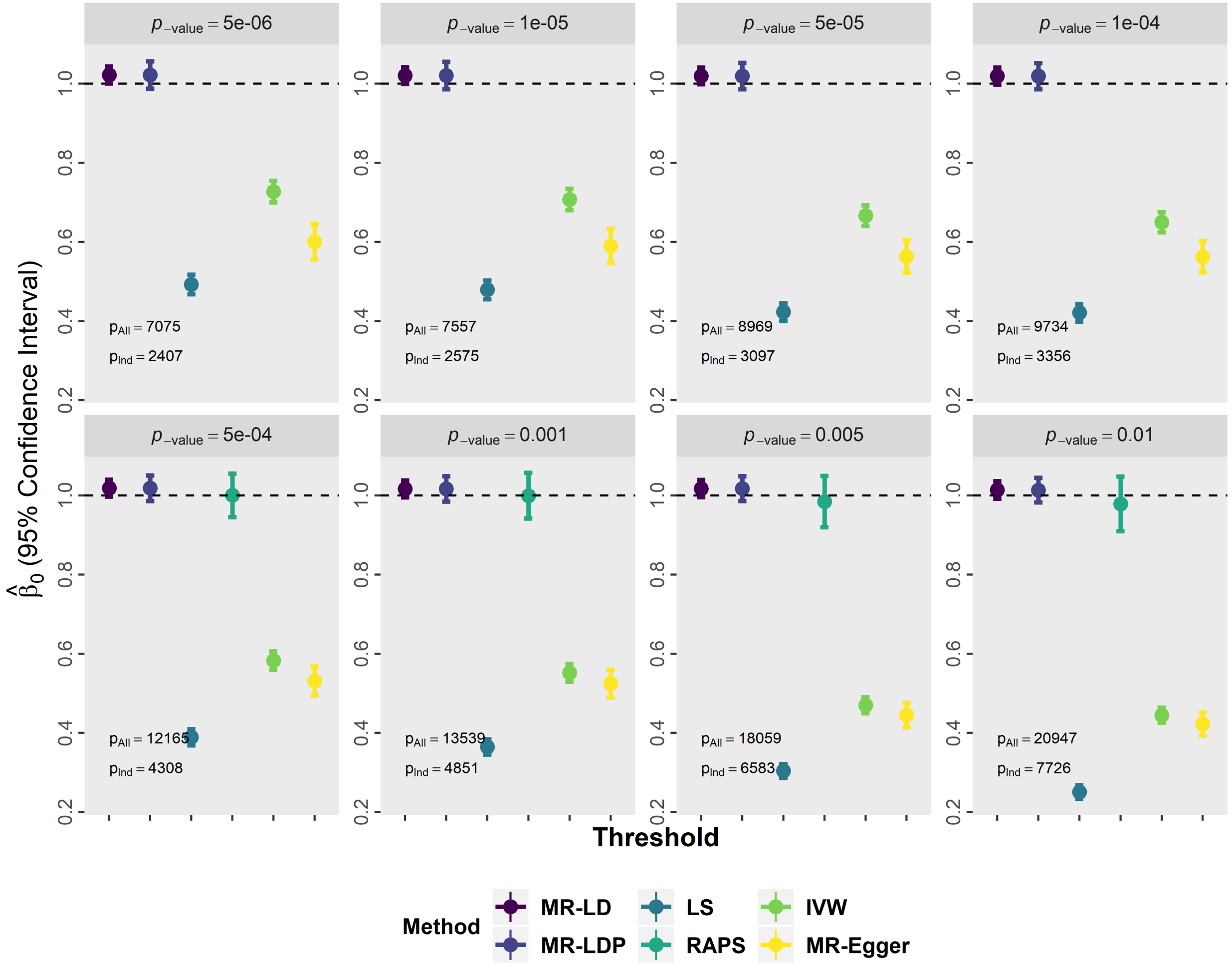

Figure S16: The result of Height-Height using UK10K as the reference penal with shrinkage parameter $\lambda = 0.1$ under different p-value thresholds to choose genetic variants in the screening dataset, e.g., p-value = 5e-6, 1e-05. MR-LD, MR-LDP and LS methods use all SNPs selected by the screening dataset (denoted as $\mathrm{P}_{\mathrm{All}}$), but IVW, MR-Egger, RAPS and GSMR use pruned SNPs, where the default value of r^2 is used for GSMR (the number of SNP used: $\mathrm{P}_{\mathrm{GSMR}}$) and $r^2 = 0.001$ is used for IVW, MR-Egger and RAPS (the number of SNP used: $\mathrm{P}_{\mathrm{Ind}}$).

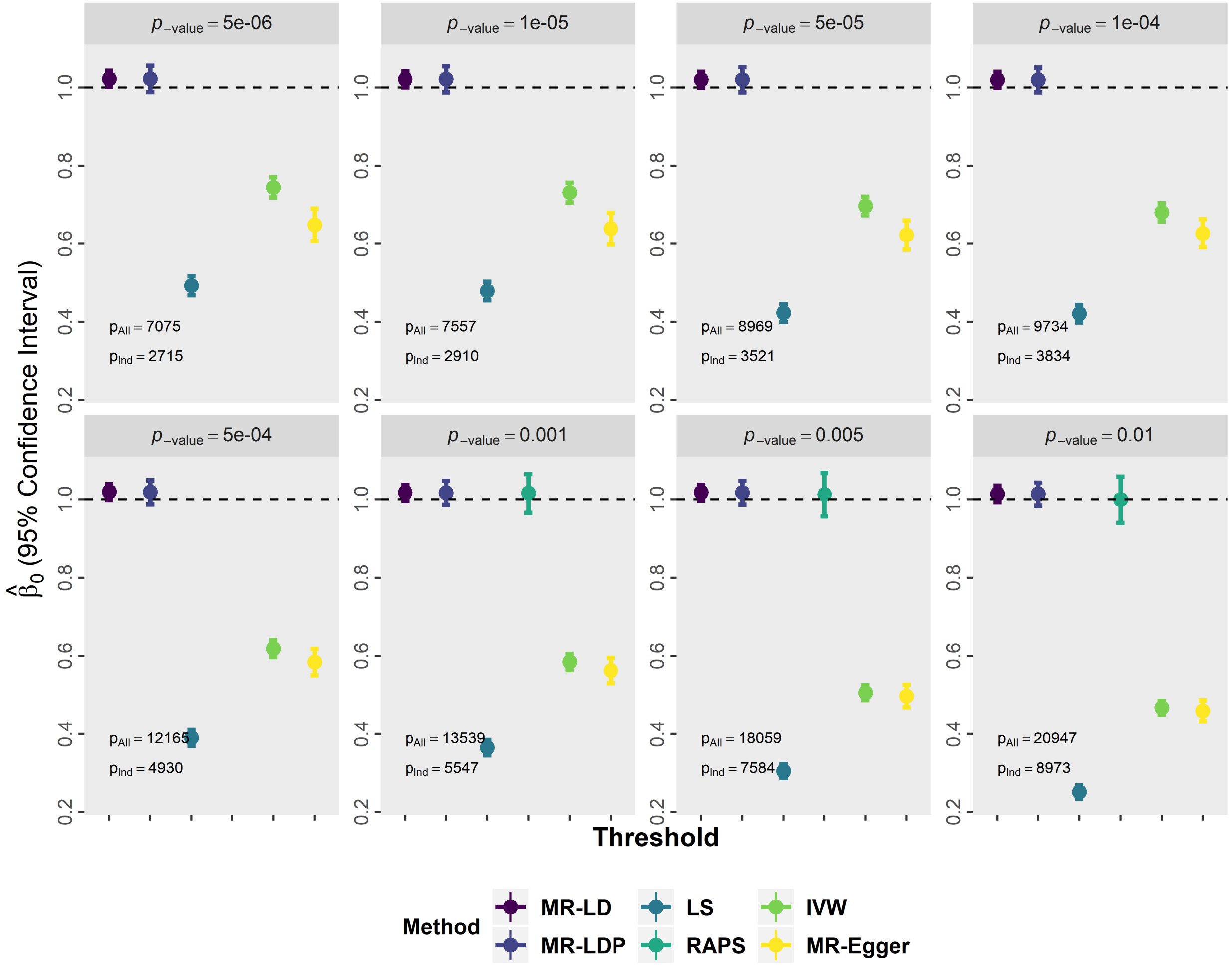

Figure S17: The result of Height-Height using UK10K as the reference penal with shrinkage parameter $\lambda = 0.15$ under different p-value thresholds to choose genetic variants in the screening dataset, e.g., p-value = 5e-6, 1e-05. MR-LD, MR-LDP and LS methods use all SNPs selected by the screening dataset (denoted as P_{All}), but IVW, MR-Egger, RAPS and GSMR use pruned SNPs, where the default value of r^2 is used for GSMR (the number of SNP used: P_{GSMR}) and $r^2 = 0.001$ is used for IVW, MR-Egger and RAPS (the number of SNP used: P_{Ind}).

4.2 Applications to the effect of lipids and BMI on common diseases

4.2.1 Lipids-disease outcome

The association result of lipids on common human diseases with shrinkage parameter $\lambda = 0.15$ is presented in Table S1, where the threshold for selecting instrumental variants in the screening dataset is set to 1×10^{-4}. Furthermore, we analysis the association of HDL-C on CAD1, CAD2, and PVD using a sequence of thresholds with different shrinkage parameters, the results are illustrated in Figures S18 – S22.

Lipids	Outcome	P_{All}	MR-LDP	P_{GSMR}	GSMR	P_{Ind}	Raps	IVW	MR-Egger
HDL-C	CAD1	2104	-0.16(0.032)	299	-0.27(0.036)	222	-0.38(0.065)	-0.36(0.064)	-0.26(0.139)
	CAD2	2071	-0.09(0.023)	307	-0.05(0.029)	226	-0.11(0.045)	-0.12(0.043)	-0.01(0.088)
	MDD	2071	-0.06(0.031)	310	-0.14(0.038)	226	-0.13(0.056)	-0.13(0.053)	-0.11(0.109)
	T2D	2071	-0.14(0.036)	304	-0.19(0.042)	226	-0.3(0.073)	-0.31(0.074)	0.07(0.149)
	Dyslid	2071	-0.17(0.027)	283	-0.1(0.029)	226	-0.27(0.082)	-0.27(0.072)	-0.23(0.148)
	Hyper	2071	-0.09(0.02)	301	-0.16(0.022)	226	-0.2(0.035)	-0.2(0.035)	-0.08(0.07)
	PVD	2071	-0.13(0.055)	308	-0.13(0.075)	226	-0.18(0.1)	-0.17(0.097)	0.08(0.199)
	DC	2071	-0.06(0.011)	300	-0.08(0.013)	226	-0.08(0.023)	-0.09(0.023)	-0.02(0.046)
LDL-C	CAD1	1863	0.38(0.031)	283	0.45(0.034)	211	0.4(0.051)	0.38(0.051)	0.43(0.09)
	CAD2	1816	0.16(0.023)	294	0.2(0.026)	215	0.16(0.036)	0.15(0.037)	0.21(0.058)
	T2D	1816	-0.07(0.032)	290	-0.1(0.037)	215	-0.05(0.054)	-0.05(0.057)	-0.06(0.092)
	Dyslid	1816	0.75(0.031)	286	0.96(0.026)	215	0.93(0.042)	0.9(0.041)	0.98(0.064)
	Hyper	1816	0.05(0.018)	289	0.06(0.019)	215	0.04(0.028)	0.04(0.029)	0.05(0.045)
	Osteoa	1816	-0.05(0.025)	295	-0.05(0.028)	215	-0.08(0.036)	-0.07(0.036)	-0.07(0.057)
	DC	1816	0.11(0.01)	294	0.14(0.011)	215	0.13(0.016)	0.12(0.016)	0.16(0.026)
TC	CAD1	2516	0.37(0.031)	334	0.49(0.035)	239	0.48(0.048)	0.46(0.05)	0.47(0.104)
	CAD2	2454	0.15(0.023)	346	0.2(0.027)	240	0.19(0.034)	0.18(0.034)	0.21(0.063)
	Dyslid	2454	0.79(0.033)	332	1.1(0.028)	240	1.01(0.046)	0.98(0.042)	1.1(0.075)
	Osteoa	2454	-0.06(0.026)	347	-0.05(0.03)	240	-0.17(0.04)	-0.16(0.04)	-0.14(0.073)
	DC	2454	0.1(0.011)	345	0.14(0.012)	240	0.12(0.017)	0.12(0.017)	0.14(0.031)

Table S1: Causal associations of lipids on common diseases using UK10K as the reference penal with the shrinkage parameter $\lambda = 0.15$. MR-LDP uses all SNPs selected by the screening dataset (denoted as P_{All}), but IVW, MR-Egger, RAPS and GSMR use pruned SNPs, where the default value of r^2 is used for GSMR (the number of SNP used: P_{GSMR}) and $r^2 = 0.001$ is used for IVW, MR-Egger and RAPS (the number of SNP used: P_{Ind}). Statistically significant results are indicated in blue.

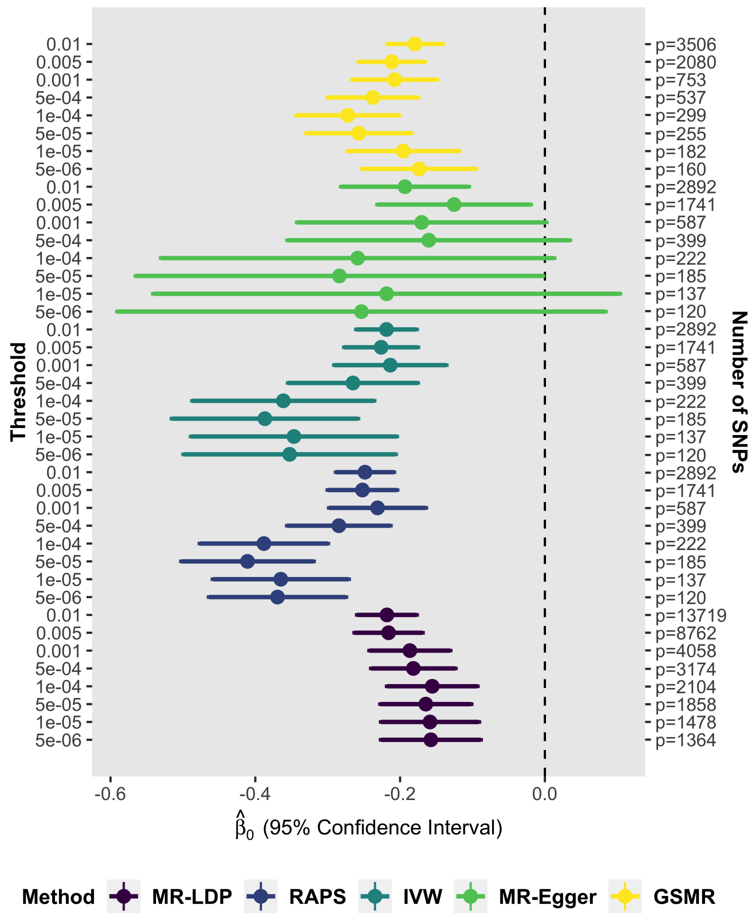

Figure S18: Causal associations of HDL-C on CAD1 under different p-value thresholds in the screening dataset, where UK10K was used as the reference panel and the shrinkage parameter $\lambda = 0.15$.

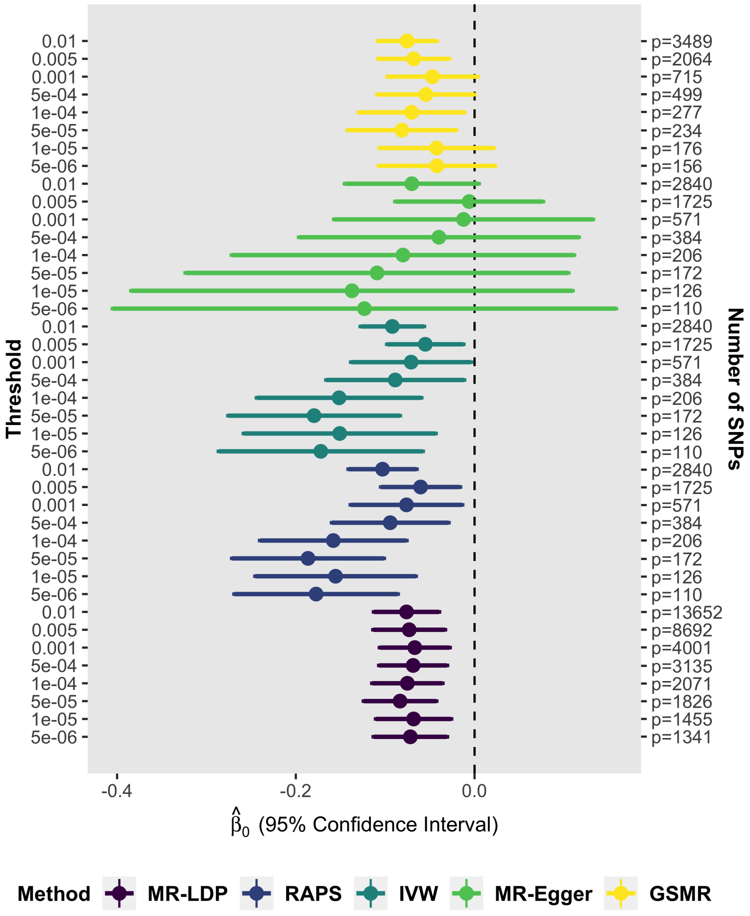

Figure S19: Causal associations of HDL-C on CAD2 under different p-value thresholds in the screening dataset, where UK10K was used as the reference panel and the shrinkage parameter $\lambda = 0.1$.

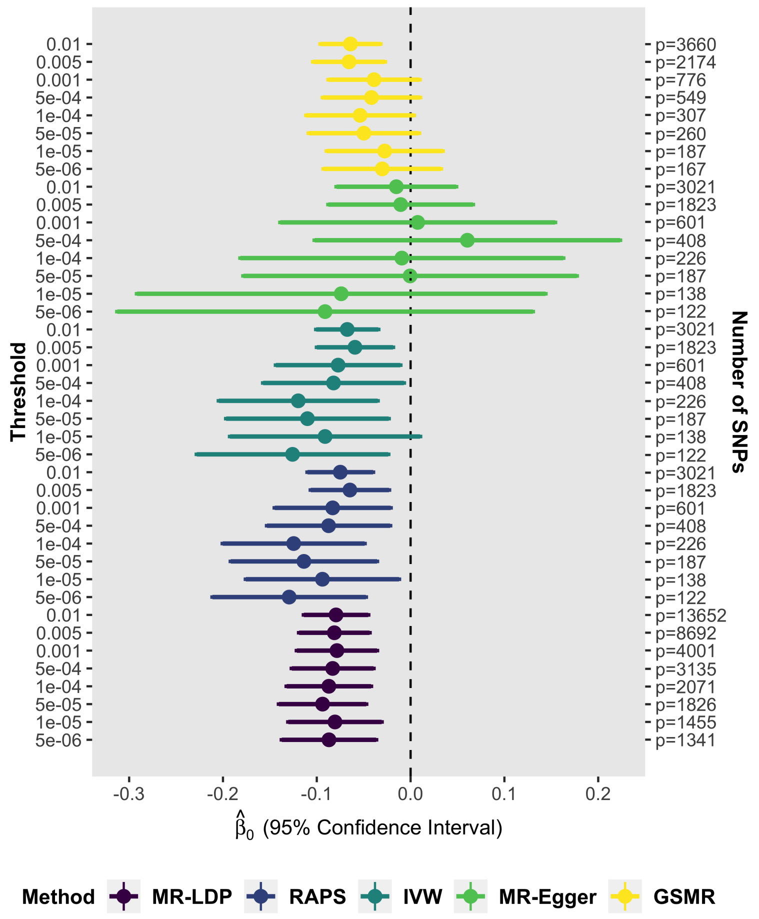

Figure S20: Causal associations of HDL-C on CAD2 under different p-value thresholds in the screening dataset, where UK10K was used as the reference panel and the shrinkage parameter $\lambda = 0.15$.

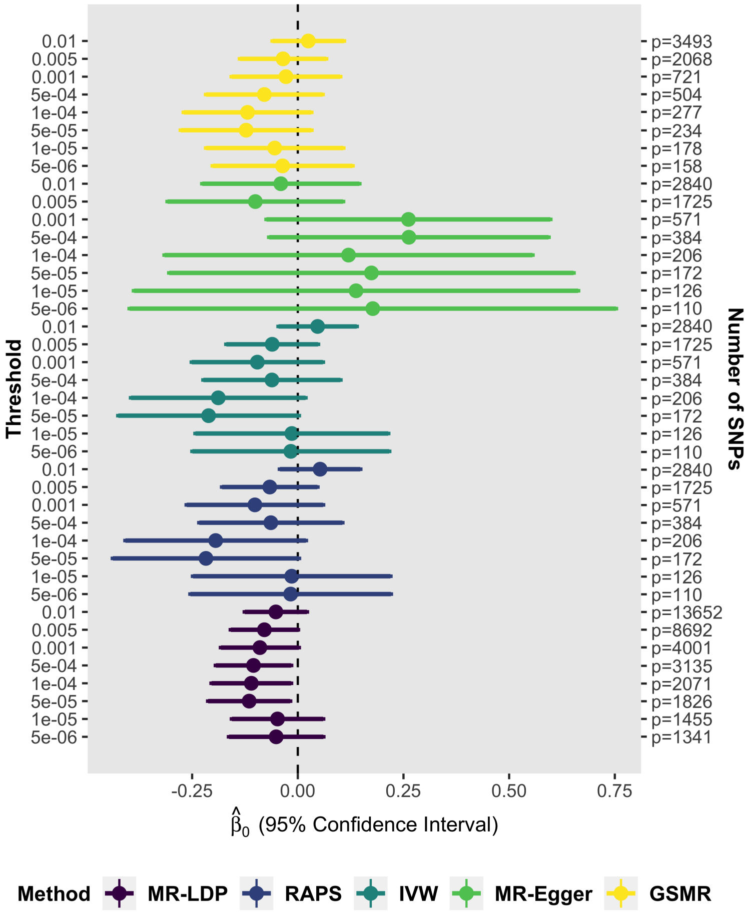

Figure S21: Causal associations of HDL-C on PVD under different p-value thresholds in the screening dataset, where UK10K was used as the reference panel and the shrinkage parameter $\lambda = 0.1$.

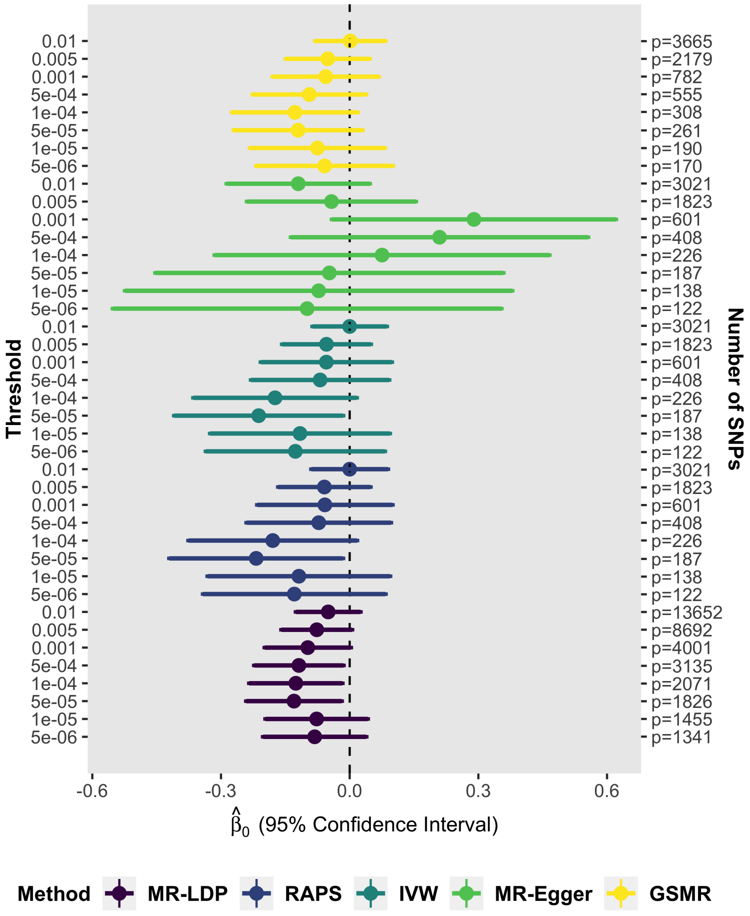

Figure S22: Causal associations of HDL-C on PVD under different p-value thresholds in the screening dataset, where UK10K was used as the reference panel and the shrinkage parameter $\lambda = 0.15$.

4.2.2 BMI-disease outcome

Similarly, the association result of BMI on common human diseases with shrinkage parameter $\lambda = 0.15$ is showed in Table S2, where the threshold for selecting instrumental variants in the screening dataset is set to 1×10^{-4}. We also analyze the association of BMI on hemorrhoids and PVD using a sequence of thresholds with different shrinkage parameters, the results are illustrated in Figures S23 – S26.

Outcome	P_{All}	MR-LDP	P_{GSMR}	GSMR	P_{Ind}	RAPS	IVW	MR-Egger
CAD1	4403	0.2(0.08)	745	0.32(0.068)	594	0.25(0.116)	0.21(0.088)	0.19(0.128)
Asthma	4426	0.28(0.069)	748	0.24(0.059)	596	0.23(0.104)	0.19(0.077)	0.18(0.113)
CAD2	4426	0.22(0.064)	749	0.22(0.06)	596	0.19(0.101)	0.15(0.076)	0.08(0.111)
T2D	4426	0.86(0.133)	748	0.72(0.093)	596	1.12(0.158)	0.85(0.12)	1.18(0.175)
Dyslid	4426	0.22(0.072)	745	0.28(0.057)	596	0.12(0.125)	0.12(0.083)	0.21(0.121)
Hemorrhoids	4426	0.31(0.128)	750	0.24(0.108)	596	0.19(0.165)	0.15(0.124)	0(0.182)
Hyper	4426	0.47(0.063)	744	0.47(0.045)	596	0.58(0.092)	0.45(0.066)	0.53(0.097)
Insomnia	4426	0.78(0.226)	749	0.87(0.21)	596	1.16(0.312)	0.9(0.238)	0.62(0.348)
Osteoa	4426	0.27(0.075)	750	0.28(0.067)	596	0.29(0.106)	0.22(0.079)	0.4(0.115)
Osteop	4426	-0.44(0.17)	750	-0.39(0.146)	596	-0.53(0.232)	-0.41(0.173)	-0.52(0.253)
PVD	4426	0.35(0.161)	750	0.36(0.155)	596	0.25(0.231)	0.19(0.179)	0.29(0.261)
DC	4426	0.27(0.033)	741	0.29(0.027)	596	0.28(0.049)	0.22(0.036)	0.23(0.052)

Table S2: Causal associations of BMI on common diseases using UK10K as the reference penal with the shrinkage parameter $\lambda = 0.15$. MR-LDP uses all SNPs selected by the screening dataset (denoted as P_{All}), but IVW, MR-Egger, RAPS and GSMR use pruned SNPs, where the default value of r^2 is used for GSMR (the number of SNP used: P_{GSMR}) and $r^2 = 0.001$ is used for IVW, MR-Egger and RAPS (the number of SNP used: P_{Ind}). Statistically significant results are indicated in blue.

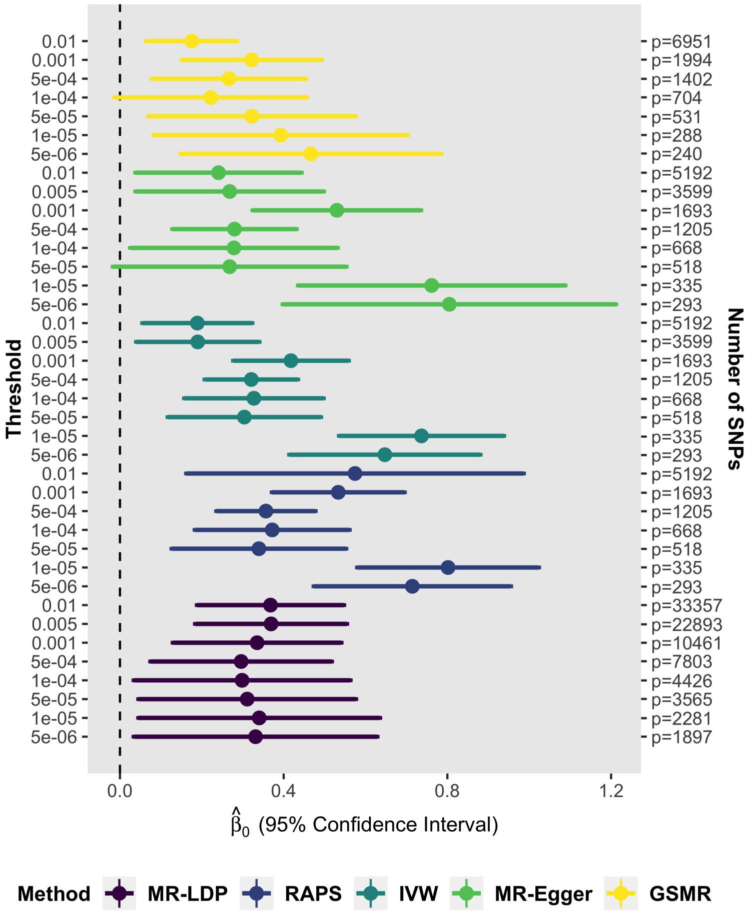

Figure S23: Causal associations of BMI on Hemorrhoids under different p-value thresholds in the screening dataset, where UK10K was used as the reference panel and the shrinkage parameter $\lambda = 0.1$.

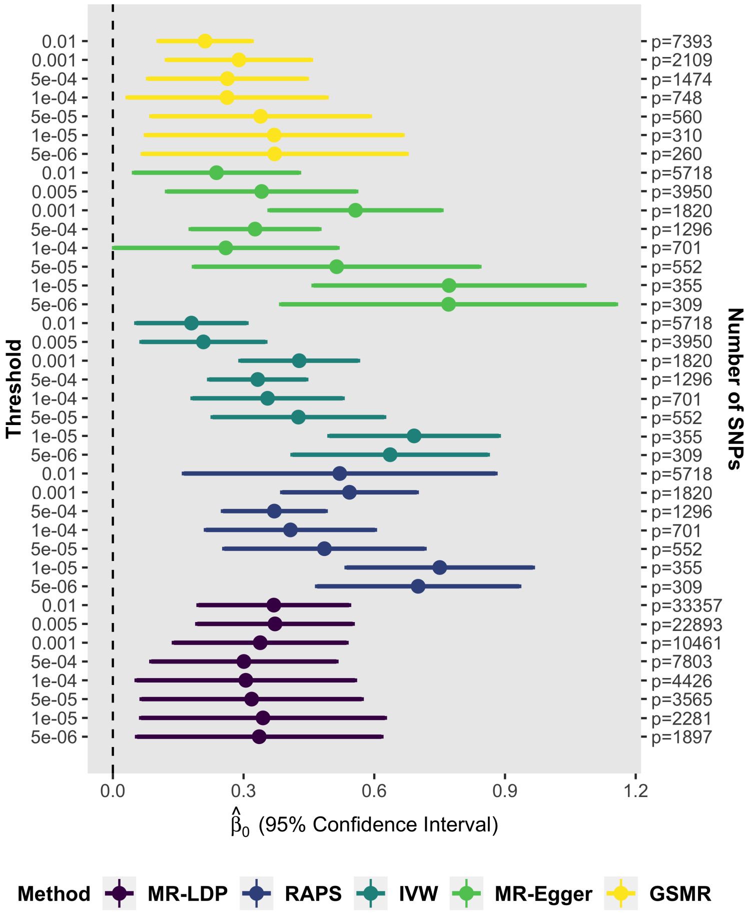

Figure S24: Causal associations of BMI on Hemorrhoids under different p-value thresholds in the screening dataset, where UK10K was used as the reference panel and the shrinkage parameter $\lambda = 0.15$.

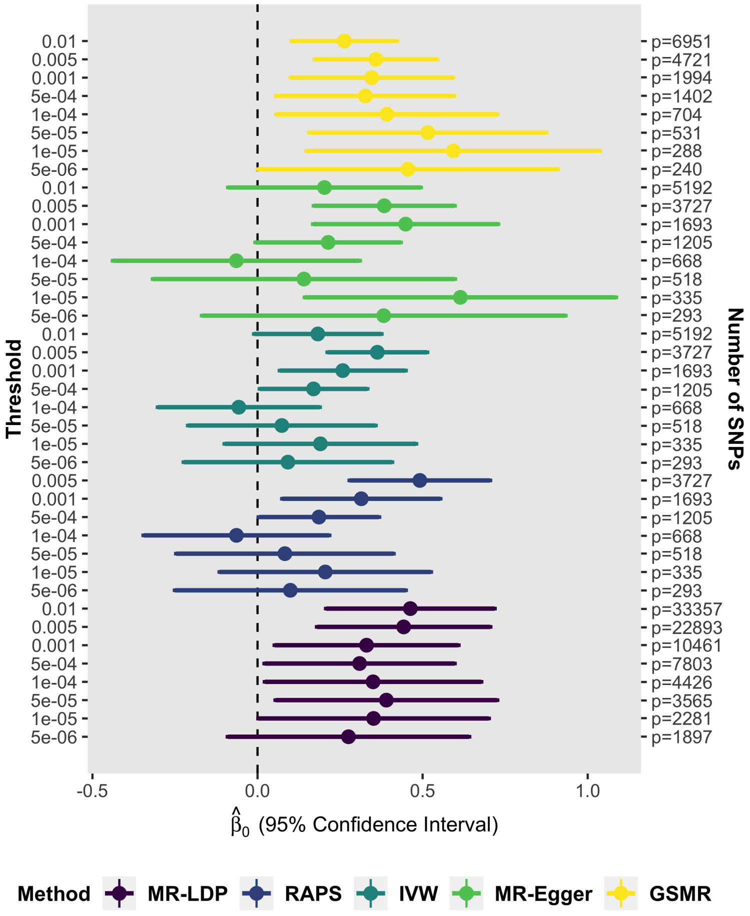

Figure S25: Causal associations of BMI on PVD under different p-value thresholds in the screening dataset, where UK10K was used as the reference panel and the shrinkage parameter $\lambda = 0.1$.

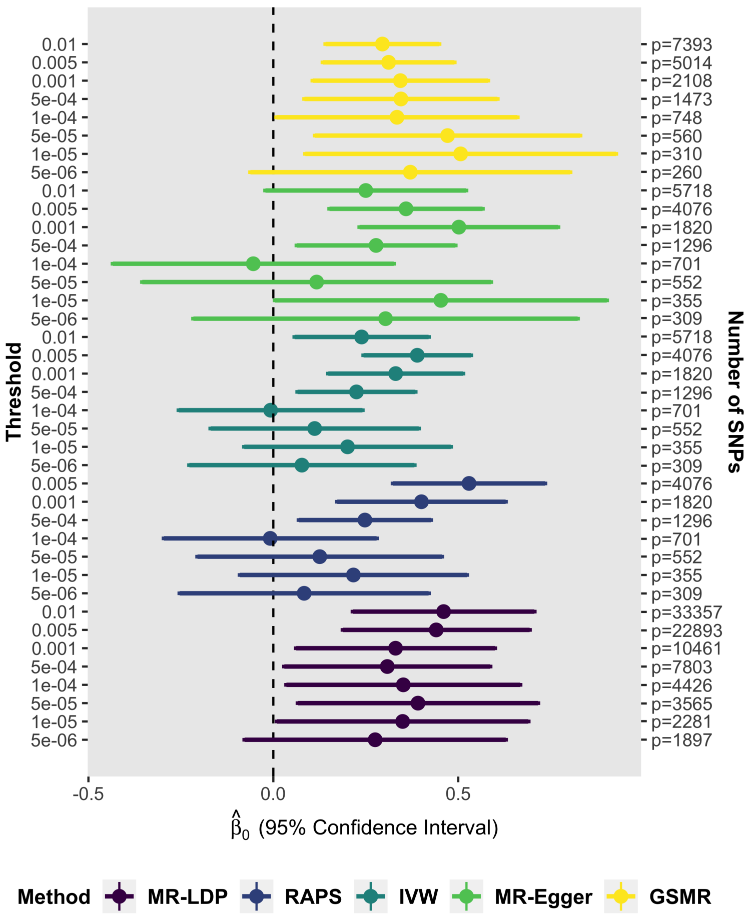

Figure S26: Causal associations of BMI on PVD under different p-value thresholds in the screening dataset, where UK10K was used as the reference panel and the shrinkage parameter $\lambda = 0.15$.

5 The detail information of GWAS Datasets

Tables S3 and S4 summarize the total number of SNPs and sample sizes for each trait, health risk factor or disease outcome and indicate the source details for these GWAS summary statistics.

ID	Year	Website
MI (screen)	2018	http://geneatlas.roslin.ed.ac.uk/downloads/
C4D	2011	http://www.cardiogramplusc4d.org/data-downloads
CAD1	2011	http://www.cardiogramplusc4d.org/data-downloads
CAD2	2018	http://cnsgenomics.com/data.html
Height (screen)	2018	http://geneatlas.roslin.ed.ac.uk/downloads/
Height-M	2013	https://portals.broadinstitute.org/collaboration/giant/index.php/Main_Page
Height-F	2013	https://portals.broadinstitute.org/collaboration/giant/index.php/Main_Page
BMI-JAP (screen)	2017	ftp://ftp.ebi.ac.uk/pub/databases/gwas/summary_statistics/AkiyamaM_28892062_GCST004904
BMI	2015	https://portals.broadinstitute.org/collaboration/giant/index.php/Main_Page
HDL-C (screen)	2010	http://csg.sph.umich.edu/willer/public/lipids2010/
LDL-C (screen)	2010	http://csg.sph.umich.edu/willer/public/lipids2010/
TC (screen)	2010	http://csg.sph.umich.edu/willer/public/lipids2010/
HDL-C (exposure)	2013	http://csg.sph.umich.edu/willer/public/lipids2013/
LDL-C (exposure)	2013	http://csg.sph.umich.edu/willer/public/lipids2013/
TC (exposure)	2013	http://csg.sph.umich.edu/willer/public/lipids2013/
Asthma	2018	http://cnsgenomics.com/data.html
AR	2018	http://cnsgenomics.com/data.html
Cancer	2018	http://cnsgenomics.com/data.html
MDD	2018	http://cnsgenomics.com/data.html
T2D	2018	http://cnsgenomics.com/data.html
Dyslid	2018	http://cnsgenomics.com/data.html
Hyper	2018	http://cnsgenomics.com/data.html
Hemorrhoids	2018	http://cnsgenomics.com/data.html
Hernia abdominopelvic cavity	2018	http://cnsgenomics.com/data.html
Insomnia	2018	http://cnsgenomics.com/data.html
IDA	2018	http://cnsgenomics.com/data.html
IBS	2018	http://cnsgenomics.com/data.html
Macular degeneration	2018	http://cnsgenomics.com/data.html
Osteoa	2018	http://cnsgenomics.com/data.html
Osteop	2018	http://cnsgenomics.com/data.html
PVD	2018	http://cnsgenomics.com/data.html
PU	2018	http://cnsgenomics.com/data.html
Pychiatric disorder	2018	http://cnsgenomics.com/data.html
Stress	2018	http://cnsgenomics.com/data.html
VV	2018	http://cnsgenomics.com/data.html
DC	2018	http://cnsgenomics.com/data.html

Table S3: The website of publicly available summary datasets used in this paper.

ID	Trait	Consortium	sample size	#SNP
MI (screen)	Myocardial infarction	UK BioBank	30,358	1,191,981
C4D	Coronary artery disease [8]	C4D Genetics Consortium	30,442	540,232
CAD1	Coronary artery disease[13]	CARDIoGRAM	86,995	2,420,360
CAD2	Coronary artery disease	UK BioBank	108, 039	1,162,292
Height (screen)	Height	UK BioBank	500,062	143,674
Height-M	Height in male in European group	[12]	270,000	1,181,512
Height-F	Height in female in European group	[12]	270,000	1,180,713
BMI-JAP (screen)	Body Mass Index[1]	[1]	173,430	5,961,105
BMI	Body Mass Index[11] in European group	Genetic Investigation of ANthropometric Traits(GIANT)	322,154	1,089,729
HDL-C (screen)	High-density lipoprotein cholesterol[14]	[14]	∼100,000	1,153,314
LDL-C (screen)	Low-density lipoprotein cholesterol[14]	[14]	∼100,000	1,153,337
TC (screen)	Total cholesterol[14]	[14]	∼100,000	1,153,322
HDL-C (exposure)	High-density lipoprotein cholesterol[15]	Global Lipids Genetics Consortium(GLGC)	188,577	2,447,441
LDL-C (exposure)	Low-density lipoprotein cholesterol[15]	Global Lipids Genetics Consortium(GLGC)	188,577	2,437,751
TC (exposure)	Total cholesterol[15]	Global Lipids Genetics Consortium(GLGC)	188,577	2,446,981
Asthma	Asthma	UK BioBank	108, 039	1,162,292
AR	Allergic rhinitis	UK BioBank	108, 039	1,162,292
Cancer	Cancer	UK BioBank	108,039	1,162,292
MDD	Major depression disorder	UK BioBank	108,039	1,162,292
T2D	type 2 diabetes	UK BioBank	108,039	1,162,291
Dyslid	Dyslipidemia	UK BioBank	108,039	1,162,292
Hyper	Hypertension disease	UK BioBank	108,039	1,162,292
Hemorrhoids	Hemorrhoids	UK BioBank	108,039	1,162,292
Hernia abdominopelvic cavity	hernia abdominopelvic cavity	UK BioBank	108,039	1,162,292
Insomnia	Insomnia	UK BioBank	108,039	1,063,186
IDA	Iron deficiency anemias	UK BioBank	108,039	1,063,186
IBS	Irritable bowel syndrome	UK BioBank	108,039	1,162,292
Macular degeneration	Macular degeneration	UK BioBank	108,039	1,063,186
Osteoa	Osteoarthritis	UK BioBank	108,039	1,162,292
Osteop	Osteoporosis	UK BioBank	108,039	1,162,292
PVD	Peripheral vascular disease	UK BioBank	108,039	1,063,186
PU	Peptic ulcer	UK BioBank	108,039	1,162,284
Pychiatric disorder	Psychiatric disorder	UK BioBank	108,039	1,162,292
Stress	acute reaction to stress	UK BioBank	108,039	1,063,186
VV	varicose veins	UK BioBank	108,039	1,162,293
DC	disease count	UK BioBank	108,039	1,162,284

Table S4: The Consortia of publicly available summary datasets used in this paper.

6 The vignette of R package: *MR.LDP*

6.1 Introduction

This vignette provides an introduction to the *MR.LDP* package which can be installed with the following commands:

```
library(devtools);
install_github("QingCheng0218/MR.LDP");
```

Load the package using the following command:

```
library(MR.LDP);
```

6.2 Fit MR-LDP using simulated data

We first generate genotype data using function *genRawGeno*:

```
library("mvtnorm");
library("PDSCE");
set.seed(2019);
rho = 0.4;L = 1; M = 50; p = M*L; m = p; Alrate = 1;
n1 = 20000; n2 = 20000; n3 = 2500; lam = 0.055;
maf = runif(p, 0.05, 0.5);
G = genRawGeno(maf, L, M, rho, n1 + n2 + n3);
G1 = G[1:n1,];
G2 = G[(n1+1):(n1+n2),];
G12 = G[1:(n1+n2),];
G3 = G[(n1+n2+1):(n1+n2+n3),];
```

Estimate the covariance matrix using function *pdsoft*:

```
R0 = cor(G3);
R = pdsoft(R0, lam)$theta;
diag(R) = rep(1, p);
mask = kronecker(diag(L),matrix(1,M,M));
R = R*mask;
```

Generate the exposure data(y) and outcome data(z) with prespecified indirect(h_y^2) and direct(h_z^2) heritability based on

$$\mathbf{y} = \mathbf{G}_1\gamma + \mathbf{U}_x\eta_x + \mathbf{e}_1, \quad \mathbf{z} = \beta_0\mathbf{x} + \mathbf{G}_2\alpha + \mathbf{U}_y\eta_y + \mathbf{e}_2,$$

```
h2z <- 0.05; h2y <- 0.1; b0 <- 0.1; q <- 50;
u = matrix(rnorm( (n1+n2) * q),ncol=q);

sigma2g <- 0.005;
gamma.nz = rnorm(m)*sqrt(sigma2g);
indx = sample(1:p,m);
gamma = numeric(p);
gamma[indx] = gamma.nz;

Su = matrix(c(1,0.8,0.8,1),nrow=2)
bu = rmvnorm(q,mean=rep(0,2), sigma = Su,method="chol")
by = bu[,1]; bz = bu[,2];
uby = u%*%by; ubz = u%*%bz;
uby = uby/sqrt(as.numeric(var(uby)/0.6));
ubz = ubz/sqrt(as.numeric(var(ubz)/0.2));

G12g = G12%*%gamma;

if(b0!=0){
h2ga = (h2y *( 1 + b0^2))/(b0^2 * (1 - h2y));
gamma0 = gamma/sqrt(as.numeric(var(G12g)/h2ga));
G12g = G12%*%gamma0;
}

yall = G12g + uby + rnorm(n1+n2)*as.numeric(sqrt(1-var(uby)));

# The direct effects on Z
h2yb = var(b0*yall);
h2al = (h2z + h2z*h2yb)/(1 - h2z)

sigma2a <- 0.005;
if(h2z==0){
alpha0 = rep(0, m);
G12a = G12%*%alpha0;
}else{
alno = floor(p*Alrate);
alpha.nz <- rnorm(alno)*sqrt(sigma2a);
# sparse setting for pleiotropy
indxAL = sample(1:p,alno);
alpha = numeric(p);
alpha[indxAL] = alpha.nz;

G12a = G12%*%alpha;
```

```
alpha0 = alpha/sqrt(as.numeric(var(G12a)/(h2al)));
G12a = G12%*%alpha0;
}

resz = ubz + rnorm(n1+n2)*as.numeric(sqrt(1-var(ubz)));
zall = b0*yall  + G12a +  resz;
H2a.res <- var(G12a)/var(zall);
H2g.res <- var(b0*G12g)/var(zall);

y = yall[1:n1];
z = zall[(n1+1):(n1+n2)];
```

We then conduct single-variant analysis to obtain the summary statistics.

```
gammah = numeric(p); Gammah = numeric(p);
segamma = numeric(p); seGamma = numeric(p);
pval = numeric(p);
for (i in 1:p){
fm = lm(y~1+G1[,i]);
gammah[i] = summary(fm)$coefficients[2,1];
segamma[i] = summary(fm)$coefficients[2,2];
pval[i] = summary(fm)$coefficients[2,4];

fm = lm(z~1+G2[,i]);
Gammah[i] = summary(fm)$coefficients[2,1];
seGamma[i] = summary(fm)$coefficients[2,2];
}
```

Until now, we obtain the summary statistics: **gammah** and **segamma** for esposure data, **Gammah** and **seGamma** for outcome data.

Initialize the parameters for MR-LDP algorithm. **epsStopLogLik** is the convergence tolerance, **maxIter** is the iteration number. **beta0, gamma, alpha, sgga2, sgal2** are the initial values for the PX-VBEM algorithm.

```
epsStopLogLik <- 1e-7; maxIter <- 10000;
beta0 <- 0;
gamma <- rep(0, p);
alpha <- rep(0, p);
sgga2 <- 0.01;
sgal2 <- 0.01;
```

We conduct the simulation study using *MRLDP_SimPXvb*, **model = 1** and **model = 2** represent MR-LD and MR-LDP, respectively.

Fit MR-LD w/ (**constr = 1**) and w/o (**constr = 0**) constraint (**constr**) that $\beta = 0$ as:

```
SimMRLD_Hb = MRLDP_SimPXvb(gammah, Gammah, segamma, seGamma, gamma,
alpha,  beta0, sgga2, sgal2, R, constr = 0,
epsStopLogLik, maxIter, model = 1);

SimMRLD_H0 = MRLDP_SimPXvb(gammah, Gammah, segamma, seGamma, gamma,
alpha,  beta0, sgga2, sgal2, R, constr = 1,
epsStopLogLik, maxIter, model = 1);

tstat = 2*(SimMRLD_Hb$tstat - SimMRLD_H0$tstat);
pval = pchisq(tstat, 1, lower.tail = F);
beta_hat = SimMRLD_Hb$beta0;
se_hat = abs(beta_hat/sqrt(tstat));
```

Fit MR-LDP w/ (**constr = 1**) and w/o (**constr = 0**) constraint that $\beta = 0$ as:

```
SimMRLDP_Hb = MRLDP_SimPXvb(gammah, Gammah, segamma, seGamma, gamma,
alpha,  beta0, sgga2, sgal2, R, constr = 0,
epsStopLogLik, maxIter, model = 2);

SimMRLDP_H0 = MRLDP_SimPXvb(gammah, Gammah, segamma, seGamma, gamma,
alpha,  beta0, sgga2, sgal2, R, constr = 1,
epsStopLogLik, maxIter, model = 2);

tstat = 2*(SimMRLDP_Hb$tstat - SimMRLDP_H0$tstat);
pval = pchisq(tstat, 1, lower.tail = F);
beta_hat = SimMRLDP_Hb$beta0;
se_hat = abs(beta_hat/sqrt(tstat));
```

beta_hat, se_hat, pval are estimated causal effect, corresponding standard error and p-value of **beta_hat**.

6.3 Fit MR-LDP using CAD-CAD study.

Furthermore, we give an example to illustrate the implements of MR.LDP for real data analysis. The following datasets("heart attack_myocardial infarction.txt", "c4d.txt", "cardiogram.txt", "all_chr_1000G.bed", "all_chr_1000G.fam", "all_chr_1000G.bim", "fourier_ls-

all.bed") should be prepared. Download here [https://drive.google.com/drive/folders/1IAs3daG9TIvjnneR32j1pfV0niz8PHyu].

```
filescreen= "heart attack_myocardial infarction.txt";
fileexposure = "c4d.txt";
fileoutcome = "cardiogram.txt";
stringname3 = "all_chr_1000G";
blockfile = "fourier_ls-all.bed"
```

filescreen, **fileexposure**, **fileoutcome** are the datasets names for screen, exposure and outcome, respectively. These three datasets must have the following format (note that it must be tab delimited):

SNP	chr	BP	A1	A2	beta	se	pvalue
rs3094315	1	752566	A	C	0.00012546	0.00042437	0.76750
rs3131969	1	754182	G	A	0.00033099	0.00045415	0.46611
rs3131972	1	752721	G	A	0.00010445	0.00042414	0.80548
rs1048488	1	760912	T	C	0.00017441	0.00042195	0.67935
rs12562034	1	768448	A	C	-0.00003632	0.00049399	0.94138
rs2286139	1	761732	T	C	0.00022796	0.00045149	0.61363

stringname3 is the name of reference panel data. Here we use samples from "1000 Genome Project Phase 1" which is in plink binary format. **blockfile** is used to partition the whole genome into blocks.

matchscreen function is used to match the four datasets with a cutoff named **pva_cutoff**. **matchExp = TRUE** means we fix the direction of exposure data. Since MR-LDP is invariant to the orientation of genetic variants, **matchExp** does not affect the results, default is FALSE.

```
pva_cutoff = 1e-4;
scrres = matchscreen(filescreen, fileexposure, fileoutcome, stringname3,
pva_cutoff, matchExp = FALSE)
bh1 = as.numeric(scrres$bh1);
bh2 = as.numeric(scrres$bh2);
s12 = as.numeric(scrres$s12);
s22 = as.numeric(scrres$s22);
```

```r
chr = as.numeric(scrres$chr);
bp = scrres$bp;
rsname = scrres$rsname
avbIndex = scrres$idxin;
idx4panel = scrres$idx4panel;
```

bh1 and **s12** are the SNP effects and corresponding standard errors on the exposure variable, **bh2** and **s22** are the SNP effects and corresponding standard errors on the outcome variable. After matching the four datasets, we obtain **chr** (chromosome number) , **bp** (base position), **rsname** (rs number). **avbIndex** (location) and **idx4panel** (Indicators to be adjusted in reference panel data).

One can using the following function *summaryQC* to remove the MHC region (**QCindex = 1**), or skip this procedure (**QCindex = 0**).

```r
QCindex = 1;
if(QCindex){
QCresult = summaryQC(mhcstart, mhcend, bh1, bh2, s12, s22, bp,
chr, rsname, avbIndex, idx4panel, Inf, Inf);
bh1new = QCresult$bh1new;
bh2new = QCresult$bh2new;
s12new = QCresult$s12new;
s22new = QCresult$s22new;
bpnew = QCresult$bpnew;
chrnew = QCresult$chrnew;
avbIndexnew = QCresult$avbIndexnew;
idx4panelnew = QCresult$idx4panel
rsnamenew = QCresult$rsnamenew;
}else{
bh1new = bh1;
bh2new = bh2;
s12new = s12;
s22new = s22;
bpnew = bp;
chrnew = chr;
rsnamenew = rsname;
idx4panelnew = idx4panel;
avbIndexnew = avbIndex;
}

p = length(avbIndexnew);
```

Initilize the parameters for MR-LDP algorithm. **CoreNum** is the number of cores in your CPU.

```
gamma = rep(0.01, p);
alpha = rep(0.01, p);
sgga2 =  0.01;
sgal2 =  0.01;
beta0 = 0;
maxIter = 10000
coreNum = 24;
lam = 0.1;
epsStopLogLik = 1e-7;
```

Fit MR-LD w/ (**constr** $=$ **1**) and w/o (**constr** $=$ **0**) constraint that $\beta = 0$ as:

```
RealMRLD_Hb = MRLDP_RealPXvb_block(bpnew, chrnew, avbIndexnew-1,
idx4panelnew, blockfile, stringname3, bh1new,
bh2new, s12new, s22new, gamma, alpha, beta0, sgga2,
sgal2, coreNum, lam, 0, epsStopLogLik, maxIter,
model = 1);

RealMRLD_H0 = MRLDP_RealPXvb_block(bpnew, chrnew, avbIndex-1,
idx4panelnew, blockfile, stringname3, bh1new,
bh2new, s12new, s22new, gamma, alpha, beta0, sgga2,
sgal2, coreNum, lam, 1, epsStopLogLik, maxIter,
model = 1);

beta0_MRLD = RealMRLD_Hb$beta0;
Tstat_LD <- 2*(RealMRLD_Hb$tstat - RealMRLD_H0$tstat);
MRLD_se = abs(RealMRLD_Hb$beta0/sqrt(Tstat_LD));
```

beta0_MRLD is the estimated effect of exposure on outcome and MRLD_se is corresponding standard error using MR-LD model.

Fit MR-LDP w/ (**constr** $=$ **1**) and w/o (**constr** $=$ **0**) constraint that $\beta = 0$ as:

```
RealMRLDP_Hb = MRLDP_RealPXvb_block(bpnew, chrnew, avbIndexnew-1,
idx4panelnew, blockfile, stringname3, bh1new,
bh2new, s12new, s22new, gamma, alpha, beta0, sgga2,
sgal2, coreNum, lam, 0, epsStopLogLik, maxIter,
model = 2);

RealMRLDP_H0 = MRLDP_RealPXvb_block(bpnew, chrnew, avbIndex-1,
idx4panelnew, blockfile, stringname3, bh1new,
```

```
bh2new, s12new, s22new, gamma, alpha, beta0, sgga2,
sgal2, coreNum, lam, 1, epsStopLogLik, maxIter,
model = 2);

beta0_MRLDP = RealMRLDP_Hb$beta0;
Tstat_MRLDP <- 2*(RealMRLDP_Hb$tstat - RealMRLDP_H0$tstat);
MRLDP_se = abs(RealMRLDP_Hb$beta0/sqrt(Tstat_MRLDP));
```

beta0_MRLDP is the estimated effect of exposure on outcome and MRLDP_se is the corresponding standard error using MR-LDP model.

References

1. Masato Akiyama, Yukinori Okada, Masahiro Kanai, Atsushi Takahashi, Yukihide Momozawa, Masashi Ikeda, Nakao Iwata, Shiro Ikegawa, Makoto Hirata, Koichi Matsuda, et al. Genome-wide association study identifies 112 new loci for body mass index in the japanese population. *Nature genetics*, 49(10):1458, 2017.

2. Christopher M Bishop. *Pattern recognition and machine learning.* springer, 2006.

3. David M Blei, Alp Kucukelbir, and Jon D McAuliffe. Variational inference: A review for statisticians. *Journal of the American Statistical Association*, 112(518):859–877, 2017.

4. Jack Bowden, George Davey Smith, and Stephen Burgess. Mendelian randomization with invalid instruments: effect estimation and bias detection through egger regression. *International journal of epidemiology*, 44(2):512–525, 2015.

5. Jack Bowden, Fabiola Del Greco M, Cosetta Minelli, George Davey Smith, Nuala Sheehan, and John Thompson. A framework for the investigation of pleiotropy in two-sample summary data mendelian randomization. *Statistics in medicine*, 36(11):1783–1802, 2017.

6. Evan A Boyle, Yang I Li, and Jonathan K Pritchard. An expanded view of complex traits: from polygenic to omnigenic. *Cell*, 169(7):1177–1186, 2017.

7. Raymond J Carroll, David Ruppert, Leonard A Stefanski, and Ciprian M Crainiceanu. *Measurement error in nonlinear models: a modern perspective.* Chapman and Hall/CRC, 2006.

8. Coronary Artery Disease (C4D) Genetics Consortium et al. A genome-wide association study in europeans and south asians identifies five new loci for coronary artery disease. *Nature genetics*, 43(4):339, 2011.

9. Vanessa Didelez and Nuala Sheehan. Mendelian randomization as an instrumental variable approach to causal inference. *Statistical methods in medical research*, 16(4):309–330, 2007.

10. Sander Greenland, James M Robins, and Judea Pearl. Confounding and collapsibility in causal inference. *Statistical science*, pages 29–46, 1999.

11. Adam E Locke, Bratati Kahali, Sonja I Berndt, Anne E Justice, Tune H Pers, Felix R Day, Corey Powell, Sailaja Vedantam, Martin L Buchkovich, Jian Yang, et al. Genetic studies of body mass index yield new insights for obesity biology. *Nature*, 518(7538):197, 2015.

12. Joshua C Randall, Thomas W Winkler, Zoltán Kutalik, Sonja I Berndt, Anne U Jackson, Keri L Monda, Tuomas O Kilpeläinen, Tõnu Esko, Reedik Mägi, Shengxu Li, et al. Sex-stratified genome-wide association studies including 270,000 individuals show sexual dimorphism in genetic loci for anthropometric traits. *PLoS genetics*, 9(6):e1003500, 2013.

13. Heribert Schunkert, Inke R König, Sekar Kathiresan, Muredach P Reilly, Themistocles L Assimes, Hilma Holm, Michael Preuss, Alexandre FR Stewart, Maja Barbalic, Christian Gieger, et al. Large-scale association analysis identifies 13 new susceptibility loci for coronary artery disease. *Nature genetics*, 43(4):333, 2011.

14. Tanya M Teslovich, Kiran Musunuru, Albert V Smith, Andrew C Edmondson, Ioannis M Stylianou, Masahiro Koseki, James P Pirruccello, Samuli Ripatti, Daniel I Chasman, Cristen J Willer, et al. Biological, clinical and population relevance of 95 loci for blood lipids. *Nature*, 466(7307):707, 2010.

15. Cristen J Willer, Ellen M Schmidt, Sebanti Sengupta, Gina M Peloso, Stefan Gustafsson, Stavroula Kanoni, Andrea Ganna, Jin Chen, Martin L Buchkovich, Samia Mora, et al.

Discovery and refinement of loci associated with lipid levels. *Nature genetics*, 45(11):1274, 2013.

16. Jian Yang, S Hong Lee, Michael E Goddard, and Peter M Visscher. Gcta: a tool for genome-wide complex trait analysis. *The American Journal of Human Genetics*, 88(1):76–82, 2011.

17. Yi Yang, Xingjie Shi, Yuling Jiao, Jian Huang, Min Chen, Xiang Zhou, Lei Sun, Xinyi Lin, Can Yang, and Jin Liu. Comm-s2: a collaborative mixed model using summary statistics in transcriptome-wide association studies. *bioRxiv*, 2019.

18. Qingyuan Zhao, Yang Chen, Jingshu Wang, and Dylan S Small. Powerful three-sample genome-wide design and robust statistical inference in summary-data mendelian randomization. *International journal of epidemiology*, 48(5):1478–1492, 2019.

19. Qingyuan Zhao, Jingshu Wang, Gibran Hemani, Jack Bowden, and Dylan S Small. Statistical inference in two-sample summary-data mendelian randomization using robust adjusted profile score. *arXiv preprint arXiv:1801.09652*, 2018.

20. Xiang Zhou and Matthew Stephens. Genome-wide efficient mixed-model analysis for association studies. *Nature genetics*, 44(7):821, 2012.

21. Xiang Zhou and Matthew Stephens. Efficient multivariate linear mixed model algorithms for genome-wide association studies. *Nature methods*, 11(4):407, 2014.
